# Transcriptome analysis and codominant markers development in caper, a drought tolerant orphan crop with medicinal value

**DOI:** 10.1038/s41598-019-46613-x

**Published:** 2019-07-18

**Authors:** Francesco Mercati, Ignazio Fontana, Alessandro Silvestre Gristina, Adriana Martorana, Mahran El Nagar, Roberto De Michele, Silvio Fici, Francesco Carimi

**Affiliations:** 1 Institute of Biosciences and BioResources (IBBR), National Research Council of Italy (CNR), Corso Calatafimi 414, 90129 Palermo, Italy; 2 Horticulture Department, Faculty of Agriculture, Benha University, 13736 Toukh, Egypt; 3 Dipartimento di Scienze Agrarie, Alimentari e Forestali (SAAF), Università di Palermo, Palermo, Italy

**Keywords:** Agricultural genetics, Plant genetics

## Abstract

Caper (*Capparis spinosa* L.) is a xerophytic shrub cultivated for its flower buds and fruits, used as food and for their medicinal properties. Breeding programs and even proper taxonomic classification of the genus *Capparis* has been hampered so far by the lack of reliable genetic information and molecular markers. Here, we present the first genomic resource for *C. spinosa*, generated by transcriptomic approach and *de novo* assembly. The sequencing effort produced nearly 80 million clean reads assembled into 124,723 unitranscripts. Careful annotation and comparison with public databases revealed homologs to genes with a key role in important metabolic pathways linked to abiotic stress tolerance and bio-compounds production, such purine, thiamine and phenylpropanoid biosynthesis, α-linolenic acid and lipid metabolism. Additionally, a panel of genes involved in stomatal development/distribution and encoding for Stress Associated Proteins (SAPs) was also identified. We also used the transcriptomic data to uncover novel molecular markers for caper. Out of 50 SSRs tested, 14 proved polymorphic and represent the first set of SSR markers for the genus *Capparis*. This transcriptome will be an important contribution to future studies and breeding programs for this orphan crop, aiding to the development of improved varieties to sustain agriculture in arid conditions.

## Introduction

Global warming is changing Earth’s climate with possible negative effects on the growth and reproductive success of plants. Reduced plant productivity due to environmental changes^1,2^, such as high temperatures, heat waves and drought stress, might implicate incapacity to ensure global food security^3,4^. The Mediterranean region will be particularly affected by climate change, with increased aridity expected to occur (Intergovernmental Panel on Climate Change; http://www.ipcc.ch)^5^. Mediterranean agriculture will need to adapt to the new environmental conditions by growing drought tolerant crops. Selection and introduction of stress-tolerant cultivars of existing crops is a slow and costly process, requiring intensive research and field trials^6^. Another option is promoting alternative drought resistant crop species. Caper (*Capparis spinosa* L.) is a xerophilous crop showing a remarkable adaptability to harsh environments, and with promising potentialities for agrosystems under the threat of global warming^7^.

In the Mediterranean area, populations are generally grouped within one species, *C. spinosa*^8–13^, although the taxonomic classification of this species is controversial due to a large pattern of morphological and ecological variations^14–17^ and to the lack of specific molecular markers. Despite the high polymorphism, two main subspecies, which differ ecologically and morphologically^11^ are recognized in Europe: *C*. *spinosa* subsp. *spinosa*, showing derived characters and widespread from the Mediterranean to Central Asia, and *C. spinosa* subsp. *rupestris* (Sm.) Nyman, characterized by phenotypic features close to the tropical stock of the group, distributed in the Mediterranean Region and the Sahara^13,17^.

The fruits and flower buds of caper are utilized as food ingredient, generally in brine, and appreciated for their flavor and texture. Only locally, cultivation of capers is extensive and acquires economic relevance for farmers. In particular, the main current areas of production are localised in Morocco, Turkey, Spain and Italy, namely in the minor islands of Salina and Pantelleria. Being rich in bio-active compounds, capers have many important medicinal properties^18–28^. Moreover, like other Mediterranean species^29–31^, caper is also a source of natural compounds with allelopathic potential^32,33^. Therefore, the extracts from *C. spinosa* could also be used to develop natural products employable in an eco-friendly agriculture.

For its interest as gourmet food, its medicinal and allelopathic properties and the ability to thrive in arid conditions^7^, capers have great agricultural potential in areas with increasing drought conditions, such as the Mediterranean basin. The process of domestication of caper plants has been limited and cultivated varieties are still very similar to wild accessions^34^, leaving ample margins for enhancement of many traits, such as increased productivity, firmer buds, disease resistance and thornless habit. Breeding programs and an efficient exploitation of this orphan crop are hampered by confused taxonomy of the genus *Capparis* and the lack of genomic information. To date, only few sequences of the chloroplast^35–37^ and mitochondrial genome^38^ have been reported, with limited value for phylogenetic analyses and breeding programs. Currently, there are no nuclear Simple Sequence Repeat (SSR) markers described for the genus *Capparis*. Microsatellites or SSR are codominant and highly informative markers already broadly used to genotype a wide range of plant species^39–43^. Compared to other molecular markers, SSRs are abundant and uniformly distributed throughout plant genomes and show several advantages such as simplicity, high polymorphism, reproducibility, co-dominant inheritance and cross-species transferability^44^. For species with no genome annotated, as is the case of orphan crops, an effective strategy to uncover SSRs is to rely on transcriptomic sequences. In contrast to genomic SSRs, Expressed Sequence Tag (EST)-SSRs are located in the coding and untranslated regions and are highly transferable to related taxa^45^. Thus, EST-SSR markers can directly influence phenotype and can be considered efficient functional markers^46^.

The advent of Next Generation Sequencing (NGS) technologies combined with bioinformatics tools can generate extensive data on non-model species in a very cost-effective way^47–49^. Among NGS strategies, RNA Sequencing (RNA-Seq) approach^50^ is a high throughput technology that has great advantages in examining the fine structure of a transcriptome^51,52^ and provides an effective way to obtain large amounts of sequence data without prior genome information^53–55^. RNA-Seq has been widely used in many organisms to obtain mass sequence data for transcriptional analysis, gene discovery and molecular marker development^52,54–56^, showing a great potential as a tool for molecular breeding^57^.

Here, for the first time, we report the sequencing, *de novo* assembly, and annotation of the leaf transcriptome of *C. spinosa* subsp*. rupestris*, a primitive type closer to the tropical stock of the group^13^. In order to identify putative genes controlling the bioactive and high-value components production the assembly was functionally annotated using public databases. In addition, polymorphic EST-SSRs were identified in the leaf transcriptome, thereby obtaining the first set of co-dominant markers for the species.

This transcript dataset provides the most widespread resource currently available for gene discovery and markers development in *C. spinosa*. This resource will be instrumental for future breeding programs and phylogenetic studies of capers. In addition, the information now available will contribute to the sustainable adaptation of agricultural production in small islands and marginal areas of the Mediterranean region^58^ and in other regions affected by aridity and/or climate change.

## Results

### Sequencing, de novo assembly and functional annotation of *C. spinosa* leaf transcriptome

**Figure 1:**
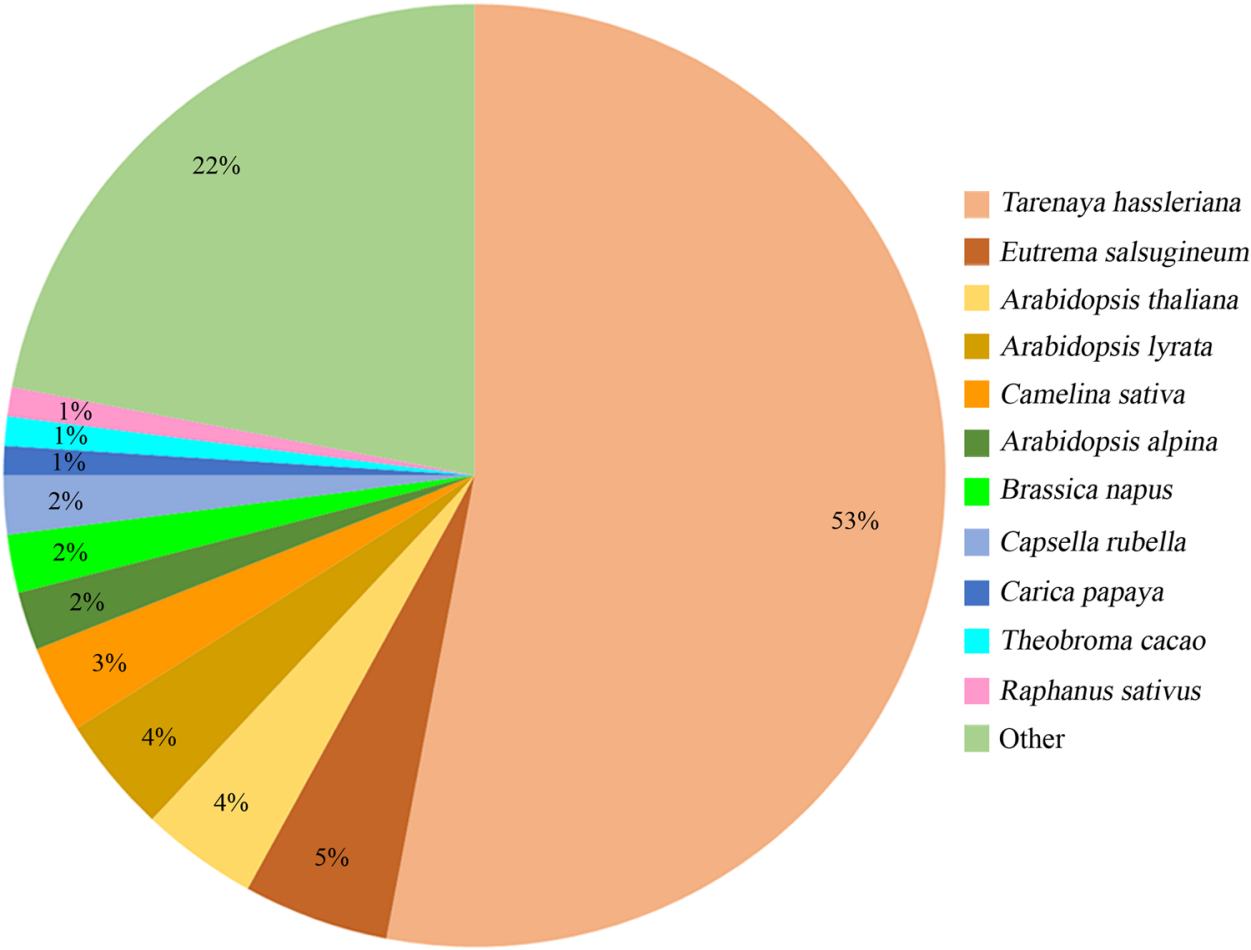
Species-based distribution of blastx matches for each clustered unitranscript of *Capparis spinosa* leaf transcriptome. The species with a match <1% were grouped in the ‘Other’ category.

We performed RNA-Seq to assemble transcripts, identify genes and develop co-dominant markers for the first time in *C. spinosa*. Leaf transcriptome Illumina shotgun sequencing yielded nearly 80 million cleaned reads, de novo assembled into 208,677 transcripts with N50 length of 2,431 bp (mean length 1,493 bp) by Trinity (Table 1). To remove the redundant transcripts the clean reads were clustered by CD-HIT-EST generating 124,723 unigenes with N50 length of 2,380 bp (mean 1,417 bp) (Table 1). The quality of assembled unitranscripts was evaluated by comparing them to the set of Eudicotyledons genes using BUSCO quality assessment tool. Out of the 2,121 BUSCO groups searched, 87.8% (1,861 BUSCOs) were “complete” (i.e., 916 single-copy and 945 duplicated), 6.7% (142 BUSCOs) were “fragmented” and the remaining 5.5% (118 BUSCOs) were “missing”. In addition, one typical peak of GC content for plants, (around 50%) was found using QUAST^59^, underlining the absence of bacteria. In total we identify 0.40% of possible contaminations (e.g. bacteria and virus), representing only 1.78% of the ‘Other’ category of Fig. 1. Clustered transcripts were searched against the NCBI-nr databases revealing 89,670 (72%) transcripts whose translation was significantly similar to known proteins. In species distribution analysis, 47,749 (53%) transcripts showed homology (top blast hits) with *Tarenaya hassleriana*, followed by *Eutrema salsugineum*, *Arabidopsis thaliana*, *Arabidopsis lyrata*, and *Camelina sativa* with 4,704 (5%), 3,322 (4%), 3,183 (4%) and 2,519 (3%), respectively (Fig. 1). The assembled sequences were also queried against the Swiss UniprotKB database using BLASTx and BLASTp searches, respectively. Nearly 54% (66,902) unitranscripts had a blastx hit and 41% (51,048) of clustered transcripts with ORF ≥ 100 bp in length displayed significant homology for annotated protein sequences. When nucleotide and protein sequences were aligned against UniRef90, their homology increased to 85,294 (68%) and 62,339 (50%), respectively. Moreover, 46,099 (37%) unique Pfam protein motifs could be assigned and 3,341 (3%) protein sequences were predicted to have signal peptides (Table 2). The complete list of transcript annotations is shown in Supplementary Dataset S1.

**Table 1:** Overview of sequencing outputs and assembly of *Capparis spinosa* leaf transcriptome.

Items	Trinity transcriptome assembly	Assembly after CD-HIT-EST clustering
Number of transcripts	208,677	124,723
Total size of transcripts	311,614,381	176,788,523
Longest contig (bp)	17,493	17,493
Shortest contig (bp)	201	201
Mean contig size (bp)	1,493	1,417
Median contig size (bp)	1,114	999
N50 contig length (bp)	2,431	2,380

**Table 2:** Overview of functional annotation by homology of *Capparis spinosa* leaf transcriptome.

Category	N° of unitranscripts
Predicted ORFs	104,505
Predicted proteins	64,541
Uniref90_Top_BLASTX_hit	85,294
sprot_Top_BLASTX_hit	66,902
Uniref90_Top_BLASTP_hit	62,339
sprot_Top_BLASTP_hit	51,048
Pfam	46,099
SignalP	3,341

ORFs, open reading frames;

Uniref90_Top_BLASTX_hit, top blastx hits against UniRef90 database;

sprot_Top_BLASTX_hit, top blastx hits against UniProtKB/Swiss-Prot database;

Uniref90_Top_BLASTP_hit, top blastp hits against UniRef90 database;

sprot_Top_BLASTP_hit, top blastp hits against UniProtKB/Swiss-Prot database;

Pfam, protein domain analysis was performed using http://www.sanger.ac.uk/software/pfam/;

SignalP, the presence of signal peptides was detected using http://www.cbs.dtu.dk/services/SignalP/.

**Figure 2:**
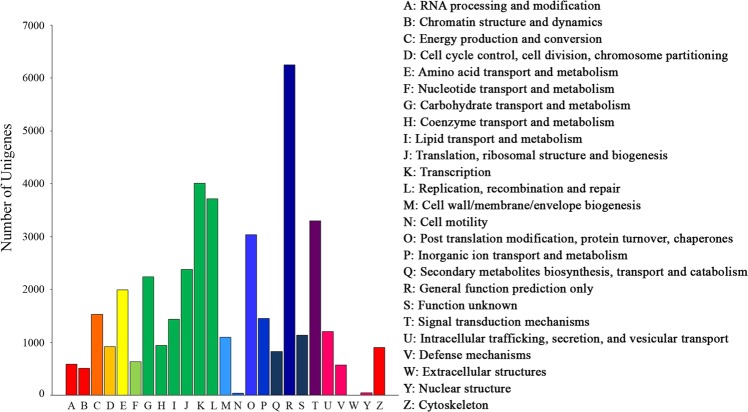
EuKaryotic Orthologous Groups (KOG) in *Capparis spinosa* leaf transcriptome. The unigenes with significant homologies in the KOG database were grouped into 24 categories. The number of unigenes belonging to each category was reported in the y-axis, while the subgroups in the KOG classification were represented in the x-axis.

We extracted 27,035 non redundant GO terms from 51% transcripts and summarized them into 97 GOslim plant categories using CateGOrizer (Supplementary Dataset S2). The annotated clustered transcripts were grouped into the three main categories: most of the assignments (61%) belonged to the biological process (BP) category, while the remaining was shared between cellular component (CC) (14%) and molecular function (MF) classes (25%). Within BP, “cellular process”, “metabolic process”, “cellular component organization and biogenesis” were the main represented groups in a total of 44 level-2 categories. Within CC, 26 level-2 categories were identified. The top three groups were “cell”, “intracellular” and “cytoplasm”. Similarly, in the MF 24 level-2 GO terms were isolated and “catalytic”, “transferase” and “hydrolase activities” were the top three (Supplementary Fig. S1). In the KOG classification, 40,765 unitranscripts were classified into 24 KOG groups (Fig. 2). Among these, the cluster for “general function prediction only” (15%) represented the largest group, followed by “transcription” (10%), “replication, recombination and repair” (9%), “signal transduction mechanism” (8%). The “cell motility” was the smallest group, while no unigenes were classified as “extracellular structures” (Fig. 2).

### Biological pathway analyses in *C. spinosa*

To investigate functional biological pathways in *C. spinosa*, we exploited Transdecoder that assigns KO to unitranscripts (e-value ≤ 1*10^−5^). The unique KOs identified were mapped against the KEGG database to verify the correct sequencing of well represented pathways in *C. spinosa*. Among the 127 KEGG pathways identified (Supplementary Table S1), purine metabolism (669 sequences; 76 KOs, covering 37% of the pathway), was the most represented pathway as number of homologous leaf transcripts. Pyrimidine metabolism (504; 56, covering 57%), oxidative phosphorylation (414; 60, covering 28%), phenylpropanoid biosynthesis (316; 18, covering 49%), fatty acid metabolism (biosynthesis and degradation) (297; 22, covering 23%) and α-linolenic acid metabolism (163; 12) were also highly represented.

**Figure 3:**
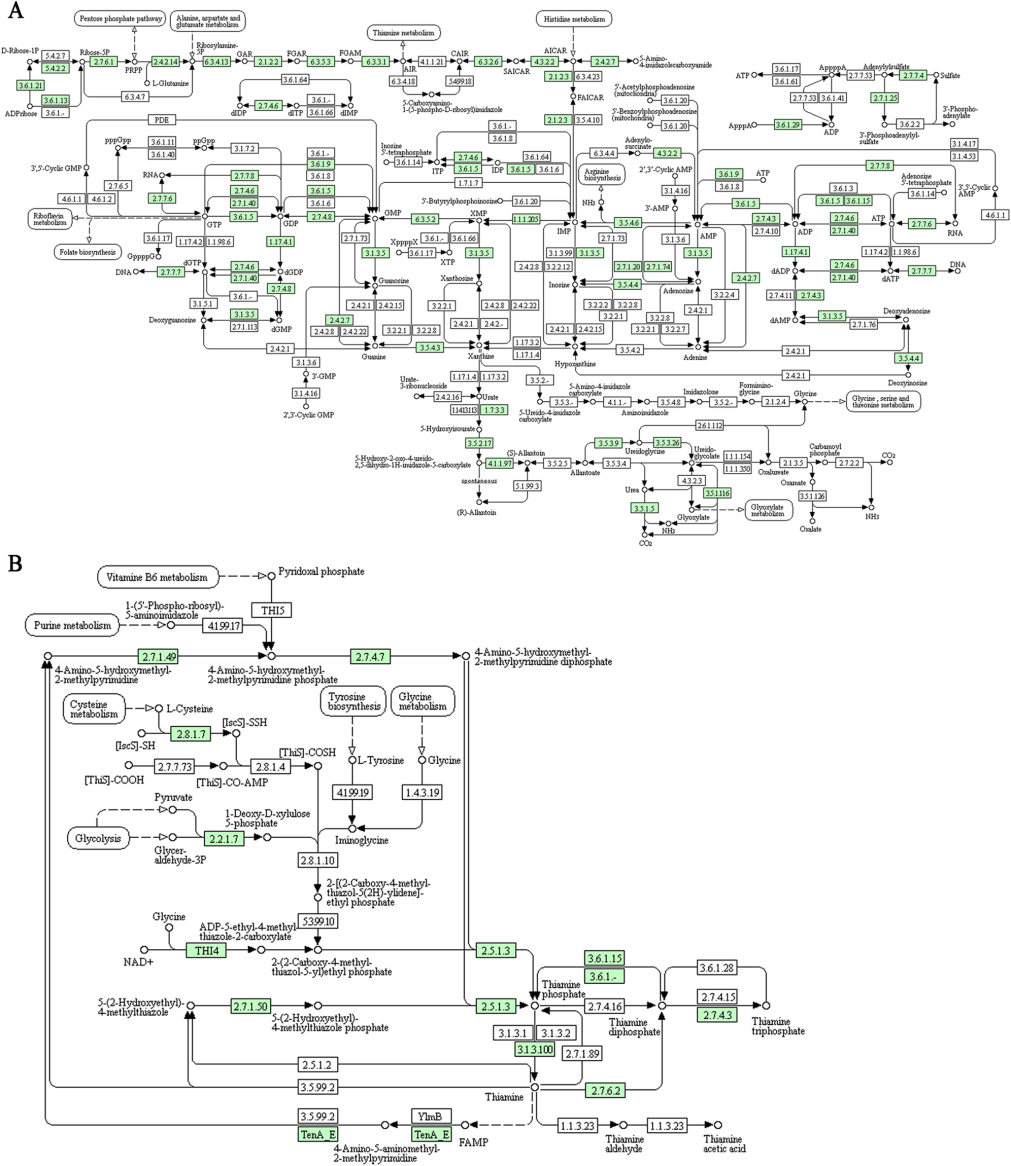
Analysis of purine (**A**) and thiamine (**B**) metabolism pathways by KEGG, showing the identified enzymes in *Capparis spinosa* leaf transcriptome (Enzyme Code - EC - identified are in green).

Because of their high representation and the known role of adenine, jasmonate, and flavonols in the abiotic stress tolerance^60–64^, we analyzed purine, thiamine and α-linolenic acid metabolism and phenylpropanoid biosynthesis in detail. In the *C. spinosa* leaf transcriptome, a high representation of purine metabolism was highlighted (Fig. 3A). Particularly, we found enzymes involved in the production of thiamine phosphates: thiamine-phosphate synthase (EC 2.5.1.3) catalyzing the reaction for thiamine phosphate synthesis, thiamine phosphatase (EC 3.6.1.15) converting thiamine di-phosphate in thiamine phosphate, thiamine di-phosphokinase (EC 2.7.6.2) and thiamine phosphate phosphatase that lead the conversion of thiamine to thiamine di-phosphate and thiamine phosphate in thiamine, respectively (Fig. 3B).

**Figure 4:**
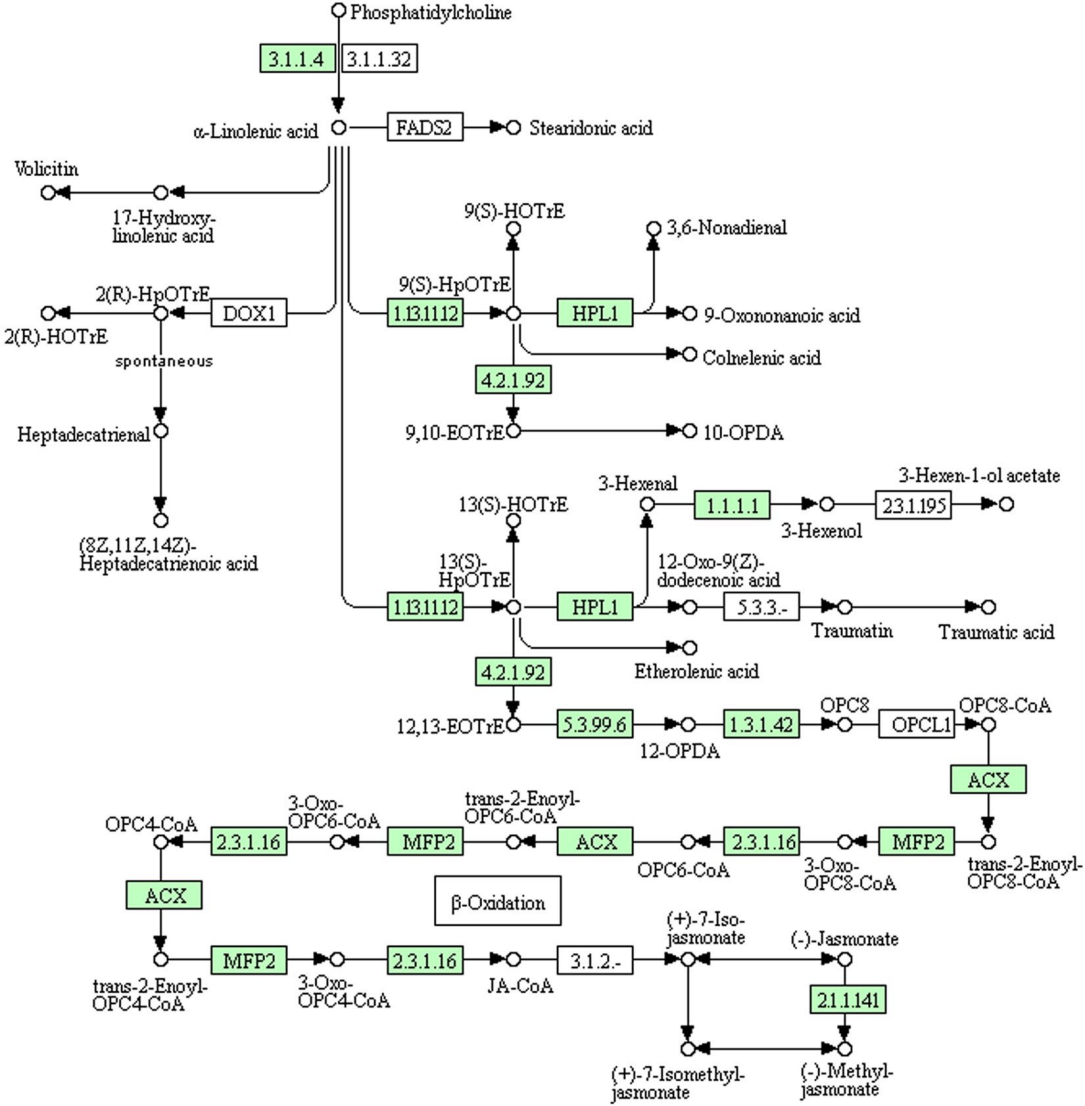
KEGG analysis showing genes involved in α-linolenic acid metabolism in *Capparis spinosa* leaf transcriptome (Enzyme Code - EC - identified are in green).

In the same way, α-linolenic acid metabolism (12 genes) is highly represented. In particular we identified jasmonate O-methyltransferase (EC 2.1.1.141) and acetyl-CoA C-acyltransferase (EC 2.3.1.16), involved in jasmonate biosynthesis (Fig. 4).

**Figure 5:**
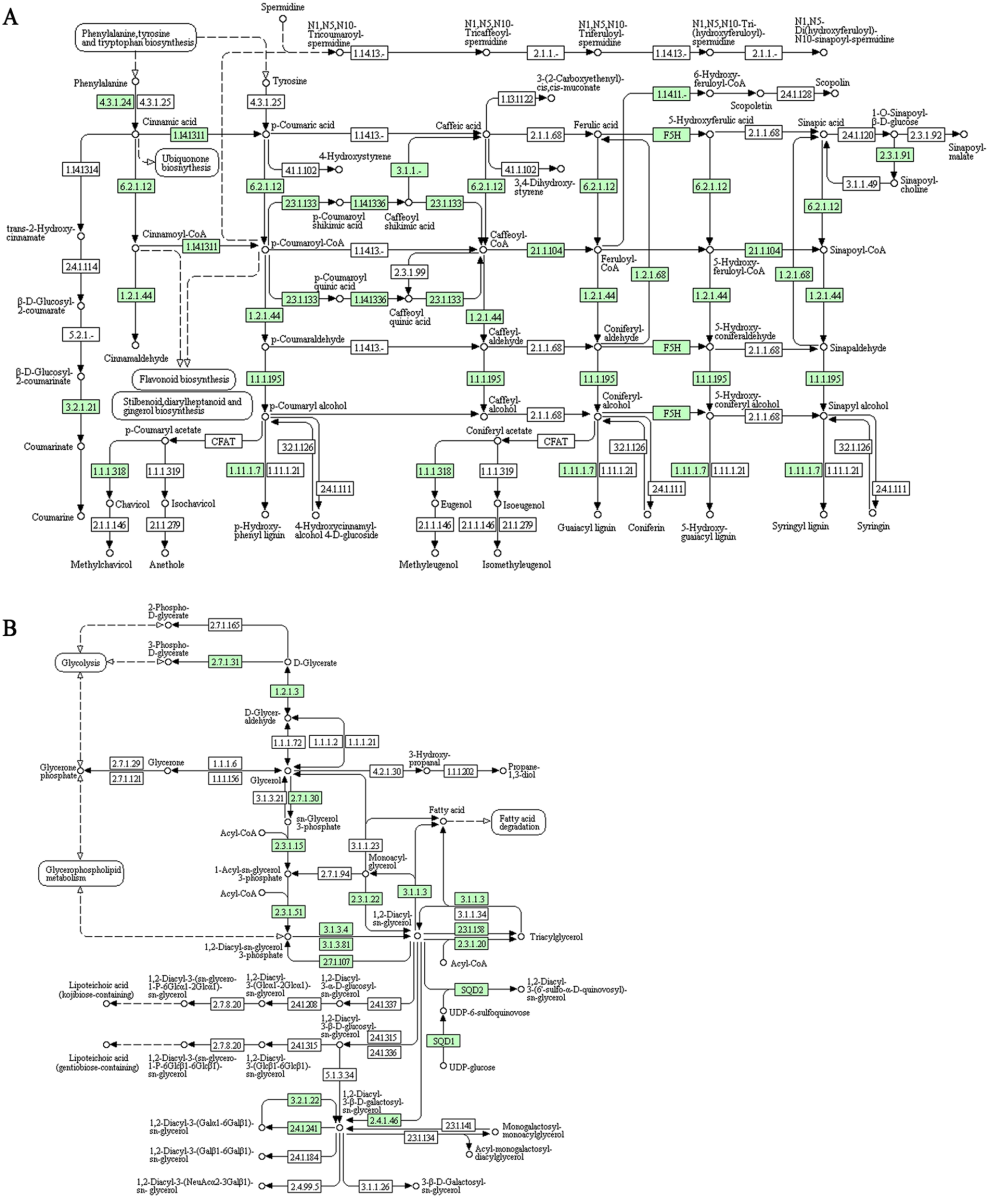
KEGG analysis showing genes involved in phenylpropanoid biosynthesis (**A**) and glycerolipid metabolism (**B**) in *Capparis spinosa* leaf transcriptome (Enzyme Code - EC - identified are in green).

A large proportion of phenylpropanoid biosynthesis pathway was also reconstructed (18 genes), identifying some important enzymes, such as phenylalanine ammonia lyase (PAL) (EC 4.3.1.24), the first component in the phenylpropanoid pathway; 4-coumarate-CoA ligase (EC 6.2.1.12) andcinnamate-4-hydroxylase (C4H) (EC 1.14.13.1), that convert trans-cinnamic acid (CA) to p-coumaric acid (COA); 4-coumarate CoA ligase involved in p-coumaroyl-CoAsynthesis, an intermediate for hydroxycinnamic acids, flavonols and flavonol derivatives (Fig. 5A).

Considering the role of lipids as signaling in plant responses to abiotic stress, the unitranscripts were investigated for coding sequences of lipid metabolism. In this highly represented pathway, we found key enzymes of glycerolipid metabolism involved in the phosphatidic acid (PA) synthesis, such as 1-acyl-sn-glycerol-3-phosphate acyltransferase (EC 2.3.1.51) and diacylglycerol kinase (ATP) (EC 2.7.1.107), converting lysophosphatidic acid and L-1, 2-diacylglycerol, respectively, in PA; and in PA transformation (phosphatidate phosphatase; EC 3.1.3.4) (Fig. 5B). We also identified CDP-diacylglycerol-inositol 3-phosphatidyltransferase (EC 2.7.8.11) that catalyzes phosphatidylinositol (PI) synthesis (Fig. 5B).

To complete the analysis of molecules playing important role during environmental stress response, the occurrence of components of sphingolipid metabolism was also explored and 10 different enzymes could be retrieved. A large proportion of sphingolipid metabolism pathways could be reconstructed, including, among others, alkaline ceramidase (EC 3.5.1.23) involved in the synthesis of sphingosine, and serine palmitoyltransferase (EC 2.3.1.50) a key enzyme of sphingolipid metabolism required for the conversion of L-serine and palmitoyl-CoA into 3-Dehydrosphinganine (Supplementary Fig. S2A).

Additionally, we identified sequences mapped in different pathways involved in phytochemical biosynthesis, such as terpenoid metabolism (36), carotenoids (12 genes), glucosinolates (9), stilbenoids (4), and anthocyanins (2). Genes involved in oxidative phosphorylation (60) and photosynthesis (28), such as cytochrome c oxidase (EC 1.9.3.1) and photosystem I/II, respectively, were also detected and well represented (Supplementary Fig. S2B,C).

Seven genes, *YODA* (mitogen-activated protein kinase kinase kinase), *ER* (*ERECTA)*, *EPLF9/STOMAGEN* (epidermal patterning factor-like protein 9), *TMM* (too many mouths) *ERL1* (erecta-like1), *GTL1* (*GT2-like1*) and *FAMA* (*FMA/bHLH097*), known to be involved in the modulation of stomatal development in response to drought, were also found (Supplementary Dataset S1). In addition, we identified transcripts with homology to Stress Associated Proteins (SAPs) genes that are potential candidates to improve abiotic stress tolerance in plants using biotechnological approaches^65^. We found 32 *C. spinosa* unitranscripts homologous to ten genes encoding for *A. thaliana* and *O. sativa* SAPs (Table 3; Supplementary Dataset S1). The average length of the transcripts is 1,607 bp, with values ranging between 249 bp (TRINITY_DN33098_c0_g1_i1; SAP10) and 4,447 bp (TRINITY_DN23049_c0_g1_i3; SAP1).

**Table 3:** List of *Capparis spinosa* leaf transcripts homologous to genes encoding for SAPs.

Gene code	Gene name	Species	Homology (%)*	*C. spinosa* L. transcripts
*SAP1*	*Stress-associated protein 1*	*A. thaliana*	69.23	TRINITY_DN23049_c0_g1_i2
			68.64	TRINITY_DN23049_c0_g3_i1
			69.23	TRINITY_DN23049_c0_g1_i3
			69.23	TRINITY_DN23049_c0_g1_i4
			68.64	TRINITY_DN23049_c0_g3_i3
			69.23	TRINITY_DN23049_c0_g1_i7
			69.23	TRINITY_DN23049_c0_g1_i8
*SAP2*	*Stress-associated protein 2*	*A. thaliana*	58.24	TRINITY_DN21298_c0_g1_i2
*SAP3*	*Stress-associated protein 3*	*A. thaliana*	65.03	TRINITY_DN23901_c0_g2_i2
			62.18	TRINITY_DN23049_c0_g2_i4
			62.18	TRINITY_DN23049_c0_g2_i12
			62.18	TRINITY_DN23049_c0_g2_i14
*SAP4*	*Stress-associated protein 4*	*A. thaliana*	58.58	TRINITY_DN20182_c1_g1_i1
			58.58	TRINITY_DN20182_c1_g1_i2
			58.58	TRINITY_DN20182_c1_g1_i3
			58.58	TRINITY_DN20182_c1_g1_i4
			87.5	TRINITY_DN26805_c2_g4_i1
			80.85	TRINITY_DN21640_c0_g3_i4
			80.85	TRINITY_DN21640_c0_g3_i6
			80.85	TRINITY_DN21640_c0_g3_i8
*SAP5*	*Stress-associated protein 5*	*A. thaliana*	57.87	TRINITY_DN18210_c0_g1_i2
			55.91	TRINITY_DN18210_c0_g2_i1
*SAP7*	*Stress-associated protein 7*	*A. thaliana*	66.48	TRINITY_DN23049_c0_g5_i1
*SAP8*	*Stress-associated protein 8*	*A. thaliana*	57.25	TRINITY_DN5273_c0_g1_i1
		*O. sativa*	60.92	TRINITY_DN21298_c0_g1_i1
		*O. sativa*	60.69	TRINITY_DN21298_c0_g1_i11
*SAP10*	*Stress-associated protein 10*	*A. thaliana*	60.32	TRINITY_DN33098_c0_g1_i1
*SAP11*	*Stress-associated protein 11*	*A. thaliana*	77.95	TRINITY_DN19581_c0_g1_i1
			79.86	TRINITY_DN19581_c0_g1_i2
*SAP12*	*Stress-associated protein 12*	*A. thaliana*	65.52	TRINITY_DN14592_c0_g1_i1
			70.76	TRINITY_DN14592_c0_g1_i2
			66.19	TRINITY_DN14592_c0_g1_i3

^*^The homology (best hit) was obtained blasting *C. spinosa* transcripts to NR database.

### Simple sequence repeats isolation and validation

A total of 5,009 perfect simple sequence repeats (SSR) with repeat numbers ranging from 4 to 31 (from di- to hexa- nucleotide motifs) were identified using the MISA tool in the assembled uniscripts (Supplementary Table S2). Trinucleotide repeats were the most abundant (2,756, 55.0%), followed by hexanucleotide (1,115, 22.3%), tetranucleotide (566, 11.3%), dinucleotide (362, 7.2%) and pentanucleotide (210, 4.2%) (Table 4). The most common repeat number was 7, observed in 1,146 assemblies (22.9%), followed by 8 (854, 17.1%), 4 (828, 16.5%), and >10 (794, 15.9%) tandem repeats (Table 4). The most abundant motifs detected were TCT (320, 6.4%) and TTC (303, 6.1%), followed by GAA (272, 5.4%). More details about different repeat motif for the isolated EST-SSRs are listed in Supplementary Table S2.

**Table 4:** Summary of EST-SSRs and their repeat motif isolated from *Capparis spinosa* leaf transcriptome.

Repeat motif	Number of repeat units								Total	(%)
	4	5	6	7	8	9	10	>10		
Di- nucleotide	—	—	—	—	—	—	—	362	*362*	*7.22*
Tri- nucleotide	—	—	—	1070	810	227	239	410	*2,756*	*55.02*
Tetra- nucleotide	—	343	152	17	15	19	2	18	*566*	*11.30*
Penta- nucleotide	—	131	48	19	8	4	—	—	210	*4.20*
Hexa- nucleotide	828	120	82	40	21	15	5	4	*1,115*	*22.26*
*Total*	*828*	*594*	*282*	*1,146*	*854*	*265*	*246*	*794*	*5,009*	
%	*1*6*.53*	*11.86*	*5.63*	*22.88*	*17.05*	*5.29*	*4.91*	*15.85*		

**Figure 6:**
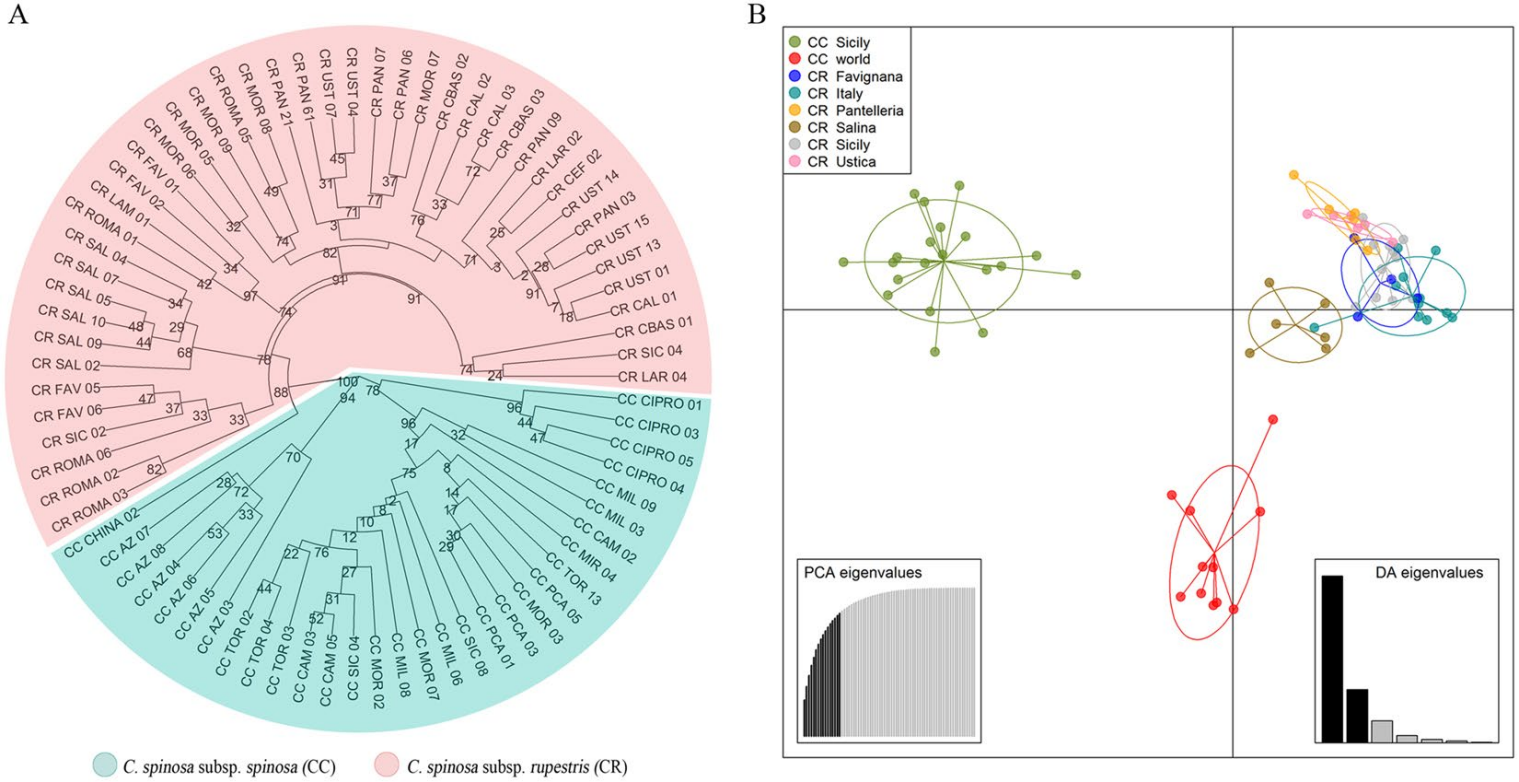
Genetic relationships among genotypes belonging to *Capparis spinosa* collection sampled across the distribution area of the species. (**A**) Dendrogram generated by 14 polymorphic EST-SSR developed in the present study, using the UPGMA method and Bruvo’s distance. (**B**) DAPC analysis clustering of the eight populations studied using the first two principal components (Y-axis and X-axis, respectively). CC: *C. spinosa* subsp. *spinosa*; CR: *C. spinosa* subsp. *rupestris*. The samples used for the EST-SSR validation were gathered in 8 main groups: CC Sicily, CC world, CR Favignana, CR Italy, CR Pantelleria, CR Salina, CR Sicily and CR Ustica.

Hundred-fifty primer pairs were designed using Primer3 (http://primer3.sourceforge.net/) and a first panel of 50 EST-SSRs was tested (Supplementary Table S3). The predicted SSRs were validated and evaluated for the polymorphism rate by using a set of 75 *C. spinosa* genotypes, collected across the distribution area of the species (Supplementary Table S4). Forty-one out of 50 tested EST-SSRs showed amplified fragments. Among them, 14 fragments fell outside the expected size range, and were not considered further. The other 27 EST-SSRs produced PCR fragments with the expected size, 14 of which were polymorphic with a number of alleles per locus ranging from 2 to 11 (mean 6), and values of He from 0.420 to 0.843 (mean 0.630) (Table 5), PIC from 0.332 to 0.826 (mean 0.583), Fis and Fst values from −0.058 to 0.830 (mean 0.062) and from 0.010 to 0.695 (mean 0.495), respectively (Table 5). The selected EST-SSRs showed strong discrimination power among the different taxa here considered. UPGMA phylogenetic tree and DAPC analysis based on SSR clearly discriminated the thorny group of *C. spinosa* subsp. *spinosa* from Italy, Creta and west Asia from the thornless group of *C. spinosa* subsp. *rupestris* from different regions and islands of Italy (hereinafter subsp. *spinosa* and *rupestris*, respectively) (Fig. 6). Among the group of subsp. *spinosa*, Sicilian populations differentiated from eastern Mediterranean and western Asia populations. The subsp. *rupestris* was more homogeneous, though samples from the small islands of Salina and, to a minor extent, Pantelleria and Ustica also skewed from the rest of the populations (Fig. 6).

**Table 5:** Main genetic parameters from the 14 polymorphic EST-SSR loci of the population under investigation (sample size 75).

Marker	Allele	Size range	He	Ho	PIC	Fis	Fst
ESTcapp5	11	95–125	0.750	0.587	0.721	−0.003	0.495
ESTcapp8	6	122–137	0.700	0.118	0.647	0.830	0.515
ESTcapp10	5	134–142	0.529	0.187	0.498	0.602	0.279
ESTcapp11	2	147–153	0.420	0.440	0.332	−0.057	0.010
ESTcapp14	4	136–148	0.531	0.107	0.476	0.768	0.382
ESTcapp18	5	102–112	0.462	0.440	0.441	−0.055	0.210
ESTcapp20	5	120–128	0.631	0.254	0.561	0.471	0.695
ESTcapp21	6	130–145	0.585	0.480	0.520	−0.026	0.325
ESTcapp32	10	159–181	0.735	0.227	0.699	0.519	0.657
ESTcapp33	9	136–163	0.843	0.548	0.826	0.245	0.594
ESTcapp35	4	101–113	0.472	0.480	0.417	−0.058	0.096
ESTcapp37	6	86–106	0.759	0.533	0.718	0.062	0.494
ESTcapp46	6	129–156	0.687	0.347	0.636	0.310	0.574
ESTcapp49	8	131–161	0.711	0.440	0.677	−0.014	0.610
*Mean*	*6*		*0.630*	*0.370*	*0.583*	*0.257*	*0.424*

He: Genetic diversity; Ho: Observed heterozygosity; PIC: Polymorphism Information Content; Fis: Inbreeding coefficient; Fst: Fixation index.

## Discussion

Although *C. spinosa* is a rich source of bioactive compounds with important nutritional and medicinal values^18,25,66–68^, until now available genomics resources were limited. The lack of adequate molecular markers and genes identification are a limit for an efficient employment of this orphan crop, displaying agro-based potentialities and a high demand for exploitation^7^. In addition, the natural resistance to drought and harsh environmental conditions makes *C. spinosa* a potentially important resource in areas threatened by global warming and desertification. Therefore, the transcriptomic data generated in this study provide useful resources to support a full taxonomic revision of the genus *Capparis* (Capparaceae), and assist selection in the modern breeding programs in order to promote this crop, especially in the Mediterranean countries.

Illumina next-generation RNA-Seq was successfully used to develop a high-quality leaf transcriptome of *C. spinosa* subsp. *rupestris*, generating a number of transcripts, similar to other transcriptome studies on plants^54,69–71^. Although our work is focused only on one vegetative tissue (leaf), the first transcriptome profile of *C. spinosa* grown under natural conditions has been developed. About 72% of the unitranscripts were successfully assigned to genes in the NR database, and likewise a large number of unigenes (68%) and predicted proteins (50%) showed match by querying against UniRef90 database. In addition, species distribution analysis showed a high homology with *Brassicaceae*, a sister family to *Cleomaceae*, underlining the close evolutionary relationship of *C. spinosa* with this family^72^.

Biological pathways identification plays a crucial role to shed light into functional analysis and transcriptomic data. KEGG is an integrated database resource that integrates genomic, chemical and systemic functional information, a useful tool for the interpretation of transcriptomic data and widespread interrogation of an organism’s genome content^73^. Here, a number of pathways of *C. spinosa* were highly represented. Among these, we described in detail those involved in abiotic stress tolerance and bio-compounds production. We studied purine and thiamine metabolism, α-linolenic acid metabolism, phenylpropanoid biosynthesis, lipid metabolism, genes involved in stomatal development and distribution, and lastly the presence of SAPs.

Purine metabolism, particularly thiamine (vitamin B1) and related phosphate esters are involved as cofactors in response to abiotic and biotic stress^74^. Thiamine metabolism can be altered under environmental stress in *Zea mays*^75^, while in *A. thaliana* the abiotic defenses activation and stress tolerance were triggered by altered adenine metabolism^61^. Cellular adenine levels drive plant growth and biomass increase, playing a key role as signal in the response modulation to abiotic stress and acclimation^61^. Moreover, a recent study^76^ suggested a possible connection between purine catabolism and stress phyto-hormone homeostasis/signaling. Takagi *et al*.^76^ showed how allantoin, a metabolic intermediate of purine catabolism accumulates in plants under abiotic stress, activating the jasmonic acid responses via abscisic acid (ABA) and enhancing seedling tolerance to abiotic stress.

Several putative targets involved in plant abiotic stress response, belonging to α-linolenic acid metabolism, phenylpropanoid biosynthesis and lipid metabolism, were also found in this study. Comparing transcriptomic profiles of susceptible and tolerance rice varieties, α-linolenic acid metabolic pathway appears involved in the high drought tolerance^77^ and, recently, a link between α-linolenic acid and jasmonic acid biosynthesis with cold acclimation was uncovered in *Camellia japonic*a through RNA-Seq analysis^63^. Phenylpropanoid pathway is responsible for the synthesis of a wide range of secondary metabolites in plant. As expected, the analysis revealed that the majority of the metabolic genes of this pathway are expressed in *C. spinosa* leaves. In particular we identified *PAL* and *C4H*, genes encoding enzymes that catalyze the first and second step of phenylpropanoid way, respectively, and responsible for biosynthesis of lignin. *C4Hs* have remained highly conserved across the plant kingdom and recent studies^78,79^ highlighted their key role in response to stresses (drought and cold) and as scavengers of Reactive Oxygen Species (ROS). In addition, genes linked to stress responses, including ethylene biosynthesis and signaling, showed altered expression levels in PAL knocked-down plants under non-challenging conditions^80^. PAL is also a biosynthetic source of salicylic acid (SA) in plants^81^, a master regulator in biotic and abiotic stress response in plants, including drought stress^82,83^.

Key enzymes of glycerolipid metabolism driving the PA synthesis were also detected. PA is a diacyl glycerophospholipid used as precursor for complex lipids biosynthesis and transiently generated in response to biotic and abiotic stress in plants. PA plays an essential role in ABA-induced production of ROS, osmotic changes and temperature stress response^84–87^. In the same way, since lipid-protein interactions are crucial for deciphering the signaling cascades, we studied and isolated phosphoinositides and sphingolipids, compounds belonging to the highly coordinated signaling network developed in plants, linked to acclimation or survival under abiotic stress^88^.

*C. spinosa* is drought tolerant and shows an efficient hydraulic conductivity due to the well-developed xylem vessels in stems^7,89^. Therefore, based on these evidences, we further assessed the presence of leaf transcripts homologous to genes involved in stomatal development and distribution that can be considered as key genes in the response to drought stress and water use efficiency (WUE). We found seven transcripts related to stomata. In particular, *YODA* is a MAPKK kinase gene and *GTL1*a transcriptional repressor of *SDD1*, a negative regulator of stomata development^90^ and density^91^. *ERECTA* has been the first identified major effector of WUE and a recent study^92^ demonstrated that the *EDT1/HDG11-ERECTA-E2Fa* genetic pathway reduced the stomatal density by increasing cell size, providing a new strategy to improve WUE in crops. The presence of *YODA*, *ERECTA* and *GTL1* in the assembled unitranscripts might be associated to an adaptive response of *C. spinosa* to drought.

We also focused our attention to the presence of homologs encoding for SAPs. These A20/AN1 zinc-finger proteins have been shown to confer tolerance to multiple abiotic stresses in plants. In *A. thaliana*, *AtSAP9* regulates abiotic/biotic stress responses probably via the ubiquitination/proteasome pathway^93^ and *AtSAP13* is upregulated in response to Cd, ABA, and salt stresses^94^. In rice, SAP homologs are activated by multiple abiotic stresses (such as cold, salt, and dehydration)^95^. In *Prunus*, water retention and cell growth are regulated by a stress-associated protein (*PpSAP1*) through the *TARGET OF RAPAMYCIN* (TOR) pathway^96^. In poplar, the downregulation of *PagSAP1* increases salt stress tolerance^97^. The finding of SAP homologs highlights the possible mechanisms involved in the adaptability of *C. spinosa* to a wide range of environmental conditions.

The production of secondary metabolites with medicinal properties could be reflected by the presence in our transcriptome of several genes involved in phytochemicals biosynthesis: prolycopene isomerase (EC 5.2.1.13) belonging to carotenoids and involved in lycopene biosynthesis; farnesyl diphosphate synthase (FPS) (EC 2.5.1.10), that catalyzes the synthesis of farnesyl diphosphate (FPP) in terpenoid metabolism; enzymes involved in chlorogenic acids (CGA) production, an important scavenging and antioxidant compound^98^; the anthocyanidin 3-O-glucoside 5-O-glucosyltransferase (EC 2.4.1.298) that converts pelargonidin 3-glucoside in pelargonin, compound with antioxidant activity; enzymes involved in glucobrassicin and glucoiberverin biosynthesis (glucosinolates) known as anti-cancer agents^99^; the MYB transcription factor *Rosea1* (*Ros1*) that, together with *Delila*, enhances anthocyanin accumulation and abiotic stress tolerance in tobacco^100^.

Finally, we developed the first panel of co-dominant markers (EST-SSR) in caper. So far, genetic analysis of *Capparis* germplasm has largely relied on AFLP, RAPD, and ISSR markers^34,101,102^. The main reasons for using dominant markers were the lack of a genome sequence and/or transcriptome information for this species. Here, we identified 5,009 microsatellites from the assembled transcriptome in agreement with the frequencies reported in other studies^52,103–107^. When mono-nucleotide repeats were excluded, tri-nucleotide repeats were the most abundant class of SSRs, with TCT the most frequent motif in our dataset. This finding is consistent with results reported in other species, such as rice, wheat, barley^107,108^, cotton^109^ and asparagus^110^. To determine the level of polymorphism and discrimination power among this first set (50) of EST-SSRs, the markers were tested and validated using 75 selected samples. Nine primers pairs failed to produce amplicons, possibly due to primers spanning splicing sites, large introns, chimeric primer(s), or poor-quality sequences introns^106,111^. Fourteen primer pairs produced amplicons that deviated from expected size, which might have been produced by the presence of introns^106,111^, large insertions or repeat number variations, or a lack of specificity. Conversely, 27 EST-SSRs were validated, 14 of which (52%) were polymorphic. The polymorphism level was higher than the values reported in previous studies^34,101,102^, with genetic diversity values (H_e_) > 0.5 for 11 out of 14 polymorphic EST-SSRs. These results suggest that the isolated sequences are suitable for the development of specific primers and confirmed the quality of the transcriptome assembled. Moreover, cluster and DAPC analysis highlighted the ability of selected EST-SSR to discriminate among taxa and origin of *C. spinosa* samples here analysed. In particular, for the subsp. *spinosa* the Sicilian plants grouped together and were separated by the rest of Mediterranean and Asian samples. In this regard, it is noteworthy that the plants from the Mediterranean island of Cyprus grouped together with Asian plants from Azerbaijan and China, rather than with Sicily. It is therefore tempting to speculate that a more extensive analysis of molecular polymorphism with samples collected worldwide with the SSR developed here might reveal unexpected scenarios of diffusion and evolution of caper. For the group of subsp. *rupestris*, the germplasm available was limited to Southern Italy, mainland Sicily and its minor islands (Supplementary Table S4). Consequently, due to the proximity of the sampling sites, the germplasm was more homogeneous respect to what observed for the subsp. *spinosa* (Fig. 6). Nevertheless, samples collected in the minor islands of Salina clearly formed a separate group. Caper plants from the islands of Pantelleria and Ustica also skewed from mainland Sicily and Italy, though less so. The minor island of Favignana, on the other hand, did not outgroup. Interestingly, caper cultivation is a major agricultural activity in the islands of Salina, Pantelleria and Ustica, but not in Favignana and in the rest of Sicily. It is therefore possible that attempts of selections by local farmers caused the observed deviation compared to wild type populations. Moreover, farmers in Salina traditionally propagate caper plants by clonal cuttings, as opposed to farmers in Pantelleria and Ustica who usually employ seeds^112^. This difference might justify the pronounced separation in the samples from Salina, since individual diversity in the selected plants is faithfully preserved and the cultivated germplasm does not mix any longer with the wild ancestors.

The novel molecular markers developed can be used in future studies assessing genetic diversity, phylogenetic analysis, marker assisted selection (MAS), mapping and association analysis in *Capparis* species worldwide. In addition, the newly designed EST-SSR primers for *C. spinosa* can also be tested in other species of the Capparaceae family currently lacking of their own genomic resources.

## Methods

### Plant material

We selected for RNA-Seq analysis three wild populations of *C. spinosa* subsp. *rupestris* to maximize the variety of genetic backgrounds and environmental conditions, in order to enrich the trancriptomic information (Supplementary Table S5). The three populations were used as biological replicates. For each population, mature leaves from three different specimens were collected at the same vegetative stage and packed *in situ*, then immediately frozen in liquid nitrogen and stored in the laboratory at −80 °C until use. Furthermore, a panel of 75 wild *C. spinosa* samples (Supplementary Table S4) were collected across the natural distribution area of the species for DNA extraction in order to validate the EST-SSR markers isolated.

### RNA isolation and sequencing

RNA from each collected sample was extracted using a NucleoSpin RNA Plant (Macherey-Nagel GmbH & Co. KG, 52355 Düren, Germany) and treated with RNase-free DNase. RNA quality (RNA Integrity Number (RIN) > 8.0) was evaluated using an Agilent Bioanalyzer RNA nanochip (Agilent, Wilmington, DE). RNA-Seq libraries were independently prepared for three pools representing the three different populations. Each pool was composed by equal leaf amount of three specimens. Sequencing library was prepared using the Illumina TruSeq RNA Sample Preparation Kit v2 (Illumina, San Diego, CA, USA) according to manufacturer’s specifications; quality and insert size distribution were evaluated using Agilent Bioanalyzer DNA 1000 chip. Sequencing libraries were quantified using qPCR and sequenced in the same lane on an Illumina HiSeq. 1000 generating 2 × 100 nt paired-end reads.

### De novo assembly and functional annotation of leaf transcriptome

Raw reads were adapter clipped and quality trimmed following recommendations from previous studies^113,114^. Adapter sequence contamination and low quality nucleotides (PHRED < 5) were removed using Trimmomatic version 0.33^115^. *De novo* transcriptome assembly of cleaned reads was carried out in Trinity (v.2.5.1)^116^ with default parameters. To generate transcriptome containing only unique transcripts for downstream analysis, CD-HIT-EST^117^ was used (identity cut-off ≥ 90%) by removing all repetitive, identical and near-identical transcripts. The quality and completeness of the *de novo* assembly were evaluated using BUSCO3 software v3^118^. This quality assessment tool provides high-resolution quantifications for genomes, gene sets, and transcriptomes and checks whether each of the BUSCO group is complete, duplicated, fragmented, or missing in the genome or transcriptome assembly. The unitranscripts were compared to the set of Eudicotyledons genes, which contains 2121 BUSCO groups from a total of 40 species in order to obtain a quantitative measure of the transcriptome completeness, based on evolutionarily informed expectations of gene content from near-universal single-copy orthologs. In addition, to evaluate potential contamination about bacteria and endophytes, QUAST software^119^ was used.

Functional annotation of unitranscripts was performed using the Trinotate pipeline (http://trinotate.sourceforge.net/) to identify open reading frames and assign best hits to UniprotKB (1*10^−5^), UniRef90 (1*10^−5^), PFAM-A (1*10^−5^), GO and KOG categories^120^. Transdecoder (v.3.1.0) (https://transdecoder.github.io/) was also used to *de novo* predict putative coding regions and protein sequences. Blastp search was carried out by using predicted Open Reading Frames (ORFs) as the query and the Swiss-Prot non-redundant database as the target. The HMMER package^121^ and Pfam databases^122^ were utilized to predict protein domains, while SignalP 4.1^123^ was used to predict the presence of signal peptides within the predicted ORFs. CateGOrizer^124^ was used to map GO terms to a parent plant to get a wide overview of the transcripts functional classification. Finally, KEGG Automatic Annotation Server (KAAS, http://www.genome.jp/kaas-bin/kaas_main?mode=est) was employed to map KEGG pathways of assigned caper orthologs^125–128^. All figures showing identified and highlighted pathways were developed through KEGG Mapper, Search Pathway using unique Kos (https://www.genome.jp/kegg/tool/map_pathway1.html). KO assignments were performed based on the bi-directional best hit of BLAST^129^.

### Identification and validation of polymorphic EST-SSR markers

All clustered transcripts generated from *de novo* assembly were examined to identify new co-dominant molecular markers. SSRs based on short tandem repeats were identified through analyses of ESTs (EST–SSR) and carried out into gene-anchored marker loci^130^. SSR loci were detected using MicroSAtellite tool (MISA; http://pgrc.ipk-gatersleben.de/misa/misa.html)^131^. Di-, tri-, tetra-, penta-, and hexa-nucleotides were searched with a minimum of 20, 7, 5, 5, and 4 repeat units, respectively. A set of primer pairs (150) was designed using Primer3 software (http://primer3.sourceforge.net/)^132^ by imposing an amplicon size range of 100–400 bp, minimum and maximum GC contents 40 and 60%, and minimum and maximum melting temperature (*Tm*) values ranging from 58 to 60 °C, respectively (Supplementary Table S3). A first panel of 50 EST-SSR was tested and validated, verifying the primers specificity and amplicons size (Supplementary Table S3) on the above-mentioned panel of samples (Supplementary Table S4). Genomic DNA was extracted from leaves (200 mg) using the CTAB protocol^133^. DNA concentration and quality were checked with a Nanodrop ND1000 (Thermo Scientific). PCR amplifications were carried out in 20 µl reaction mixtures starting from 50 ng of DNA as previously described^110^. The fragments were analyzed on an ABI PRISM 3500 Genetic Analyzer (Applied Biosystems) and the alleles were sized by GENEMAPPER 4.0 (Applied Biosystems). Genetic diversity (He), mean allele number, fixation index (Fst), inbreeding coefficient (Fis) and Polymorphism Information Content (PIC) for each EST-SSR used were calculated by using PowerMarker v. 3.25^134^ and R/poppr^135^. Genetic relationships among studied genotypes were also investigated by cluster analysis and Discriminant Analysis of Principal Components (DAPC). The UPGMA (Unweighted Pair Group Method with Arithmetic Mean) phylogenetic tree was designed by using R/poppr^135^ with Bruvo’s distance^136^. The bootstrap analysis was performed based on 1,000 resamplings. DAPC, implemented in the R/adegenet^137^, was performed to infer population subdivision of the analysed collection, regardless of the geographic origin. Since only one sample (LAM01) belonging to the Lampedusa island population was available, this population has been excluded from DAPC analysis. In the output, samples were gathered in 8 main groups (Fig. 6B; Table S6). The number of principal components (PCs) retained was evaluated using the cross-validation procedure. We also used the K-means algorithm, ‘find.clusters’, to independently verify the assignment of individuals to clusters.

## Supplementary information

S1

S2

Supplementary information

## Data Availability

RNA-Seq generated for this project has been deposited in the NCBI BioProject (PRJNA311285) and NCBI SRA (SAMN04481463). The Transcriptome Shotgun Assembly project (submission number SUB5676995) has been deposited at DDBJ/EMBL/GenBank under the accession GHNF00000000. The version described in this paper is the first version, GHNF01000000. The assembled transcriptome can be accessed via NCBI (https://www.ncbi.nlm.nih.gov/genbank/tsa/). The microsatellite data for the population samples of *C. spinosa* are available in this article.

## References

[1] Yamori W, Hikosaka K, Way DA (2014). Temperature response of photosynthesis in C3, C4, and CAM plants: temperature acclimation and temperature adaptation. Photosynth. Res..

[2] Xu Z, Jiang Y, Zhou G (2015). Response and adaptation of photosynthesis, respiration, and antioxidant systems to elevated CO2 with environmental stress in plants. Front. Plant Sci..

[3] Bita CE, Gerats T (2013). Plant tolerance to high temperature in a changing environment: scientific fundamentals and production of heat stress-tolerant crops. Front. Plant Sci..

[4] Ray DK, Gerber JS, MacDonald GK, West PC (2015). Climate variation explains a third of global crop yield variability. Nat. Commun..

[5] Pachauri, R. K. *et al*. “Climate change 2014: synthesis report,” *in Contribution of Working Groups I, II and III to the Fifth Assessment Report of the Intergovernmental Panel on Climate Change*, eds Pachauri, R. and Meyer, L. (Geneva: IPCC), 151 (2014).

[6] Thiry AA, Dulanto PNC, Reynolds MP, Davies WJ (2016). How can we improve crop genotypes to increase stress resilience and productivity in a future climate? A new crop screening method based on productivity and resistance to abiotic stress. J. Exp. Bot..

[7] Chedraoui S (2017). *Capparis spinosa* L. in A Systematic Review: A xerophilous species of multi values and promising potentialities for agrosystems under the threat of global warming. Front. Plant Sci..

[8] Jacobs M (1965). The genus *Capparis* (Capparaceae) from the Indus to the Pacific. Blumea.

[9] Maire, R. Flore de l’Afrique du Nord. Paris: 12 Editions Paul Lechevalier (1965).

[10] St. John HS (1965). Revision of *Capparis spinosa* and its African, Asiatic and Pacific relatives. Micronesica.

[11] Higton RN, Akeroyd JR (1991). Variation in *Capparis spinosa* L. in Europe. Bot. J. Linn. Soc..

[12] Heywood, V. H. “*Capparis* L.” in *Flora Europaea*, vol 1, ed. Cambridge University Press (Cambridge) 312 (1993).

[13] Fici S (2001). Intraspecific variation and evolutionary trends in *Capparis spinosa* L. (Capparaceae). Plant Syst. Evol..

[14] Zohary M (1960). The species of *Capparis* in the Mediterranean and the Near Eastern countries. B. Res. Coun. Israel.

[15] Inocencio C, Rivera D, Obón C, Alcaraz F, Barrena J (2006). A systematic revision of *Capparis* section *Capparis* (Capparaceae). Ann. Mo. Bot. Gard..

[16] Danin A (2010). *Capparis* in the East Mediterranean countries. Fl. Medit..

[17] Fici S (2014). A taxonomic revision of the *Capparis spinosa* group (Capparaceae) from the Mediterranean to Central. Asia. Phytotaxa.

[18] Rivera D, Inocencio C, Obón MC, Alcaraz F (2003). Review of food and medicinal uses of *Capparis* L. subgenus *Capparis* (Capparidaceae). Econom. Bot..

[19] Eddouks M, Lemhardi A, Michel JB (2005). Hypolipidemic activity of aqueous extract of *Capparis spinosa* L. in normal and diabetic rats. J. Ethnopharmacol..

[20] Lemhadri A, Eddouks M, Sulpice T, Burcelin R (2007). Anti-hyperglycaemic and anti-obesity effects of *Capparis spinosa* and *Chamaemelum nobile* aqueous extracts in HFD Mice. Am. J. Pharm. Toxicol..

[21] Tesoriere L, Butera D, Gentile C, Livrea MA (2007). Bioactive components of Caper (*Capparis spinosa* L.) from Sicily and antioxidant effects in a red meat simulated gastric digestion. J. Agr. Food. Chem..

[22] Sze-Kwan L, Tzi-Bun N (2009). A protein with antiproliferative, antifungal and HIV-1 reverse transcriptase inhibitory activities from caper (*Capparis spinosa*) seeds. Phytomedicine.

[23] Haifeng Z (2010). Anti inflammatory effects of caper (*Capparis spinosa* L.) fruit aqueous extract and the isolation of main phytochemicals. J. Agric. Food Chem..

[24] Abraham SVPI, Palani A, Ramaswamy BR, Shunmugiah KP, Arumugam VR (2011). Antiquorum sensing and antibiofilm potential of *Capparis spinosa*. Arch. Med. Res..

[25] Tlili N (2011). The caper (*Capparis* L.): ethnopharmacology, phytochemical and pharmacological properties. Fitoter..

[26] Huseini FH (2013). *Capparis spinosa* L. (Caper) fruit extract in treatment of type 2 diabetic patients: a randomized double-blind placebo-controlled clinical trial. Complement. Ther. Med..

[27] Hong-Juan L, Tao Y, Xue-Mei C, Chang-Hong W (2014). Comparative evaluation of anti-inflammatory and analgesic activities of various medicinal parts of *Capparis spinosa*: a consideration of ecological environment and resource conservation. Indian J. Med. Res. Pharm. Sci..

[28] Yu L (2017). Antioxidant and antitumor activities of *Capparis spinosa* L. and the related mechanisms. Oncol. Rep..

[29] Mamoci E (2011). Chemical composition and *in vitro* activity of plant extracts from Ferula communis and Dittrichia viscosa against postharvest fungi. Molecules.

[30] Araniti F, Lupini A, Mercati F, Statti GA, Abenavoli MR (2013). Calamintha nepeta L. (Savi) as source of phytotoxic compounds: bio-guided fractionation in identifying biological active molecules. Acta Physiol. Plant..

[31] Araniti F (2014). Phytotoxic activity of Cachrys pungens Jan, a Mediterranean species: separation, identification and quantification of potential allelochemicals. Acta Physiol. Plant..

[32] Caboni P (2012). Nematicidal Activity of 2-Thiophenecarboxaldehyde and Methylisothiocyanate from Caper (*Capparis spinosa*) against *Meloidogyne incognita*. J. Agric. Food Chem..

[33] Ladhari A, Omezzine F, Dellagreca M, Zarrelli A, Haouala R (2013). Phytotoxic activity of *Capparis spinosa* L. and its discovered active compounds. Allelopathy J..

[34] Gristina AS (2014). Hybridization in *Capparis spinosa* L.: Molecular and morphological evidence from a Mediterranean island complex. Flora.

[35] Hall JC (2008). Systematics of Capparaceae and Cleomaceae: an evaluation of the generic delimitations of *Capparis* and *Cleome* using plastid DNA sequence data. Botany.

[36] Siragusa M, Carimi F (2009). Development of specific primers for cpSSR analysis in caper, olive and grapevine using consensus chloroplast primer pairs. Scie. Hortic..

[37] Wang Q, Zhang ML, Yin LK (2016). Phylogeographic structure of a tethyan relict *Capparis spinosa* (Capparaceae) traces Pleistocene geologic and climatic changes in the western Himalayas, Tianshan mountains, and adjacent desert regions. Biomed. Res. Int..

[38] Grewe F (2014). Comparative analysis of 11 Brassicales mitochondrial genomes and the mitochondrial transcriptome of *Brassica oleracea*. Mitochondrion.

[39] Jiao Y (2012). Development of simple sequence repeat (SSR) markers from a genome survey of Chinese bayberry (*Myrica rubra*). BMC Genomics.

[40] Carimi F (2011). Intra-varietal genetic diversity of the grapevine (*Vitis vinifera* L.) cultivar ‘Nero d’Avola’ as revealed by microsatellite markers. Genet. Res. Crop. Evol..

[41] Mercati F (2015). Genetic variation of an Italian long shelf-life tomato (*Solanum lycopersicon* L.) collection by using SSR and morphological fruit traits. Genet. Res. Crop. Evol..

[42] Fu Y (2017). Patterns of SSR variation in bread wheat (*Triticum aestivum* L.) seeds under *ex situ* gene-bank storage and accelerated ageing. Genet. Res. Crop. Evol..

[43] Gristina AS (2017). Urgent need for preservation of grapevine (*Vitis vinifera* L. subsp. *vinifera*) germplasm from small circum-Sicilian islands as revealed by SSR markers and traditional use investigations. Genet. Res. Crop. Evol..

[44] Morgante M, Olivieri AM (1993). PCR-amplified microsatellites as markers in plant genetics. Plant J..

[45] Zhao Y, Williams R, Prakash CS, He G (2013). Identification and characterization of gene-based SSR markers in date palm (*Phoenix dactylifera* L.). BMC Plant Biol..

[46] Li YC, Korol AB, Fahima T, Nevo E (2004). Microsatellites within genes: structure, function, and evolution. Mol. Biol. Evol..

[47] Parchman TL, Geist KS, Grahnen JA, Benkman CW, Buerkle CA (2010). Transcriptome sequencing in an ecologically important tree species: assembly, annotation, and marker discovery. BMC Genomics.

[48] Ekblom R, Galindo J (2011). Applications of next generation sequencing in molecular ecology of non-model organisms. Heredity.

[49] Harkess A (2017). The asparagus genome sheds light on the origin and evolution of a young Y chromosome. Nat. Commun..

[50] Wang Z, Gerstein M, Snyder M (2009). RNA-Seq: a revolutionary tool for transcriptomics. Nat. Rev. Genet..

[51] Feng C (2012). Transcriptomic analysis of Chinese bayberry (*Myrica rubra*) fruit development and ripening using RNA-Seq. BMC Genomics.

[52] Wei L (2014). Transcriptome analysis of *Houttuynia cordata* Thunb. by Illumina Paired-End RNA Sequencing and SSR marker discovery. PLoS One.

[53] Xia Z (2011). RNA-Seq analysis and *de novo* transcriptome assembly of *Hevea brasiliensis*. Plant Mol. Biol..

[54] Harkess A (2015). J. Sex-biased gene expression in dioecious garden asparagus (*Asparagus officinalis*). New Phytol..

[55] Evangelistella C (2017). De novo assembly, functional annotation, and analysis of the giant reed (*Arundo donax* L.) leaf transcriptome provide tools for the development of a biofuel feedstock. BMC Biotechnol. Biofuels.

[56] Brautigam A, Mullick T, Schliesky S, Weber APM (2011). Critical assessment of assembly strategies for non-model species mRNA-Seq data and application of next-generation sequencing to the comparison of C_3_ and C_4_ species. J. Exp. Bot..

[57] Martin LB, Fei Z, Giovannoni JJ, Rose JK (2013). Catalyzing plant science research with RNA-seq. Front. Plant Sci..

[58] Carra A (2012). *In vitro* plant regeneration of caper (*Capparis spinosa* L.) from floral explants and genetic stability of regenerants. Plant Cell Tiss. Org. Cult..

[59] Singh R, Ming R, Yu Q (2016). Comparative analysis of GC content variations in plant genomes. Tropical Plant Biol..

[60] Tunc-Ozdemir M (2009). Thiamin confers enhanced tolerance to oxidative stress in Arabidopsis. Plant Physiol..

[61] Sukrong S (2012). Improved growth and stress tolerance in the Arabidopsis oxt1 mutant triggered by altered adenine metabolism. Mol. Plant.

[62] Wasternack C (2014). Action of jasmonates in plant stress responses and development - Applied aspects. Biotechnol. Adv..

[63] Li Q (2016). RNA-seq based transcriptomic analysis uncovers α-linolenic acid and jasmonic acid biosynthesis pathways respond to cold acclimation in *Camellia japonica*. Sci. Rep..

[64] Martinez V (2016). Accumulation of flavonols over hydroxycinnamic acids favors oxidative damage protection protection under abiotic stress. Front. Plant Sci..

[65] Giri J (2013). SAPs as novel regulators of abiotic stress response in plants. BioEssays.

[66] Sozzi, G. O. & Vicente, A. Capers and caperberries. In: Handbook of Herbs and Spices. Ed. Peter, K. V. (Cambridge, Woodhead Publishing Limited, CRC Press) **3**, 230–256 (2006).

[67] Gull T, Anwar F, Sultana B, Alcayde CAM, Nouman W (2015). *Capparis* species: a potential source of bioactives and high-value components: a review. Ind. Crops Prod..

[68] Nabavi SF (2016). Pharmacological effects of *Capparis spinosa* L. Phytother. Res..

[69] Yazawa T, Kawahigashi H, Matsumoto T, Mizuno H (2013). Simultaneous transcriptome analysis of *Sorghum* and *Bipolaris sorghicola* by using RNA-seq in combination with de novo transcriptome assembly. PLoS One.

[70] Han S (2017). Differential gene expression in leaf tissues between mutant and wild-type genotypes response to late leaf spot in peanut (*Arachis hypogaea* L.). PLoS One.

[71] Ponniah SK, Thimmapuram J, Bhide K, Kalavacharla V, Manoharan M (2017). Comparative analysis of the root transcriptomes of cultivated sweetpotato (*Ipomoea batatas* [L.] Lam) and its wild ancestor (*Ipomoea trifida* [Kunth] G. Don). BMC Plant Biol..

[72] Iltis HH, Hall JC, Cochrane TS, Sytsma KJ (2011). Studies in the Cleomaceae I. On the separate recognition of Capparaceae, Cleomaceae, and Brassicaceae. Ann. Mo. Bot. Gard..

[73] Kanehisa M, Goto S (2000). KEGG: Kyoto Encyclopedia of Genes and Genomes. Nucleic Acids Res..

[74] Goyer A (2010). Thiamine in plants: aspects of its metabolism and functions. Phytochemistry.

[75] Rapala-Kozik M, Kowalska E, Ostrowska K (2008). Modulation of thiamine metabolism in *Zea mays* seedlings under conditions of abiotic stress. J. Exp. Bot..

[76] Takagi H (2016). Allantoin, a stress-related purine metabolite, can activate jasmonate signaling in a MYC2-regulated and abscisic acid-dependent manner. J. Exp. Bot..

[77] Lenka SK, Katiyar A, Chinnusamy V, Bansal KC (2011). Comparative analysis of drought-responsive transcriptome in *Indica* rice genotypes with contrasting drought tolerance. Plant Biotechnol. J..

[78] Wang A (2017). A sweet potato cinnamate 4-hydroxylase gene, IbC4H, increases phenolics content and enhances drought tolerance in tobacco. Acta Physiol. Plant..

[79] Cheng S (2018). Characterization and expression patterns of a cinnamate-4-hydroxylase gene involved in lignin biosynthesis and in response to various stresses and hormonal treatments in *Ginkgo biloba*. Acta Physiol. Plant..

[80] Cass CL (2015). Effects of PHENYLALANINE AMMONIA LYASE (PAL) knockdown on cell wall composition, biomass digestibility, and biotic and abiotic stress responses in. Brachypodium. J. Exp. Bot..

[81] Chen Z, Zheng Z, Huang J, Lai Z, Fan B (2009). Biosynthesis of salicylic acid in plants. Plant Signal. Behav..

[82] Kumar D (2014). Salicylic acid signaling in disease resistance. Plant Sci..

[83] Khan MI, Fatma M, Per TS, Anjum NA, Khan NA (2015). Salicylic acid-induced abiotic stress tolerance and underlying mechanisms in plants. Front. Plant Sci..

[84] Hong Y, Zheng S, Wang X (2008). Dual functions of phospholipase Dα1 in plant response to drought. Mol. Plant.

[85] Arisz SA, Testerink C, Munnik T, Plant PA (2009). signaling via diacylglycerol kinase. Biochim. Biophys. Acta.

[86] Zhang Y (2009). Phospholipase Dα1 and phosphatidic acid regulate NADPH oxidase activity and production of reactive oxygen species in ABA-mediated stomatal closure in *Arabidopsis*. Plant Cell.

[87] Yu L (2010). Phosphatidic acid mediates salt stress response by regulation of MPK6 in *Arabidopsis thaliana*. New Phytol..

[88] Hou Q, Ufer G, Bartels D (2016). Lipid signalling in plant responses to abiotic stress. Plant Cell Environ..

[89] Levizou E, Drilias P, Kyparissis A (2004). Exceptional photosynthetic performance of *Capparis spinosa* L. under adverse conditions of Mediterranean summer. Photosynthetica.

[90] Meng LS, Yao SQ (2015). Transcription co‐activator Arabidopsis ANGUSTIFOLIA3 (AN3) regulates water‐use efficiency and drought tolerance by modulating stomatal density and improving root architecture by the transrepression of YODA (YDA). Plant Biotechnol. J..

[91] Yoo CY, Hasegawa PM, Mickelbart MV (2011). Regulation of stomatal density by the GTL1 transcription factor for improving water use efficiency. Plant Signal. Behav..

[92] Xiang C (2017). A genetic pathway composed of EDT1/HDG11, ERECTA, and E2Fa loci regulates water use efficiency by modulating stomatal density. BioRxiv.

[93] Kang M (2017). Arabidopsis stress associated protein 9 mediates biotic and abiotic stress responsive ABA signaling via the proteasome pathway. Plant Cell Environ..

[94] Dixit A (2018). A stress‐associated protein, AtSAP13, from *Arabidopsis thaliana* provides tolerance to multiple abiotic stresses. Plant Cell Environ..

[95] Vij S, Tyagi AK (2006). Genome-wide analysis of the stress associated protein (SAP) gene family containing A20/AN1 zinc-finger (s) in rice and their phylogenetic relationship with Arabidopsis. Mol. Gen. Genom..

[96] Lloret A (2017). Dual regulation of water retention and cell growth by a stress-associated protein (SAP) gene in Prunus. Sci. Rep..

[97] Yoon SK (2018). Downregulation of stress-associated protein 1 (PagSAP1) increases salt stress tolerance in poplar (*Populus alba* × *P. glandulosa*). Trees.

[98] Jeszka-Skowron M, Sentkowska A, Pyrzynska K (2016). & Paz De Peña, M. Chlorogenic acids, caffeine content and antioxidant properties of green coffee extracts - influence of green coffee bean preparation. Eur. Food Res. Technol..

[99] Mithen RF, Dekker M, Verkerk R, Rabot S, Johnson IT (2000). The nutritional significance, biosynthesis and bioavailability of glucosinolates in human foods. J. Sci. Food Agr..

[100] Naing AH (2017). Overexpression of snapdragon Delila (Del) gene in tobacco enhances anthocyanin accumulation and abiotic stress tolerance. BMC Plant Biol..

[101] Inocencio C, Cowan RS, Alcaraz F, Rivera D, Fay MF (2005). AFLP fingerprinting in *Capparis* subgenus *Capparis* related to the commercial sources of capers. Gen. Res. Crop. Evol..

[102] Özbek Ö, Kara A (2013). Genetic variation in natural populations of *Capparis* from Turkey, as revealed by RAPD analysis. Plant Syst. Evol..

[103] Wei W (2011). Characterization of the sesame (*Sesamum indicum* L.) global transcriptome using Illumina paired-end sequencing and development of EST-SSR markers. BMC Genomics.

[104] Jiang B, Xie D, Liu W, Peng Q, He X (2013). De Novo assembly and characterization of the transcriptome, and development of SSR Markers in Wax Gourd (*Benincasa hispida*). PLoS One.

[105] Hu L (2015). De Novo assembly and characterization of fruit transcriptome in black pepper (*Piper nigrum*). PLoS One.

[106] Varshney RK, Graner A, Sorrells ME (2005). Genic microsatellite markers in plants: features and applications. Trends Biotechnol..

[107] Ramsay L (1999). Intimate association of microsatellite repeats with retrotransposons and other dispersed repetitive elements in barley. Plant J..

[108] La Rota M, Kantety RS, Yu JK, Sorrells ME (2005). Nonrandom distribution and frequencies of genomic and EST-derived microsatellite markers in rice, wheat, and barley. BMC Genomics.

[109] Xie F, Sun G, Stiller JW, Zhang B (2011). Genome-wide functional analysis of the cotton transcriptome by creating an integrated EST database. PLoS One.

[110] Mercati F (2013). Single nucleotide polymorphism isolated from a novel EST dataset in garden asparagus (*Asparagus officinalis* L.). Plant Sci..

[111] Zeng S (2010). Development of a EST dataset and characterization of EST-SSRs in a traditional Chinese medicinal plant, *Epimedium sagittatum* (Sieb. Et Zucc.) Maxim. BMC Genomics.

[112] Barbera G, Di Lorenzo R, Barone E (1991). Observations on *Capparis* populations cultivated in Sicily and on their vegetative and productive behaviour. Agric. Mediterr..

[113] MacManes MD (2014). On the optimal trimming of high-throughput mRNA sequence data. Front. Genet..

[114] Mbandi SK, Hesse U, Rees DJG, Christoffels A (2014). A glance at quality score: implication for *de novo* transcriptome reconstruction of Illumina reads. Front. Genet..

[115] Bolger AM, Lohse M, Usadel B (2014). Trimmomatic: A flexible trimmer for Illumina Sequence Data. Bioinformatics.

[116] Grabherr MG (2011). Full-length transcriptome assembly from RNA-Seq data without a reference genome. Nat. Biotech..

[117] Huang Y, Niu B, Gao Y, Fu L, Li W (2010). CD-HIT Suite: a web server for clustering and comparing biological sequences. Bioinformatics.

[118] Simão FA, Waterhouse RM, Ioannidis P, Kriventseva EV, Zdobnov EM (2015). BUSCO: assessing genome assembly and annotation completeness with single-copy orthologs. Bioinformatics.

[119] Gurevich A, Saveliev V, Vyahhi N, Tesler G (2013). QUAST: quality assessment tool for genome assemblies. Bioinformatics.

[120] Bryant DM (2017). A tissue-mapped axolotl de novo transcriptome enables identification of limb regeneration factors. Cell Rep..

[121] Finn RD, Clements J, Eddy SR (2011). HMMER web server: interactive sequence similarity searching. Nucleic Acids Res..

[122] Punta, M. *et al*. The Pfam protein families database. *Nucleic Acids Res*. **40** (Database issue), D290–D301; 10.1093/nar/gkr1065 (2012). 10.1093/nar/gkr1065 PMC3245129 22127870

[123] Petersen TN, Brunak S, von Heijne G, Nielsen H (2011). SignalP 4.0: discriminating signal peptides from transmembrane regions. Nat. Methods..

[124] Hu ZL, Bao J, Reecy JM (2008). CateGOrizer: A Web-Based Program to Batch Analyze Gene Ontology Classification Categories. Online J. Bioinform..

[125] Moriya Y, Itoh M, Okuda S, Yoshizawa AC, Kanehisa M (2007). KAAS: an automatic genome annotation and pathway reconstruction server. Nucleic Acids Res..

[126] Okuda S (2008). KEGG Atlas mapping for global analysis of metabolic pathways. Nucleic Acids Res..

[127] Kanehisa M (2019). New approach for understanding genome variations in KEGG. Nucleic Acids Res..

[128] Kanehisa M (2017). KEGG: new perspectives on genomes, pathways, diseases and drugs. Nucleic Acids Res..

[129] Altschul SF, Gish W, Miller W, Myers EW, Lipman DJ (1990). Basic local alignment search tool. J. Mol. Biol..

[130] Ellis JR, Burke JM (2007). EST-SSRs as a resource for population genetic analyses. Heredity.

[131] Beier S, Thiel T, Münch T, Scholz U, Mascher M (2017). MISA-web: a web server for microsatellite prediction. Bioinformatics.

[132] Untergasser A (2012). Primer3–new capabilities and interfaces. Nucleic Acids Res..

[133] Lodhi MA, Ye G, Weeden NF, Reisch BI (1994). A simple and efficient method for DNA extraction from grapevine cultivars and *Vitis* species. Mol. Biol. Rep..

[134] Liu K, Muse SV (2005). PowerMarker: an integrated analysis environment for genetic marker analysis. Bioinformatics.

[135] Kamvar ZN, Tabima JF, Grünwald NJ (2014). Poppr: an R package for genetic analysis of populations with clonal, partially clonal, and/or sexual reproduction. PeerJ.

[136] Bruvo R, Michiels NK, D’Souza TG, Schulenburg H (2004). A simple method for the calculation of microsatellite genotype distances irrespective of ploidy level. Mol. Ecol..

[137] Jombart T, Ahmed I (2011). Adegenet 1.3-1: new tools for the analysis of genome-wide SNP data. Bioinformatics.

