# Supplementary material for: Transcriptome analysis and codominant markers development in caper, a drought tolerant orphan crop with medicinal value
Source: Sci Rep. 2019 Jul 18;9:10411. doi: 10.1038/s41598-019-46613-x (PMC6639398; doi:10.1038/s41598-019-46613-x)

Supplementary information

of the Manuscript:

Transcriptome analysis and codominant markers development in caper, a drought tolerant orphan crop with medicinal value

Francesco Mercati, Ignazio Fontana, Alessandro Silvestre Gristina, Adriana Martorana, Mahran El Nagar, Roberto De Michele, Silvio Fici, Francesco Carimi

Supplementary Tables S1-S5

Supplementary Figures S1-S2

Supplementary Table S1. Overview of KEGG pathways isolated in *C. spinosa* leaf transcriptome.

Pathways	#KO	#Seqs
Purine metabolism	76	669
Oxidative phosphorylation	60	414
Pyrimidine metabolism	56	504
Amino sugar and nucleotide sugar metabolism	34	436
Glycerophospholipid metabolism	33	465
Cysteine and methionine metabolism	32	271
Starch and sucrose metabolism	30	510
Glycolysis / Gluconeogenesis	28	397
Photosynthesis	28	79
Porphyrin and chlorophyll metabolism	28	167
Glycine serine and threonine metabolism	27	261
Glyoxylate and dicarboxylate metabolism	24	279
N-Glycan biosynthesis	24	191
Pyruvate metabolism	23	367
Alanine aspartate and glutamate metabolism	23	203
Carbon fixation in photosynthetic organisms	22	192
Glycerolipid metabolism	22	286
Phenylalanine tyrosine and tryptophan biosynthesis	20	179
Inositol phosphate metabolism	19	309
Various types of N-glycan biosynthesis	19	141
Citrate cycle TCA cycle)	18	214
Fructose and mannose metabolism	18	185
Arginine and proline metabolism	18	254
Ubiquinone and other terpenoid-quinone biosynthesis	18	122
Phenylpropanoid biosynthesis	18	316
Arginine biosynthesis	17	148
Terpenoid backbone biosynthesis	17	155
Glutathione metabolism	16	237
Pentose phosphate pathway	15	159
Galactose metabolism	15	176
Methane metabolism	15	177
Steroid biosynthesis	15	118
Glycosylphosphatidylinositol (GPI)-anchor biosynthesis	15	52
Pantothenate and CoA biosynthesis	15	108
Valine leucine and isoleucine degradation	14	184
Tyrosine metabolism	14	134
Tryptophan metabolism	14	136
beta-Alanine metabolism	14	133
Ascorbate and aldarate metabolism	13	171
Propanoate metabolism	13	134
Phenylalanine metabolism	13	133
Fatty acid biosynthesis	12	166
Nitrogen metabolism	12	110
alpha-Linolenic acid metabolism	12	163
Carotenoid biosynthesis	12	126
Carbon fixation pathways in prokaryotes	11	122
Nicotinate and nicotinamide metabolism	11	128
Fatty acid degradation	10	216
Sphingolipid metabolism	10	122
Thiamine metabolism	10	63

Supplementary Table S1. Overview of KEGG pathways isolated in *C. spinosa* leaf transcriptome.

Pathways	#KO	#Seqs
Folate biosynthesis	10	93
Diterpenoid biosynthesis	10	41
Drug metabolism - other enzymes	10	154
Sulfur metabolism	9	113
Butanoate metabolism	9	67
Cutin suberine and wax biosynthesis	9	37
Valine leucine and isoleucine biosynthesis	9	76
Lysine degradation	9	133
Riboflavin metabolism	9	52
Isoquinoline alkaloid biosynthesis	9	88
Glucosinolate biosynthesis	9	36
Pentose and glucuronate interconversions	8	104
Ether lipid metabolism	8	178
Lysine biosynthesis	8	84
Cyanoamino acid metabolism	8	197
One carbon pool by folate	8	79
Photosynthesis - antenna proteins	7	17
Biosynthesis of unsaturated fatty acids	7	95
Histidine metabolism	7	88
Selenocompound metabolism	7	74
Other glycan degradation	7	93
Vitamin B6 metabolism	7	42
Flavonoid biosynthesis	7	35
Tropane piperidine and pyridine alkaloid biosynthesis	7	80
Fatty acid elongation	6	49
Lipopolysaccharide biosynthesis	6	49
Arachidonic acid metabolism	5	44
Other types of O-glycan biosynthesis	5	58
Biotin metabolism	5	54
Monoterpenoid biosynthesis	5	12
Zeatin biosynthesis	5	61
Monobactam biosynthesis	5	64
C5-Branched dibasic acid metabolism	4	24
Linoleic acid metabolism	4	72
Retinol metabolism	4	38
Sesquiterpenoid and triterpenoid biosynthesis	4	52
Brassinosteroid biosynthesis	4	17
Stilbenoid diarylheptanoid and gingerol biosynthesis	4	21
Streptomycin biosynthesis	4	55
Styrene degradation	4	43
Taurine and hypotaurine metabolism	3	42
Glycosaminoglycan degradation	3	22
Chloroalkane and chloroalkene degradation	3	54
Metabolism of xenobiotics by cytochrome P450	3	82
Drug metabolism - cytochrome P450	3	82
Synthesis and degradation of ketone bodies	2	24
Primary bile acid biosynthesis	2	9
Steroid hormone biosynthesis	2	9
Phosphonate and phosphinate metabolism	2	57
Glycosphingolipid biosynthesis - globo and isoglobo series	2	20

Supplementary Table S1. Overview of KEGG pathways isolated in *C. spinosa* leaf transcriptome.

Pathways	#KO	#Seqs
Glycosphingolipid biosynthesis - ganglio series	2	12
Lipoic acid metabolism	2	14
Anthocyanin biosynthesis	2	3
Isoflavonoid biosynthesis	2	4
Indole alkaloid biosynthesis	2	7
Phenazine biosynthesis	2	16
Prodigiosin biosynthesis	2	21
Benzoate degradation	2	20
Naphthalene degradation	2	18
D-Glutamine and D-glutamate metabolism	1	11
Peptidoglycan biosynthesis	1	3
Insect hormone biosynthesis	1	36
Limonene and pinene degradation	1	36
Biosynthesis of ansamycins	1	8
Polyketide sugar unit biosynthesis	1	2
Biosynthesis of siderophore group nonribosomal peptides	1	8
Flavone and flavonol biosynthesis	1	2
Caffeine metabolism	1	4
Neomycin kanamycin and gentamicin biosynthesis	1	14
Novobiocin biosynthesis	1	5
Aflatoxin biosynthesis	1	5
Aminobenzoate degradation	1	16
Fluorobenzoate degradation	1	4
Chlorocyclohexane and chlorobenzene degradation	1	4
Toluene degradation	1	4
Atrazine degradation	1	11
Biosynthesis of terpenoids and steroids	1	12

Supplementary Table S2. Frequencies of different repeat motifs in EST-SSRs from *Capparis spinosa* leaf transcriptome.

Repeats	4	5	6	7	8	9	10	11	12	13	14	15	16	17	18	19	20	21	22	23	24	25	26	27	28	29	30	31	Total	Total (%)	Within motifs (%)
AC	-	-	-	-	-	-	-	-	-	-	-	-	-	-	-	-	3	1	-	-	2	-	1	-	-	-	-	-	7	0.14	1.93
AG	-	-	-	-	-	-	-	-	-	-	-	-	-	-	-	-	15	1	11	3	9	2	8	1	-	-	-	-	50	1.00	13.81
AT	-	-	-	-	-	-	-	-	-	-	-	-	-	-	-	-	5	-	2	-	-	1	-	-	-	-	-	-	8	0.16	2.21
CA	-	-	-	-	-	-	-	-	-	-	-	-	-	-	-	-	2	-	-	-	-	-	-	-	-	-	-	-	2	0.04	0.55
CT	-	-	-	-	-	-	-	-	-	-	-	-	-	-	-	-	26	15	27	19	7	4	-	1	3	-	-	-	102	2.04	28.18
GA	-	-	-	-	-	-	-	-	-	-	-	-	-	-	-	-	5	10	7	8	2	-	-	2	-	-	-	-	34	0.68	9.39
TA	-	-	-	-	-	-	-	-	-	-	-	-	-	-	-	-	3	1	1	-	-	-	-	-	-	-	-	-	5	0.10	1.38
TC	-	-	-	-	-	-	-	-	-	-	-	-	-	-	-	-	70	32	20	9	6	6	5	1	-	-	-	1	150	2.99	41.44
TG	-	-	-	-	-	-	-	-	-	-	-	-	-	-	-	-	2	1	-	-	-	-	1	-	-	-	-	-	4	0.08	1.10
**Tot. Dinucleotide**																													**362**	**7.22**	
AAC	-	-	-	13	5	2	-	4	-	-	-	3	-	-	-	-	-	-	-	-	-	-	-	-	-	-	-	-	27	0.54	0.98
AAG	-	-	-	81	61	4	22	1	1	5	-	-	2	1	9	-	-	-	-	-	-	-	-	-	-	-	-	-	187	3.73	6.79
AAT	-	-	-	5	3	1	-	1	1	-	-	1	-	-	-	-	-	-	-	-	-	-	-	-	-	-	-	-	12	0.24	0.44
ACA	-	-	-	2	5	6	2	-	2	-	-	-	-	-	-	-	-	-	-	-	-	-	-	-	-	-	-	-	17	0.34	0.62
ACC	-	-	-	5	3	3	-	-	-	2	-	-	-	-	-	-	-	-	-	-	-	-	-	-	-	-	-	-	13	0.26	0.47
ACG	-	-	-	1	8	4	-	-	-	-	-	-	-	-	-	-	-	-	-	-	-	-	-	-	-	-	-	-	13	0.26	0.47
AGA	-	-	-	49	70	6	9	15	6	1	2	7	2	-	2	1	-	-	-	-	-	-	-	-	-	-	-	-	170	3.39	6.17
AGC	-	-	-	9	24	3	1	-	-	8	-	-	-	-	-	-	-	-	-	-	-	-	-	-	-	-	-	-	45	0.90	1.63
AGG	-	-	-	18	7	4	-	2	4	-	-	-	-	-	-	-	-	-	-	-	-	-	-	-	-	-	-	-	35	0.70	1.27
AGT	-	-	-	5	-	-	-	-	-	-	-	-	-	-	-	-	-	-	-	-	-	-	-	-	-	-	-	-	5	0.10	0.18
ATA	-	-	-	13	5	4	4	-	1	-	1	-	-	-	-	-	-	-	-	-	-	-	-	-	-	-	-	-	28	0.56	1.02
ATC	-	-	-	10	9	2	2	2	2	-	-	-	-	-	-	-	-	-	-	-	-	-	-	-	-	-	-	-	27	0.54	0.98
ATG	-	-	-	14	10	1	-	8	6	2	2	-	-	-	-	-	-	-	-	-	-	-	-	-	-	-	-	-	43	0.86	1.56
ATT	-	-	-	27	20	1	6	2	3	-	-	-	-	-	-	-	-	-	-	-	-	-	-	-	-	-	-	-	59	1.18	2.14
CAA	-	-	-	13	10	-	-	-	4	1	-	-	-	-	-	-	-	-	-	-	-	-	-	-	-	-	-	-	28	0.56	1.02
CAC	-	-	-	3	2	5	3	-	-	-	-	-	-	-	-	-	-	-	-	-	-	-	-	-	-	-	-	-	13	0.26	0.47
CAG	-	-	-	14	32	13	10	1	3	-	2	1	1	3	-	-	-	-	-	-	-	-	-	-	-	-	-	-	80	1.60	2.90
CAT	-	-	-	17	16	5	8	2	7	-	-	-	-	-	-	-	-	-	-	-	-	-	-	-	-	-	-	-	55	1.10	2.00
CCA	-	-	-	33	7	4	-	-	1	-	4	-	-	-	-	-	-	-	-	-	-	-	-	-	-	-	-	-	49	0.98	1.78
CCG	-	-	-	4	2	1	8	-	-	-	-	-	-	-	-	-	-	-	-	-	-	-	-	-	-	-	-	-	15	0.30	0.54
CCT	-	-	-	8	13	3	12	3	-	1	-	-	-	-	-	-	-	-	-	-	-	-	-	-	-	-	-	-	40	0.80	1.45
CGA	-	-	-	1	3	-	-	-	-	-	-	-	-	-	-	-	-	-	-	-	-	-	-	-	-	-	-	-	4	0.08	0.15
CGC	-	-	-	3	-	1	-	-	-	-	-	-	-	-	-	-	-	-	-	-	-	-	-	-	-	-	-	-	4	0.08	0.15
CGG	-	-	-	1	1	-	2	-	-	-	-	-	-	-	-	-	-	-	-	-	-	-	-	-	-	-	-	-	4	0.08	0.15
CTA	-	-	-	5	-	-	-	-	-	-	-	-	-	-	-	-	-	-	-	-	-	-	-	-	-	-	-	-	5	0.10	0.18
CTC	-	-	-	18	21	10	3	9	3	-	-	-	-	-	-	-	-	-	-	-	-	-	-	-	-	-	-	-	64	1.28	2.32
CTG	-	-	-	21	10	-	1	-	1	-	-	-	-	-	-	-	-	-	-	-	-	-	-	-	-	-	-	-	33	0.66	1.20
CTT	-	-	-	84	53	17	5	6	10	10	2	-	4	3	-	-	-	-	-	-	-	-	-	-	-	-	-	-	194	3.87	7.04
GAA	-	-	-	108	74	18	23	20	6	14	2	3	1	2	1	-	-	-	-	-	-	-	-	-	-	-	-	-	272	5.43	9.87
GAC	-	-	-	9	4	-	-	-	-	-	-	-	-	-	-	-	-	-	-	-	-	-	-	-	-	-	-	-	13	0.26	0.47
GAG	-	-	-	38	16	2	-	1	-	-	-	-	-	-	-	-	-	-	-	-	-	-	-	-	-	-	-	-	57	1.14	2.07
GAT	-	-	-	21	31	5	4	-	1	-	-	-	-	-	-	-	-	-	-	-	-	-	-	-	-	-	-	-	62	1.24	2.25
GCA	-	-	-	5	10	3	6	7	1	-	-	-	-	-	-	-	-	-	-	-	-	-	-	-	-	-	-	-	32	0.64	1.16
GCC	-	-	-	7	2	4	-	-	-	-	-	-	-	-	-	-	-	-	-	-	-	-	-	-	-	-	-	-	13	0.26	0.47
GCG	-	-	-	15	10	-	-	-	-	-	-	-	-	-	-	-	-	-	-	-	-	-	-	-	-	-	-	-	25	0.50	0.91
GCT	-	-	-	3	3	-	-	4	-	-	-	-	-	-	-	-	-	-	-	-	-	-	-	-	-	-	-	-	10	0.20	0.36
GGA	-	-	-	21	36	-	-	1	-	-	-	-	-	-	-	-	-	-	-	-	-	-	-	-	-	-	-	-	58	1.16	2.10
GGC	-	-	-	14	1	-	-	-	-	-	-	-	-	-	-	-	-	-	-	-	-	-	-	-	-	-	-	-	15	0.30	0.54
GGT	-	-	-	6	1	2	1	-	-	-	-	-	-	-	-	-	-	-	-	-	-	-	-	-	-	-	-	-	10	0.20	0.36
GTC	-	-	-	2	-	-	-	-	-	-	-	-	-	-	-	-	-	-	-	-	-	-	-	-	-	-	-	-	2	0.04	0.07
GTG	-	-	-	-	-	2	-	-	-	-	-	-	-	-	-	-	-	-	-	-	-	-	-	-	-	-	-	-	2	0.04	0.07
GTT	-	-	-	1	3	-	-	-	-	-	-	-	-	-	-	-	-	-	-	-	-	-	-	-	-	-	-	-	4	0.08	0.15
TAA	-	-	-	5	10	-	1	1	-	-	-	-	-	-	-	-	-	-	-	-	-	-	-	-	-	-	-	-	17	0.34	0.62
TAC	-	-	-	2	-	-	-	-	-	-	-	-	-	-	-	-	-	-	-	-	-	-	-	-	-	-	-	-	2	0.04	0.07
TAT	-	-	-	12	1	1	3	1	-	-	-	2	-	-	-	-	-	-	-	-	-	-	-	-	-	-	-	-	20	0.40	0.73
TCA	-	-	-	25	15	1	9	2	2	4	-	4	-	-	-	-	-	-	-	-	-	-	-	-	-	-	-	-	62	1.24	2.25
TCC	-	-	-	35	6	2	2	-	-	-	-	-	-	-	-	-	-	-	-	-	-	-	-	-	-	-	-	-	45	0.90	1.63
TCG	-	-	-	11	1	-	-	-	-	-	-	1	-	-	-	-	-	-	-	-	-	-	-	-	-	-	-	-	13	0.26	0.47
TCT	-	-	-	113	79	30	37	15	18	8	4	8	4	3	-	-	1	-	-	-	-	-	-	-	-	-	-	-	320	6.39	11.61
TGA	-	-	-	18	30	10	1	1	1	3	-	1	1	-	1	-	-	-	1	-	-	-	-	-	-	-	-	-	68	1.36	2.47
TGC	-	-	-	8	-	-	-	-	-	-	-	-	-	-	-	-	-	-	-	-	-	-	-	-	-	-	-	-	8	0.16	0.29
TGG	-	-	-	1	1	1	-	-	-	-	-	-	-	-	-	-	-	-	-	-	-	-	-	-	-	-	-	-	3	0.06	0.11
TGT	-	-	-	6	1	-	-	-	5	-	-	-	-	-	-	-	-	-	-	-	-	-	-	-	-	-	-	-	12	0.24	0.44
TTA	-	-	-	11	-	2	1	7	-	1	-	1	-	-	-	-	-	-	-	-	-	-	-	-	-	-	-	-	23	0.46	0.83
TTC	-	-	-	90	65	44	53	27	10	6	5	2	-	-	-	1	-	-	-	-	-	-	-	-	-	-	-	-	303	6.05	10.99
TTG	-	-	-	6	10	-	-	-	-	-	-	-	-	-	-	-	-	-	-	-	-	-	-	-	-	-	-	-	16	0.32	0.58
**Tot. Trinucleotide**																													**2756**	**55.02**	
AAAC	-	3	-	-	-	-	-	-	-	-	-	-	-	-	-	-	-	-	-	-	-	-	-	-	-	-	-	-	3	0.06	0.53
AAAG	-	10	1	-	-	-	-	-	-	-	-	-	-	-	-	-	-	-	-	-	-	-	-	-	-	-	-	-	11	0.22	1.94
AAAT	-	19	14	-	2	-	-	-	-	-	-	-	-	-	-	-	-	-	-	-	-	-	-	-	-	-	-	-	35	0.70	6.18
AACA	-	8	-	-	-	-	-	-	-	-	-	-	-	-	-	-	-	-	-	-	-	-	-	-	-	-	-	-	8	0.16	1.41
AAGA	-	1	7	1	-	-	-	-	-	4	-	-	-	-	-	-	-	-	-	-	-	-	-	-	-	-	-	-	13	0.26	2.30
AATA	-	6	2	1	-	-	-	-	-	-	-	-	-	-	-	-	-	-	-	-	-	-	-	-	-	-	-	-	9	0.18	1.59
AATG	-	7	-	-	-	-	-	-	-	-	-	-	-	-	-	-	-	-	-	-	-	-	-	-	-	-	-	-	7	0.14	1.24
AATT	-	5	-	-	-	-	-	-	-	-	-	-	-	-	-	-	-	-	-	-	-	-	-	-	-	-	-	-	5	0.10	0.88
ACAA	-	-	2	-	-	-	-	-	-	-	-	-	-	-	-	-	-	-	-	-	-	-	-	-	-	-	-	-	2	0.04	0.35
ACAG	-	1	8	-	-	-	-	-	-	-	-	-	-	-	-	-	-	-	-	-	-	-	-	-	-	-	-	-	9	0.18	1.59
ACAT	-	4	2	2	1	-	2	-	1	-	-	-	-	-	-	-	-	-	-	-	-	-	-	-	-	-	-	-	12	0.24	2.12
AGAA	-	7	4	-	-	-	-	-	-	-	-	-	-	-	-	-	-	-	-	-	-	-	-	-	-	-	-	-	11	0.22	1.94
AGAC	-	1	-	-	-	-	-	-	-	-	-	-	-	-	-	-	-	-	-	-	-	-	-	-	-	-	-	-	1	0.02	0.18
AGAT	-	1	-	1	-	-	-	-	-	-	-	-	-	-	-	-	-	-	-	-	-	-	-	-	-	-	-	-	2	0.04	0.35
AGCG	-	-	6	-	-	-	-	-	-	-	-	-	-	-	-	-	-	-	-	-	-	-	-	-	-	-	-	-	6	0.12	1.06
AGGA	-	1	-	-	-	-	-	-	-	-	-	-	-	-	-	-	-	-	-	-	-	-	-	-	-	-	-	-	1	0.02	0.18
ATAA	-	8	1	-	-	-	-	-	-	-	-	-	-	-	-	-	-	-	-	-	-	-	-	-	-	-	-	-	9	0.18	1.59
ATAC	-	7	5	-	2	-	-	1	-	-	-	-	1	-	-	-	-	-	-	-	-	-	-	-	-	-	-	-	16	0.32	2.83
ATAG	-	3	-	-	-	-	-	-	-	-	-	-	-	-	-	-	-	-	-	-	-	-	-	-	-	-	-	-	3	0.06	0.53
ATCG	-	2	-	-	-	-	-	-	-	-	-	-	-	-	-	-	-	-	-	-	-	-	-	-	-	-	-	-	2	0.04	0.35
ATCT	-	-	-	-	-	10	-	1	-	1	-	-	-	-	-	-	-	-	-	-	-	-	-	-	-	-	-	-	12	0.24	2.12
ATGA	-	4	-	-	-	-	-	-	-	-	-	-	-	-	-	-	-	-	-	-	-	-	-	-	-	-	-	-	4	0.08	0.71
ATGT	-	6	3	-	-	-	-	-	-	-	-	-	-	-	-	-	-	-	-	-	-	-	-	-	-	-	-	-	9	0.18	1.59
ATTA	-	1	-	-	-	-	-	-	-	-	-	-	-	-	-	-	-	-	-	-	-	-	-	-	-	-	-	-	1	0.02	0.18
ATTG	-	-	1	-	-	-	-	-	-	-	-	-	-	-	-	-	-	-	-	-	-	-	-	-	-	-	-	-	1	0.02	0.18
ATTT	-	12	11	-	-	-	-	-	-	-	-	-	-	-	-	-	-	-	-	-	-	-	-	-	-	-	-	-	23	0.46	4.06
CAAA	-	2	-	-	-	-	-	-	-	-	-	-	-	-	-	-	-	-	-	-	-	-	-	-	-	-	-	-	2	0.04	0.35
CACG	-	1	-	-	-	-	-	-	-	-	-	-	-	-	-	-	-	-	-	-	-	-	-	-	-	-	-	-	1	0.02	0.18
CATA	-	6	-	-	-	1	-	-	-	-	-	-	-	-	-	-	-	-	-	-	-	-	-	-	-	-	-	-	7	0.14	1.24
CATG	-	-	1	-	-	-	-	-	-	-	-	-	-	-	-	-	-	-	-	-	-	-	-	-	-	-	-	-	1	0.02	0.18
CATT	-	-	1	-	-	-	-	-	-	-	-	-	-	-	-	-	-	-	-	-	-	-	-	-	-	-	-	-	1	0.02	0.18
CCAG	-	-	1	-	-	-	-	-	-	-	-	-	-	-	-	-	-	-	-	-	-	-	-	-	-	-	-	-	1	0.02	0.18
CCGA	-	-	6	-	-	-	-	-	-	-	-	-	-	-	-	-	-	-	-	-	-	-	-	-	-	-	-	-	6	0.12	1.06
CGAT	-	1	6	-	-	-	-	-	-	-	-	-	-	-	-	-	-	-	-	-	-	-	-	-	-	-	-	-	7	0.14	1.24

Supplementary Table S2. Frequencies of different repeat motifs in EST-SSRs from *Capparis spinosa* leaf transcriptome.

Repeats	4	5	6	7	8	9	10	11	12	13	14	15	16	17	18	19	20	21	22	23	24	25	26	27	28	29	30	31	Total	Total (%)	Within motifs (%)
CGCA	-	1	-	-	-	-	-	-	-	-	-	-	-	-	-	-	-	-	-	-	-	-	-	-	-	-	-	-	1	0.02	0.18
CTAG	-	-	2	-	-	-	-	-	-	-	-	-	-	-	-	-	-	-	-	-	-	-	-	-	-	-	-	-	2	0.04	0.35
CTAT	-	3	-	-	-	-	-	-	-	-	-	-	-	-	-	-	-	-	-	-	-	-	-	-	-	-	-	-	3	0.06	0.53
CTCC	-	1	-	-	-	-	-	-	-	-	-	-	-	-	-	-	-	-	-	-	-	-	-	-	-	-	-	-	1	0.02	0.18
CTGT	-	1	-	-	-	-	-	-	-	-	-	-	-	-	-	-	-	-	-	-	-	-	-	-	-	-	-	-	1	0.02	0.18
CTTT	-	5	6	-	-	1	-	-	-	-	-	-	-	-	-	-	-	-	-	-	-	-	-	-	-	-	-	-	12	0.24	2.12
GAAA	-	4	2	-	-	-	-	-	-	-	-	-	-	-	-	-	-	-	-	-	-	-	-	-	-	-	-	-	6	0.12	1.06
GAAT	-	2	-	-	-	-	-	-	-	-	-	-	-	-	-	-	-	-	-	-	-	-	-	-	-	-	-	-	2	0.04	0.35
GACA	-	1	-	-	-	-	-	-	-	-	-	-	-	-	-	-	-	-	-	-	-	-	-	-	-	-	-	-	1	0.02	0.18
GACT	-	-	2	-	-	-	-	-	-	-	-	-	-	-	-	-	-	-	-	-	-	-	-	-	-	-	-	-	2	0.04	0.35
GAGC	-	-	1	-	-	-	-	-	-	-	-	-	-	-	-	-	-	-	-	-	-	-	-	-	-	-	-	-	1	0.02	0.18
GATA	-	1	-	-	-	-	-	-	-	-	-	-	-	-	-	-	-	-	-	-	-	-	-	-	-	-	-	-	1	0.02	0.18
GATC	-	1	-	-	-	-	-	-	-	-	-	-	-	-	-	-	-	-	-	-	-	-	-	-	-	-	-	-	1	0.02	0.18
GATT	-	3	-	-	-	-	-	-	-	-	-	-	-	-	-	-	-	-	-	-	-	-	-	-	-	-	-	-	3	0.06	0.53
GCAT	-	-	1	-	-	-	-	-	-	-	-	-	-	-	-	-	-	-	-	-	-	-	-	-	-	-	-	-	1	0.02	0.18
GCTC	-	7	-	-	-	-	-	-	-	-	-	-	-	-	-	-	-	-	-	-	-	-	-	-	-	-	-	-	7	0.14	1.24
GTAT	-	14	-	-	1	-	-	-	-	-	-	-	-	-	-	-	-	-	-	-	-	-	-	-	-	-	-	-	15	0.30	2.65
GTCC	-	1	-	-	-	-	-	-	-	-	-	-	-	-	-	-	-	-	-	-	-	-	-	-	-	-	-	-	1	0.02	0.18
GTTC	-	1	-	-	-	-	-	-	-	-	-	-	-	-	-	-	-	-	-	-	-	-	-	-	-	-	-	-	1	0.02	0.18
GTTT	-	5	1	1	-	-	-	-	-	-	-	-	-	-	-	-	-	-	-	-	-	-	-	-	-	-	-	-	7	0.14	1.24
TAAA	-	1	1	1	-	-	-	-	-	-	-	-	-	-	-	-	-	-	-	-	-	-	-	-	-	-	-	-	3	0.06	0.53
TAAT	-	5	-	-	-	-	-	-	-	-	-	-	-	-	-	-	-	-	-	-	-	-	-	-	-	-	-	-	5	0.10	0.88
TACA	-	2	3	1	-	-	-	-	-	1	-	-	-	-	-	-	-	-	-	-	-	-	-	-	-	-	-	-	7	0.14	1.24
TAGA	-	-	1	-	-	-	-	-	-	-	-	-	-	-	-	-	-	-	-	-	-	-	-	-	-	-	-	-	1	0.02	0.18
TATG	-	13	7	1	-	1	-	-	-	1	-	-	-	-	-	-	-	-	-	-	-	-	-	-	-	-	-	-	23	0.46	4.06
TATT	-	10	1	-	-	-	-	-	-	-	-	-	-	-	-	-	-	-	-	-	-	-	-	-	-	-	-	-	11	0.22	1.94
TCAA	-	4	-	-	-	-	-	-	-	-	-	-	-	-	-	-	-	-	-	-	-	-	-	-	-	-	-	-	4	0.08	0.71
TCAC	-	-	-	1	-	-	-	-	-	-	-	-	-	-	-	-	-	-	-	-	-	-	-	-	-	-	-	-	1	0.02	0.18
TCCT	-	13	-	-	-	-	-	-	-	-	-	-	-	-	-	-	-	-	-	-	-	-	-	-	-	-	-	-	13	0.26	2.30
TCTA	-	4	-	-	-	-	-	-	-	-	-	-	-	-	-	-	-	-	-	-	-	-	-	-	-	-	-	-	4	0.08	0.71
TCTG	-	1	-	-	-	-	-	-	-	-	-	-	-	-	-	-	-	-	-	-	-	-	-	-	-	-	-	-	1	0.02	0.18
TCTT	-	6	8	1	3	-	-	-	-	-	-	-	-	-	-	-	-	-	-	-	-	-	-	-	-	-	-	-	18	0.36	3.18
TGAA	-	3	1	-	-	-	-	-	-	-	-	-	-	-	-	-	-	-	-	-	-	-	-	-	-	-	-	-	4	0.08	0.71
TGAT	-	-	1	-	1	-	-	-	-	-	-	-	-	-	-	-	-	-	-	-	-	-	-	-	-	-	-	-	2	0.04	0.35
TGGT	-	-	-	-	2	-	-	-	1	-	-	-	-	-	-	-	-	-	-	-	-	-	-	-	-	-	-	-	3	0.06	0.53
TGTA	-	1	4	-	1	-	-	-	-	-	-	-	-	-	-	-	-	-	-	-	-	-	-	-	-	-	-	-	6	0.12	1.06
TGTC	-	1	-	-	-	-	-	-	-	-	-	-	-	-	-	-	-	-	-	-	-	-	-	-	-	-	-	-	1	0.02	0.18
TGTT	-	2	-	-	-	-	-	-	-	-	-	-	-	-	-	-	-	-	-	-	-	-	-	-	-	-	-	-	2	0.04	0.35
TTAA	-	14	-	-	-	-	-	-	-	-	-	-	-	-	-	-	-	-	-	-	-	-	-	-	-	-	-	-	14	0.28	2.47
TTAT	-	4	4	3	2	-	-	-	-	-	-	-	-	-	-	-	-	-	-	-	-	-	-	-	-	-	-	-	13	0.26	2.30
TTCT	-	4	2	1	-	4	-	-	-	-	-	-	-	-	-	-	-	-	-	-	-	-	-	-	-	-	-	-	11	0.22	1.94
TTGA	-	2	2	-	-	1	-	-	-	-	-	-	-	-	-	-	-	-	-	-	-	-	-	-	-	-	-	-	5	0.10	0.88
TTGG	-	3	-	-	-	-	-	-	-	-	-	-	-	-	-	-	-	-	-	-	-	-	-	-	-	-	-	-	3	0.06	0.53
TTGT	-	10	-	-	-	-	-	-	-	-	-	-	-	-	-	-	-	-	-	-	-	-	-	-	-	-	-	-	10	0.20	1.77
TTTA	-	24	4	-	-	-	-	-	-	-	-	-	-	-	-	-	-	-	-	-	-	-	-	-	-	-	-	-	28	0.56	4.95
TTTC	-	33	16	2	-	1	-	6	-	-	-	-	-	-	-	-	-	-	-	-	-	-	-	-	-	-	-	-	58	1.16	10.25
TTTG	-	5	-	-	-	-	-	-	-	-	-	-	-	-	-	-	-	-	-	-	-	-	-	-	-	-	-	-	5	0.10	0.88
**Tot. Tetranucleotide**																													**566**	**11.30**	
AAAAG	-	3	-	-	-	-	-	-	-	-	-	-	-	-	-	-	-	-	-	-	-	-	-	-	-	-	-	-	3	0.06	1.43
AAATA	-	2	-	-	-	-	-	-	-	-	-	-	-	-	-	-	-	-	-	-	-	-	-	-	-	-	-	-	2	0.04	0.95
AAATC	-	1	-	-	-	-	-	-	-	-	-	-	-	-	-	-	-	-	-	-	-	-	-	-	-	-	-	-	1	0.02	0.48
AACCC	-	2	-	-	-	-	-	-	-	-	-	-	-	-	-	-	-	-	-	-	-	-	-	-	-	-	-	-	2	0.04	0.95
AACCG	-	1	-	-	-	-	-	-	-	-	-	-	-	-	-	-	-	-	-	-	-	-	-	-	-	-	-	-	1	0.02	0.48
AAGAA	-	4	-	-	-	-	-	-	-	-	-	-	-	-	-	-	-	-	-	-	-	-	-	-	-	-	-	-	4	0.08	1.90
AAGAG	-	16	-	-	-	-	-	-	-	-	-	-	-	-	-	-	-	-	-	-	-	-	-	-	-	-	-	-	16	0.32	7.62
AATTT	-	2	-	-	-	-	-	-	-	-	-	-	-	-	-	-	-	-	-	-	-	-	-	-	-	-	-	-	2	0.04	0.95
AGAAG	-	1	1	-	-	-	-	-	-	-	-	-	-	-	-	-	-	-	-	-	-	-	-	-	-	-	-	-	2	0.04	0.95
AGCTG	-	1	-	-	-	-	-	-	-	-	-	-	-	-	-	-	-	-	-	-	-	-	-	-	-	-	-	-	1	0.02	0.48
ATAAT	-	-	-	2	-	-	-	-	-	-	-	-	-	-	-	-	-	-	-	-	-	-	-	-	-	-	-	-	2	0.04	0.95
ATCTC	-	-	-	1	-	-	-	-	-	-	-	-	-	-	-	-	-	-	-	-	-	-	-	-	-	-	-	-	1	0.02	0.48
ATTTT	-	1	-	-	-	-	-	-	-	-	-	-	-	-	-	-	-	-	-	-	-	-	-	-	-	-	-	-	1	0.02	0.48
CACAT	-	2	-	-	-	-	-	-	-	-	-	-	-	-	-	-	-	-	-	-	-	-	-	-	-	-	-	-	2	0.04	0.95
CCAAA	-	1	-	-	-	-	-	-	-	-	-	-	-	-	-	-	-	-	-	-	-	-	-	-	-	-	-	-	1	0.02	0.48
CCATG	-	3	-	-	-	-	-	-	-	-	-	-	-	-	-	-	-	-	-	-	-	-	-	-	-	-	-	-	3	0.06	1.43
CGGTT	-	5	-	-	-	-	-	-	-	-	-	-	-	-	-	-	-	-	-	-	-	-	-	-	-	-	-	-	5	0.10	2.38
CGTTT	-	1	-	-	-	-	-	-	-	-	-	-	-	-	-	-	-	-	-	-	-	-	-	-	-	-	-	-	1	0.02	0.48
CTTAA	-	-	-	-	-	2	-	-	-	-	-	-	-	-	-	-	-	-	-	-	-	-	-	-	-	-	-	-	2	0.04	0.95
CTTCT	-	3	-	-	-	-	-	-	-	-	-	-	-	-	-	-	-	-	-	-	-	-	-	-	-	-	-	-	3	0.06	1.43
CTTGC	-	6	-	-	-	-	-	-	-	-	-	-	-	-	-	-	-	-	-	-	-	-	-	-	-	-	-	-	6	0.12	2.86
GAAAA	-	4	-	-	-	-	-	-	-	-	-	-	-	-	-	-	-	-	-	-	-	-	-	-	-	-	-	-	4	0.08	1.90
GACCA	-	5	-	-	-	-	-	-	-	-	-	-	-	-	-	-	-	-	-	-	-	-	-	-	-	-	-	-	5	0.10	2.38
GACTG	-	-	1	1	-	-	-	-	-	-	-	-	-	-	-	-	-	-	-	-	-	-	-	-	-	-	-	-	2	0.04	0.95
GAGAG	-	10	-	-	-	-	-	-	-	-	-	-	-	-	-	-	-	-	-	-	-	-	-	-	-	-	-	-	10	0.20	4.76
GGATC	-	1	-	-	-	-	-	-	-	-	-	-	-	-	-	-	-	-	-	-	-	-	-	-	-	-	-	-	1	0.02	0.48
GGTCT	-	-	1	-	-	-	-	-	-	-	-	-	-	-	-	-	-	-	-	-	-	-	-	-	-	-	-	-	1	0.02	0.48
GTCCG	-	1	-	-	-	-	-	-	-	-	-	-	-	-	-	-	-	-	-	-	-	-	-	-	-	-	-	-	1	0.02	0.48
GTCTC	-	-	-	2	-	-	-	-	-	-	-	-	-	-	-	-	-	-	-	-	-	-	-	-	-	-	-	-	2	0.04	0.95
GTTGT	-	1	-	-	-	-	-	-	-	-	-	-	-	-	-	-	-	-	-	-	-	-	-	-	-	-	-	-	1	0.02	0.48
GTTTT	-	1	-	-	-	-	-	-	-	-	-	-	-	-	-	-	-	-	-	-	-	-	-	-	-	-	-	-	1	0.02	0.48
TATGG	-	-	-	-	8	-	-	-	-	-	-	-	-	-	-	-	-	-	-	-	-	-	-	-	-	-	-	-	8	0.16	3.81
TCACA	-	-	3	-	-	-	-	-	-	-	-	-	-	-	-	-	-	-	-	-	-	-	-	-	-	-	-	-	3	0.06	1.43
TCCTC	-	1	-	-	-	-	-	-	-	-	-	-	-	-	-	-	-	-	-	-	-	-	-	-	-	-	-	-	1	0.02	0.48
TCTCT	-	3	-	-	-	-	-	-	-	-	-	-	-	-	-	-	-	-	-	-	-	-	-	-	-	-	-	-	3	0.06	1.43
TCTGG	-	1	-	-	-	-	-	-	-	-	-	-	-	-	-	-	-	-	-	-	-	-	-	-	-	-	-	-	1	0.02	0.48
TCTTC	-	-	7	-	-	-	-	-	-	-	-	-	-	-	-	-	-	-	-	-	-	-	-	-	-	-	-	-	7	0.14	3.33
TCTTT	-	6	1	1	-	-	-	-	-	-	-	-	-	-	-	-	-	-	-	-	-	-	-	-	-	-	-	-	8	0.16	3.81
TGATT	-	1	-	-	-	-	-	-	-	-	-	-	-	-	-	-	-	-	-	-	-	-	-	-	-	-	-	-	1	0.02	0.48
TGCAG	-	-	-	-	-	1	-	-	-	-	-	-	-	-	-	-	-	-	-	-	-	-	-	-	-	-	-	-	1	0.02	0.48
TGTCG	-	1	-	-	-	-	-	-	-	-	-	-	-	-	-	-	-	-	-	-	-	-	-	-	-	-	-	-	1	0.02	0.48
TGTTC	-	1	-	-	-	-	-	-	-	-	-	-	-	-	-	-	-	-	-	-	-	-	-	-	-	-	-	-	1	0.02	0.48
TGTTT	-	1	3	-	-	-	-	-	-	-	-	-	-	-	-	-	-	-	-	-	-	-	-	-	-	-	-	-	4	0.08	1.90
TTAAA	-	1	-	-	-	-	-	-	-	-	-	-	-	-	-	-	-	-	-	-	-	-	-	-	-	-	-	-	1	0.02	0.48
TTAAG	-	-	-	-	-	1	-	-	-	-	-	-	-	-	-	-	-	-	-	-	-	-	-	-	-	-	-	-	1	0.02	0.48
TTATA	-	1	-	-	-	-	-	-	-	-	-	-	-	-	-	-	-	-	-	-	-	-	-	-	-	-	-	-	1	0.02	0.48
TTCCA	-	10	-	-	-	-	-	-	-	-	-	-	-	-	-	-	-	-	-	-	-	-	-	-	-	-	-	-	10	0.20	4.76
TTCCC	-	-	1	-	-	-	-	-	-	-	-	-	-	-	-	-	-	-	-	-	-	-	-	-	-	-	-	-	1	0.02	0.48
TTCTC	-	3	-	-	-	-	-	-	-	-	-	-	-	-	-	-	-	-	-	-	-	-	-	-	-	-	-	-	3	0.06	1.43
TTCTT	-	3	9	-	-	-	-	-	-	-	-	-	-	-	-	-	-	-	-	-	-	-	-	-	-	-	-	-	12	0.24	5.71
TTGGC	-	1	-	-	-	-	-	-	-	-	-	-	-	-	-	-	-	-	-	-	-	-	-	-	-	-	-	-	1	0.02	0.48
TTGGT	-	4	-	-	-	-	-	-	-	-	-	-	-	-	-	-	-	-	-	-	-	-	-	-	-	-	-	-	4	0.08	1.90
TTTAA	-	3	-	-	-	-	-	-	-	-	-	-	-	-	-	-	-	-	-	-	-	-	-	-	-	-	-	-	3	0.06	1.43
TTTAG	-	-	10	-	-	-	-	-	-	-	-	-	-	-	-	-	-	-	-	-	-	-	-	-	-	-	-	-	10	0.20	4.76

Supplementary Table S2. Frequencies of different repeat motifs in EST-SSRs from *Capparis spinosa* leaf transcriptome.

Repeats	4	5	6	7	8	9	10	11	12	13	14	15	16	17	18	19	20	21	22	23	24	25	26	27	28	29	30	31	Total	Total (%)	Within motifs (%)
TTTAT	-	1	-	-	-	-	-	-	-	-	-	-	-	-	-	-	-	-	-	-	-	-	-	-	-	-	-	-	1	0.02	0.48
TTTCT	-	-	-	12	-	-	-	-	-	-	-	-	-	-	-	-	-	-	-	-	-	-	-	-	-	-	-	-	12	0.24	5.71
TTTGG	-	-	1	-	-	-	-	-	-	-	-	-	-	-	-	-	-	-	-	-	-	-	-	-	-	-	-	-	1	0.02	0.48
TTTTA	-	2	7	-	-	-	-	-	-	-	-	-	-	-	-	-	-	-	-	-	-	-	-	-	-	-	-	-	9	0.18	4.29
TTTTC	-	3	3	-	-	-	-	-	-	-	-	-	-	-	-	-	-	-	-	-	-	-	-	-	-	-	-	-	6	0.12	2.86
TTTTG	-	6	-	-	-	-	-	-	-	-	-	-	-	-	-	-	-	-	-	-	-	-	-	-	-	-	-	-	6	0.12	2.86
**Tot. Pentanucleotide**																													**210**	**4.20**	
AAAAAC	1	-	-	-	-	-	-	-	-	-	-	-	-	-	-	-	-	-	-	-	-	-	-	-	-	-	-	-	1	0.02	0.09
AAAAAG	11	-	-	-	-	-	-	-	-	-	-	-	-	-	-	-	-	-	-	-	-	-	-	-	-	-	-	-	11	0.22	0.99
AAAAAT	1	-	-	-	-	-	-	-	-	-	-	-	-	-	-	-	-	-	-	-	-	-	-	-	-	-	-	-	1	0.02	0.09
AAAAGA	2	-	-	-	-	-	-	-	-	-	-	-	-	-	-	-	-	-	-	-	-	-	-	-	-	-	-	-	2	0.04	0.18
AAAAGC	9	-	-	-	-	-	-	-	-	-	-	-	-	-	-	-	-	-	-	-	-	-	-	-	-	-	-	-	9	0.18	0.81
AAAATA	-	2	-	-	-	-	-	-	-	-	-	-	-	-	-	-	-	-	-	-	-	-	-	-	-	-	-	-	2	0.04	0.18
AAAGAA	2	-	-	-	-	-	-	-	-	-	-	-	-	-	-	-	-	-	-	-	-	-	-	-	-	-	-	-	2	0.04	0.18
AAATAA	2	-	-	-	-	-	-	-	-	-	-	-	-	-	-	-	-	-	-	-	-	-	-	-	-	-	-	-	2	0.04	0.18
AAATTC	1	-	-	-	-	-	-	-	-	-	-	-	-	-	-	-	-	-	-	-	-	-	-	-	-	-	-	-	1	0.02	0.09
AACAGA	14	-	-	-	-	-	-	-	-	-	-	-	-	-	-	-	-	-	-	-	-	-	-	-	-	-	-	-	14	0.28	1.26
AACAGC	1	1	-	-	-	-	-	-	-	-	-	-	-	-	-	-	-	-	-	-	-	-	-	-	-	-	-	-	2	0.04	0.18
AACCAA	6	-	-	-	-	-	-	-	-	-	-	-	-	-	-	-	-	-	-	-	-	-	-	-	-	-	-	-	6	0.12	0.54
AACCCG	1	-	-	-	-	-	-	-	-	-	-	-	-	-	-	-	-	-	-	-	-	-	-	-	-	-	-	-	1	0.02	0.09
AACCCT	5	-	-	-	-	-	-	-	-	-	-	-	-	-	-	-	-	-	-	-	-	-	-	-	-	-	-	-	5	0.10	0.45
AACTGA	1	-	-	-	-	-	-	-	-	-	-	-	-	-	-	-	-	-	-	-	-	-	-	-	-	-	-	-	1	0.02	0.09
AACTGC	1	-	-	-	-	-	-	-	-	-	-	-	-	-	-	-	-	-	-	-	-	-	-	-	-	-	-	-	1	0.02	0.09
AAGAAA	1	-	-	-	-	-	-	-	-	-	-	-	-	-	-	-	-	-	-	-	-	-	-	-	-	-	-	-	1	0.02	0.09
AAGAAC	7	-	-	-	-	-	-	-	-	-	-	-	-	-	-	-	-	-	-	-	-	-	-	-	-	-	-	-	7	0.14	0.63
AAGACG	2	1	-	-	-	-	-	-	-	-	-	-	-	-	-	-	-	-	-	-	-	-	-	-	-	-	-	-	3	0.06	0.27
AAGAGC	4	-	-	-	-	-	-	-	-	-	-	-	-	-	-	-	-	-	-	-	-	-	-	-	-	-	-	-	4	0.08	0.36
AAGCAA	1	-	-	-	-	-	-	-	-	-	-	-	-	-	-	-	-	-	-	-	-	-	-	-	-	-	-	-	1	0.02	0.09
AAGGAC	1	-	-	-	-	-	-	-	-	-	-	-	-	-	-	-	-	-	-	-	-	-	-	-	-	-	-	-	1	0.02	0.09
AATCCA	1	-	-	-	-	-	-	-	-	-	-	-	-	-	-	-	-	-	-	-	-	-	-	-	-	-	-	-	1	0.02	0.09
AATGGC	-	-	1	-	-	-	-	-	-	-	-	-	-	-	-	-	-	-	-	-	-	-	-	-	-	-	-	-	1	0.02	0.09
AATTTG	1	-	-	-	-	-	-	-	-	-	-	-	-	-	-	-	-	-	-	-	-	-	-	-	-	-	-	-	1	0.02	0.09
ACAAGA	1	-	-	-	-	-	-	-	-	-	-	-	-	-	-	-	-	-	-	-	-	-	-	-	-	-	-	-	1	0.02	0.09
ACACAG	1	-	-	-	-	-	-	-	-	-	-	-	-	-	-	-	-	-	-	-	-	-	-	-	-	-	-	-	1	0.02	0.09
ACAGCC	1	-	-	-	-	-	-	-	-	-	-	-	-	-	-	-	-	-	-	-	-	-	-	-	-	-	-	-	1	0.02	0.09
ACATAT	2	1	-	-	-	-	-	-	-	-	-	-	-	-	-	-	-	-	-	-	-	-	-	-	-	-	-	-	3	0.06	0.27
ACATCA	2	-	-	-	-	-	-	-	-	-	-	-	-	-	-	-	-	-	-	-	-	-	-	-	-	-	-	-	2	0.04	0.18
ACCCGA	-	-	1	1	-	-	-	-	-	-	-	-	-	-	-	-	-	-	-	-	-	-	-	-	-	-	-	-	2	0.04	0.18
ACCCTA	2	-	-	-	-	-	-	-	-	-	-	-	-	-	-	-	-	-	-	-	-	-	-	-	-	-	-	-	2	0.04	0.18
ACGATC	3	-	-	-	-	-	-	-	-	-	-	-	-	-	-	-	-	-	-	-	-	-	-	-	-	-	-	-	3	0.06	0.27
ACGCCT	-	-	-	-	-	1	-	-	-	-	-	-	-	-	-	-	-	-	-	-	-	-	-	-	-	-	-	-	1	0.02	0.09
ACTCCG	1	-	-	-	-	-	-	-	-	-	-	-	-	-	-	-	-	-	-	-	-	-	-	-	-	-	-	-	1	0.02	0.09
ACTGAT	-	-	1	-	-	-	-	-	-	-	-	-	-	-	-	-	-	-	-	-	-	-	-	-	-	-	-	-	1	0.02	0.09
ACTGGA	1	-	-	-	-	-	-	-	-	-	-	-	-	-	-	-	-	-	-	-	-	-	-	-	-	-	-	-	1	0.02	0.09
AGAAAA	3	-	-	-	-	-	-	-	-	-	-	-	-	-	-	-	-	-	-	-	-	-	-	-	-	-	-	-	3	0.06	0.27
AGAACA	-	-	-	-	1	-	-	-	-	-	-	-	-	-	-	-	-	-	-	-	-	-	-	-	-	-	-	-	1	0.02	0.09
AGAAGC	1	9	-	-	-	-	-	-	-	-	-	-	-	-	-	-	-	-	-	-	-	-	-	-	-	-	-	-	10	0.20	0.90
AGAAGG	4	-	-	-	-	-	-	-	-	-	-	-	-	-	-	-	-	-	-	-	-	-	-	-	-	-	-	-	4	0.08	0.36
AGAGAC	1	-	-	-	-	-	-	-	-	-	-	-	-	-	-	-	-	-	-	-	-	-	-	-	-	-	-	-	1	0.02	0.09
AGAGCA	10	-	-	-	-	-	-	-	-	-	-	-	-	-	-	-	-	-	-	-	-	-	-	-	-	-	-	-	10	0.20	0.90
AGAGTA	4	-	-	-	-	-	-	-	-	-	-	-	-	-	-	-	-	-	-	-	-	-	-	-	-	-	-	-	4	0.08	0.36
AGATCG	2	-	-	-	-	-	-	-	-	-	-	-	-	-	-	-	-	-	-	-	-	-	-	-	-	-	-	-	2	0.04	0.18
AGATGA	-	2	-	-	-	-	-	-	-	-	-	-	-	-	-	-	-	-	-	-	-	-	-	-	-	-	-	-	2	0.04	0.18
AGATGC	1	-	-	-	-	-	-	-	-	-	-	-	-	-	-	-	-	-	-	-	-	-	-	-	-	-	-	-	1	0.02	0.09
AGATTG	6	-	-	-	-	-	-	-	-	-	-	-	-	-	-	-	-	-	-	-	-	-	-	-	-	-	-	-	6	0.12	0.54
AGCAAG	2	-	-	-	-	-	-	-	-	-	-	-	-	-	-	-	-	-	-	-	-	-	-	-	-	-	-	-	2	0.04	0.18
AGCAGG	4	2	-	-	-	-	-	-	-	-	-	-	-	-	-	-	-	-	-	-	-	-	-	-	-	-	-	-	6	0.12	0.54
AGCATC	2	-	-	-	-	-	-	-	-	-	-	-	-	-	-	-	-	-	-	-	-	-	-	-	-	-	-	-	2	0.04	0.18
AGCCAA	1	-	-	-	-	-	-	-	-	-	-	-	-	-	-	-	-	-	-	-	-	-	-	-	-	-	-	-	1	0.02	0.09
AGCTCC	1	-	-	-	-	-	-	-	-	-	-	-	-	-	-	-	-	-	-	-	-	-	-	-	-	-	-	-	1	0.02	0.09
AGGAAG	4	-	-	-	-	-	-	-	-	-	-	-	-	-	-	-	-	-	-	-	-	-	-	-	-	-	-	-	4	0.08	0.36
AGGGAG	4	-	-	-	-	-	-	-	-	-	-	-	-	-	-	-	-	-	-	-	-	-	-	-	-	-	-	-	4	0.08	0.36
AGGTTC	1	-	-	-	-	-	-	-	-	-	-	-	-	-	-	-	-	-	-	-	-	-	-	-	-	-	-	-	1	0.02	0.09
AGTCGC	-	2	-	-	-	-	-	-	-	-	-	-	-	-	-	-	-	-	-	-	-	-	-	-	-	-	-	-	2	0.04	0.18
ATAATT	1	-	-	-	-	-	-	-	-	-	-	-	-	-	-	-	-	-	-	-	-	-	-	-	-	-	-	-	1	0.02	0.09
ATACAT	-	-	-	1	-	-	-	-	-	-	-	-	-	-	-	-	-	-	-	-	-	-	-	-	-	-	-	-	1	0.02	0.09
ATAGGA	1	-	-	-	-	-	-	-	-	-	-	-	-	-	-	-	-	-	-	-	-	-	-	-	-	-	-	-	1	0.02	0.09
ATATAC	-	2	-	1	-	-	-	-	-	-	-	-	-	-	-	-	-	-	-	-	-	-	-	-	-	-	-	-	3	0.06	0.27
ATATGG	3	-	-	-	-	-	-	-	-	-	-	-	-	-	-	-	-	-	-	-	-	-	-	-	-	-	-	-	3	0.06	0.27
ATATGT	2	-	-	1	-	-	-	-	-	-	-	-	-	-	-	-	-	-	-	-	-	-	-	-	-	-	-	-	3	0.06	0.27
ATCAAC	1	-	-	-	-	-	-	-	-	-	-	-	-	-	-	-	-	-	-	-	-	-	-	-	-	-	-	-	1	0.02	0.09
ATCACC	-	-	3	-	-	-	-	-	-	-	-	-	-	-	-	-	-	-	-	-	-	-	-	-	-	-	-	-	3	0.06	0.27
ATCAGC	-	-	-	2	-	-	-	-	-	-	-	-	-	-	-	-	-	-	-	-	-	-	-	-	-	-	-	-	2	0.04	0.18
ATCATA	-	-	-	2	-	-	-	-	-	-	-	-	-	-	-	-	-	-	-	-	-	-	-	-	-	-	-	-	2	0.04	0.18
ATCCAG	-	-	1	-	-	-	-	-	-	-	-	-	-	-	-	-	-	-	-	-	-	-	-	-	-	-	-	-	1	0.02	0.09
ATCGTC	1	-	-	-	-	-	-	-	-	-	-	-	-	-	-	-	-	-	-	-	-	-	-	-	-	-	-	-	1	0.02	0.09
ATCTCC	1	-	-	-	-	-	-	-	-	-	-	-	-	-	-	-	-	-	-	-	-	-	-	-	-	-	-	-	1	0.02	0.09
ATCTTT	-	1	-	-	-	-	-	-	-	-	-	-	-	-	-	-	-	-	-	-	-	-	-	-	-	-	-	-	1	0.02	0.09
ATGAAG	1	-	-	-	-	-	-	-	-	-	-	-	-	-	-	-	-	-	-	-	-	-	-	-	-	-	-	-	1	0.02	0.09
ATGACC	-	-	-	-	2	-	-	-	-	-	-	-	-	-	-	-	-	-	-	-	-	-	-	-	-	-	-	-	2	0.04	0.18
ATGAGA	-	-	-	-	1	-	-	-	-	-	-	-	-	-	-	-	-	-	-	-	-	-	-	-	-	-	-	-	1	0.02	0.09
ATGCAA	1	-	-	-	-	-	-	-	-	-	-	-	-	-	-	-	-	-	-	-	-	-	-	-	-	-	-	-	1	0.02	0.09
ATGGGA	6	-	-	-	-	-	-	-	-	-	-	-	-	-	-	-	-	-	-	-	-	-	-	-	-	-	-	-	6	0.12	0.54
ATGGTG	1	1	-	-	-	-	-	-	-	-	-	-	-	-	-	-	-	-	-	-	-	-	-	-	-	-	-	-	2	0.04	0.18
ATGTGC	4	-	-	-	-	-	-	-	-	-	-	-	-	-	-	-	-	-	-	-	-	-	-	-	-	-	-	-	4	0.08	0.36
ATTATG	2	-	-	-	-	-	-	-	-	-	-	-	-	-	-	-	-	-	-	-	-	-	-	-	-	-	-	-	2	0.04	0.18
ATTGCG	1	-	-	-	-	-	-	-	-	-	-	-	-	-	-	-	-	-	-	-	-	-	-	-	-	-	-	-	1	0.02	0.09
ATTTAA	-	5	-	-	-	-	-	-	-	-	-	-	-	-	-	-	-	-	-	-	-	-	-	-	-	-	-	-	5	0.10	0.45
ATTTAT	1	-	-	-	-	-	-	-	-	-	-	-	-	-	-	-	-	-	-	-	-	-	-	-	-	-	-	-	1	0.02	0.09
ATTTCG	1	-	-	-	-	-	-	-	-	-	-	-	-	-	-	-	-	-	-	-	-	-	-	-	-	-	-	-	1	0.02	0.09
ATTTGG	2	-	-	-	-	-	-	-	-	-	-	-	-	-	-	-	-	-	-	-	-	-	-	-	-	-	-	-	2	0.04	0.18
ATTTTT	5	-	-	-	-	-	-	-	-	-	-	-	-	-	-	-	-	-	-	-	-	-	-	-	-	-	-	-	5	0.10	0.45
CAAAAC	1	-	-	-	-	-	-	-	-	-	-	-	-	-	-	-	-	-	-	-	-	-	-	-	-	-	-	-	1	0.02	0.09
CAAACG	-	1	-	-	1	-	-	-	-	-	-	-	-	-	-	-	-	-	-	-	-	-	-	-	-	-	-	-	2	0.04	0.18
CAACAT	-	-	-	1	-	-	-	-	-	-	-	-	-	-	-	-	-	-	-	-	-	-	-	-	-	-	-	-	1	0.02	0.09
CAAGAA	2	-	-	-	-	-	-	-	-	-	-	-	-	-	-	-	-	-	-	-	-	-	-	-	-	-	-	-	2	0.04	0.18
CAAGCA	3	-	-	-	-	-	-	-	-	-	-	-	-	-	-	-	-	-	-	-	-	-	-	-	-	-	-	-	3	0.06	0.27
CAATGG	2	7	-	-	-	-	-	-	-	-	-	-	-	-	-	-	-	-	-	-	-	-	-	-	-	-	-	-	9	0.18	0.81
CACAGC	1	-	-	-	-	-	-	-	-	-	-	-	-	-	-	-	-	-	-	-	-	-	-	-	-	-	-	-	1	0.02	0.09
CACCAA	-	-	-	1	-	-	-	-	-	-	-	-	-	-	-	-	-	-	-	-	-	-	-	-	-	-	-	-	1	0.02	0.09
CACCTC	3	-	-	-	-	-	-	-	-	-	-	-	-	-	-	-	-	-	-	-	-	-	-	-	-	-	-	-	3	0.06	0.27
CACTGC	-	-	-	1	-	-	-	-	-	-	-	-	-	-	-	-	-	-	-	-	-	-	-	-	-	-	-	-	1	0.02	0.09

Supplementary Table S2. Frequencies of different repeat motifs in EST-SSRs from *Capparis spinosa* leaf transcriptome.

Repeats	4	5	6	7	8	9	10	11	12	13	14	15	16	17	18	19	20	21	22	23	24	25	26	27	28	29	30	31	Total	Total (%)	Within motifs (%)
CAGAGG	1	-	-	-	-	-	-	-	-	-	-	-	-	-	-	-	-	-	-	-	-	-	-	-	-	-	-	-	1	0.02	0.09
CAGCAA	4	-	1	-	-	-	-	-	-	-	-	-	-	-	-	-	-	-	-	-	-	-	-	-	-	-	-	-	5	0.10	0.45
CAGCAC	1	-	-	-	-	-	-	-	-	-	-	-	-	-	-	-	-	-	-	-	-	-	-	-	-	-	-	-	1	0.02	0.09
CAGCAT	-	-	-	3	-	-	-	-	-	-	-	-	-	-	-	-	-	-	-	-	-	-	-	-	-	-	-	-	3	0.06	0.27
CAGCCA	1	-	-	-	-	1	-	-	-	-	-	-	-	-	-	-	-	-	-	-	-	-	-	-	-	-	-	-	2	0.04	0.18
CAGCCG	6	-	-	-	-	-	-	-	-	-	-	-	-	-	-	-	-	-	-	-	-	-	-	-	-	-	-	-	6	0.12	0.54
CAGCCT	1	-	-	-	-	-	-	-	-	-	-	-	-	-	-	-	-	-	-	-	-	-	-	-	-	-	-	-	1	0.02	0.09
CAGCGG	3	-	-	-	-	-	-	-	-	-	-	-	-	-	-	-	-	-	-	-	-	-	-	-	-	-	-	-	3	0.06	0.27
CAGGCG	13	-	-	-	-	-	-	-	-	-	-	-	-	-	-	-	-	-	-	-	-	-	-	-	-	-	-	-	13	0.26	1.17
CAGGGT	1	-	-	-	-	-	-	-	-	-	-	-	-	-	-	-	-	-	-	-	-	-	-	-	-	-	-	-	1	0.02	0.09
CAGTTT	7	1	-	-	-	-	-	-	-	-	-	-	-	-	-	-	-	-	-	-	-	-	-	-	-	-	-	-	8	0.16	0.72
CATATA	3	-	-	-	-	-	-	-	-	-	-	-	-	-	-	-	-	-	-	-	-	-	-	-	-	-	-	-	3	0.06	0.27
CATATC	1	-	-	-	-	-	-	-	-	-	-	-	-	-	-	-	-	-	-	-	-	-	-	-	-	-	-	-	1	0.02	0.09
CATCAC	1	-	1	-	-	-	-	-	-	-	-	-	-	-	-	-	-	-	-	-	-	-	-	-	-	-	-	-	2	0.04	0.18
CATCAG	3	-	-	-	-	-	-	-	-	-	-	-	-	-	-	-	-	-	-	-	-	-	-	-	-	-	-	-	3	0.06	0.27
CATCTG	6	-	-	-	-	-	-	-	-	-	-	-	-	-	-	-	-	-	-	-	-	-	-	-	-	-	-	-	6	0.12	0.54
CATGAA	6	-	-	-	-	-	-	-	-	-	-	-	-	-	-	-	-	-	-	-	-	-	-	-	-	-	-	-	6	0.12	0.54
CATTGC	2	-	-	-	-	-	-	-	-	-	-	-	-	-	-	-	-	-	-	-	-	-	-	-	-	-	-	-	2	0.04	0.18
CATTTT	5	-	-	-	-	-	-	-	-	-	-	-	-	-	-	-	-	-	-	-	-	-	-	-	-	-	-	-	5	0.10	0.45
CCAAAG	-	1	-	-	-	-	-	-	-	-	-	-	-	-	-	-	-	-	-	-	-	-	-	-	-	-	-	-	1	0.02	0.09
CCAACC	7	-	-	-	-	-	-	-	-	-	-	-	-	-	-	-	-	-	-	-	-	-	-	-	-	-	-	-	7	0.14	0.63
CCAAGT	-	-	1	-	-	-	-	-	-	-	-	-	-	-	-	-	-	-	-	-	-	-	-	-	-	-	-	-	1	0.02	0.09
CCACAG	-	-	3	-	-	-	-	-	-	-	-	-	-	-	-	-	-	-	-	-	-	-	-	-	-	-	-	-	3	0.06	0.27
CCACGC	-	-	-	1	-	-	-	-	-	-	-	-	-	-	-	-	-	-	-	-	-	-	-	-	-	-	-	-	1	0.02	0.09
CCACTC	-	-	-	1	-	-	-	-	-	-	-	-	-	-	-	-	-	-	-	-	-	-	-	-	-	-	-	-	1	0.02	0.09
CCAGAT	-	-	2	-	-	-	-	-	-	-	-	-	-	-	-	-	-	-	-	-	-	-	-	-	-	-	-	-	2	0.04	0.18
CCAGGA	1	-	-	-	-	-	-	-	-	-	-	-	-	-	-	-	-	-	-	-	-	-	-	-	-	-	-	-	1	0.02	0.09
CCATCA	1	-	-	-	-	-	-	-	-	-	-	-	-	-	-	-	-	-	-	-	-	-	-	-	-	-	-	-	1	0.02	0.09
CCCAGA	1	1	-	-	-	-	-	1	-	-	-	-	-	-	-	-	-	-	-	-	-	-	-	-	-	-	-	-	3	0.06	0.27
CCCTAA	-	-	3	-	-	-	-	-	-	-	-	-	-	-	-	-	-	-	-	-	-	-	-	-	-	-	-	-	3	0.06	0.27
CCCTCT	3	-	-	-	-	-	-	-	-	-	-	-	-	-	-	-	-	-	-	-	-	-	-	-	-	-	-	-	3	0.06	0.27
CCGACG	-	-	-	-	-	1	-	-	-	-	-	-	-	-	-	-	-	-	-	-	-	-	-	-	-	-	-	-	1	0.02	0.09
CCGCAA	-	1	2	-	-	-	-	-	-	-	-	-	-	-	-	-	-	-	-	-	-	-	-	-	-	-	-	-	3	0.06	0.27
CCGCAG	6	-	-	-	-	-	-	-	-	-	-	-	-	-	-	-	-	-	-	-	-	-	-	-	-	-	-	-	6	0.12	0.54
CCGCCA	4	-	-	-	-	-	-	-	-	-	-	-	-	-	-	-	-	-	-	-	-	-	-	-	-	-	-	-	4	0.08	0.36
CCGCCT	1	-	-	-	-	-	-	-	-	-	-	-	-	-	-	-	-	-	-	-	-	-	-	-	-	-	-	-	1	0.02	0.09
CCGGCA	-	-	1	-	-	-	-	-	-	-	-	-	-	-	-	-	-	-	-	-	-	-	-	-	-	-	-	-	1	0.02	0.09
CCGTCA	1	-	-	-	-	-	-	-	-	-	-	-	-	-	-	-	-	-	-	-	-	-	-	-	-	-	-	-	1	0.02	0.09
CCTAAC	1	-	-	-	-	-	-	-	-	-	-	-	-	-	-	-	-	-	-	-	-	-	-	-	-	-	-	-	1	0.02	0.09
CCTAAG	6	-	-	-	-	-	-	-	-	-	-	-	-	-	-	-	-	-	-	-	-	-	-	-	-	-	-	-	6	0.12	0.54
CCTCAA	5	-	-	-	-	-	-	-	-	-	-	-	-	-	-	-	-	-	-	-	-	-	-	-	-	-	-	-	5	0.10	0.45
CCTCAC	1	-	-	-	-	-	-	-	-	-	-	-	-	-	-	-	-	-	-	-	-	-	-	-	-	-	-	-	1	0.02	0.09
CCTCCA	8	1	-	-	6	-	-	-	-	-	-	-	-	-	-	-	-	-	-	-	-	-	-	-	-	-	-	-	15	0.30	1.35
CCTCCG	1	-	2	-	-	-	-	-	-	-	-	-	-	-	-	-	-	-	-	-	-	-	-	-	-	-	-	-	3	0.06	0.27
CCTCTC	1	-	1	-	-	-	-	-	-	-	-	-	-	-	-	-	-	-	-	-	-	-	-	-	-	-	-	-	2	0.04	0.18
CCTCTT	2	-	-	-	-	-	-	-	-	-	-	-	-	-	-	-	-	-	-	-	-	-	-	-	-	-	-	-	2	0.04	0.18
CCTGCT	-	-	3	-	2	-	-	-	-	-	-	-	-	-	-	-	-	-	-	-	-	-	-	-	-	-	-	-	5	0.10	0.45
CCTTCT	4	-	-	-	-	-	-	-	-	-	-	-	-	-	-	-	-	-	-	-	-	-	-	-	-	-	-	-	4	0.08	0.36
CCTTTA	-	-	-	-	-	-	-	-	-	1	-	-	-	-	-	-	-	-	-	-	-	-	-	-	-	-	-	-	1	0.02	0.09
CGAAAT	1	-	-	-	-	-	-	-	-	-	-	-	-	-	-	-	-	-	-	-	-	-	-	-	-	-	-	-	1	0.02	0.09
CGAACC	1	-	-	-	-	-	-	-	-	-	-	-	-	-	-	-	-	-	-	-	-	-	-	-	-	-	-	-	1	0.02	0.09
CGACCT	-	1	1	-	-	-	-	-	-	-	-	-	-	-	-	-	-	-	-	-	-	-	-	-	-	-	-	-	2	0.04	0.18
CGACGG	1	-	-	-	-	-	-	-	-	-	-	-	-	-	-	-	-	-	-	-	-	-	-	-	-	-	-	-	1	0.02	0.09
CGATGA	1	-	-	-	-	-	-	-	-	-	-	-	-	-	-	-	-	-	-	-	-	-	-	-	-	-	-	-	1	0.02	0.09
CGCACG	1	-	-	-	-	-	-	-	-	-	-	-	-	-	-	-	-	-	-	-	-	-	-	-	-	-	-	-	1	0.02	0.09
CGCAGC	1	-	-	-	-	-	-	-	-	-	-	-	-	-	-	-	-	-	-	-	-	-	-	-	-	-	-	-	1	0.02	0.09
CGCAGT	-	-	-	2	-	-	-	-	-	-	-	-	-	-	-	-	-	-	-	-	-	-	-	-	-	-	-	-	2	0.04	0.18
CGCCCG	6	-	-	-	-	-	-	-	-	-	-	-	-	-	-	-	-	-	-	-	-	-	-	-	-	-	-	-	6	0.12	0.54
CGGAGG	1	-	-	-	-	-	-	-	-	-	-	-	-	-	-	-	-	-	-	-	-	-	-	-	-	-	-	-	1	0.02	0.09
CGGCCA	-	2	-	-	-	-	-	-	-	-	-	-	-	-	-	-	-	-	-	-	-	-	-	-	-	-	-	-	2	0.04	0.18
CGGCGA	8	-	-	-	-	-	-	-	-	-	-	-	-	-	-	-	-	-	-	-	-	-	-	-	-	-	-	-	8	0.16	0.72
CGGCGT	1	-	-	-	-	-	-	-	-	-	-	-	-	-	-	-	-	-	-	-	-	-	-	-	-	-	-	-	1	0.02	0.09
CGGGAT	2	-	-	-	-	-	-	-	-	-	-	-	-	-	-	-	-	-	-	-	-	-	-	-	-	-	-	-	2	0.04	0.18
CGGGCT	1	-	-	-	-	-	-	-	-	-	-	-	-	-	-	-	-	-	-	-	-	-	-	-	-	-	-	-	1	0.02	0.09
CGGTGC	1	-	-	-	-	-	-	-	-	-	-	-	-	-	-	-	-	-	-	-	-	-	-	-	-	-	-	-	1	0.02	0.09
CGGTTT	1	-	-	-	-	-	-	-	-	-	-	-	-	-	-	-	-	-	-	-	-	-	-	-	-	-	-	-	1	0.02	0.09
CGTCAA	-	-	-	-	-	2	-	-	-	-	-	-	-	-	-	-	-	-	-	-	-	-	-	-	-	-	-	-	2	0.04	0.18
CGTCAT	-	-	-	-	-	2	-	-	-	-	-	-	-	-	-	-	-	-	-	-	-	-	-	-	-	-	-	-	2	0.04	0.18
CGTCTC	5	-	-	-	-	-	-	-	-	-	-	-	-	-	-	-	-	-	-	-	-	-	-	-	-	-	-	-	5	0.10	0.45
CGTCTT	2	-	-	-	-	-	-	-	-	-	-	-	-	-	-	-	-	-	-	-	-	-	-	-	-	-	-	-	2	0.04	0.18
CGTTTT	13	-	-	-	-	-	-	-	-	-	-	-	-	-	-	-	-	-	-	-	-	-	-	-	-	-	-	-	13	0.26	1.17
CTAACC	1	-	-	-	-	-	-	-	-	-	-	-	-	-	-	-	-	-	-	-	-	-	-	-	-	-	-	-	1	0.02	0.09
CTATAT	1	-	-	-	-	-	-	-	-	-	-	-	-	-	-	-	-	-	-	-	-	-	-	-	-	-	-	-	1	0.02	0.09
CTATCT	-	2	-	-	-	-	-	-	-	-	-	-	-	-	-	-	-	-	-	-	-	-	-	-	-	-	-	-	2	0.04	0.18
CTCAGC	-	-	2	-	-	-	-	-	-	-	-	-	-	-	-	-	-	-	-	-	-	-	-	-	-	-	-	-	2	0.04	0.18
CTCCAC	-	2	-	-	-	-	-	-	-	-	-	-	-	-	-	-	-	-	-	-	-	-	-	-	-	-	-	-	2	0.04	0.18
CTCCGC	3	5	-	-	-	-	-	-	-	-	-	-	-	-	-	-	-	-	-	-	-	-	-	-	-	-	-	-	8	0.16	0.72
CTCTCC	4	3	-	-	-	-	-	-	-	-	-	-	-	-	-	-	-	-	-	-	-	-	-	-	-	-	-	-	7	0.14	0.63
CTCTGC	1	-	-	-	-	-	-	-	-	-	-	-	-	-	-	-	-	-	-	-	-	-	-	-	-	-	-	-	1	0.02	0.09
CTCTGT	2	-	-	-	-	-	-	-	-	-	-	-	-	-	-	-	-	-	-	-	-	-	-	-	-	-	-	-	2	0.04	0.18
CTGATG	7	-	-	-	-	-	-	-	-	-	-	-	-	-	-	-	-	-	-	-	-	-	-	-	-	-	-	-	7	0.14	0.63
CTGATT	1	-	-	-	-	-	-	-	-	-	-	-	-	-	-	-	-	-	-	-	-	-	-	-	-	-	-	-	1	0.02	0.09
CTGCAA	-	-	-	1	-	-	-	-	-	-	-	-	-	-	-	-	-	-	-	-	-	-	-	-	-	-	-	-	1	0.02	0.09
CTGCCA	-	1	-	-	-	-	-	-	-	-	-	-	-	-	-	-	-	-	-	-	-	-	-	-	-	-	-	-	1	0.02	0.09
CTGCGT	1	-	-	-	-	-	-	-	-	-	-	-	-	-	-	-	-	-	-	-	-	-	-	-	-	-	-	-	1	0.02	0.09
CTGCTA	4	-	-	-	-	-	-	-	-	-	-	-	-	-	-	-	-	-	-	-	-	-	-	-	-	-	-	-	4	0.08	0.36
CTGCTT	1	-	-	-	-	-	-	-	-	-	-	-	-	-	-	-	-	-	-	-	-	-	-	-	-	-	-	-	1	0.02	0.09
CTGGAT	2	-	-	-	-	-	-	-	-	-	-	-	-	-	-	-	-	-	-	-	-	-	-	-	-	-	-	-	2	0.04	0.18
CTGTCT	-	-	5	-	-	-	-	-	-	-	-	-	-	-	-	-	-	-	-	-	-	-	-	-	-	-	-	-	5	0.10	0.45
CTGTGG	7	-	-	-	-	-	-	-	-	-	-	-	-	-	-	-	-	-	-	-	-	-	-	-	-	-	-	-	7	0.14	0.63
CTTCAT	1	-	-	-	-	-	-	-	-	-	-	-	-	-	-	-	-	-	-	-	-	-	-	-	-	-	-	-	1	0.02	0.09
CTTCCG	-	-	4	-	-	-	-	-	-	-	-	-	-	-	-	-	-	-	-	-	-	-	-	-	-	-	-	-	4	0.08	0.36
CTTCGT	1	-	-	-	-	-	-	-	-	-	-	-	-	-	-	-	-	-	-	-	-	-	-	-	-	-	-	-	1	0.02	0.09
CTTCTC	4	1	-	-	-	-	-	-	-	-	-	-	-	-	-	-	-	-	-	-	-	-	-	-	-	-	-	-	5	0.10	0.45
CTTCTG	-	1	3	-	-	-	-	-	-	-	-	-	-	-	-	-	-	-	-	-	-	-	-	-	-	-	-	-	4	0.08	0.36
CTTGCT	1	-	-	-	-	-	-	-	-	-	-	-	-	-	-	-	-	-	-	-	-	-	-	-	-	-	-	-	1	0.02	0.09
CTTTCA	1	-	-	-	-	-	-	-	-	-	-	-	-	-	-	-	-	-	-	-	-	-	-	-	-	-	-	-	1	0.02	0.09
CTTTTC	1	-	-	-	-	-	-	-	-	-	-	-	-	-	-	-	-	-	-	-	-	-	-	-	-	-	-	-	1	0.02	0.09
CTTTTT	7	1	-	-	-	-	-	-	-	-	-	-	-	-	-	-	-	-	-	-	-	-	-	-	-	-	-	-	8	0.16	0.72
GAAAAC	1	-	-	-	-	-	-	-	-	-	-	-	-	-	-	-	-	-	-	-	-	-	-	-	-	-	-	-	1	0.02	0.09
GAAAGT	1	-	-	-	-	-	-	-	-	-	-	-	-	-	-	-	-	-	-	-	-	-	-	-	-	-	-	-	1	0.02	0.09
GAACAA	2	-	-	-	-	-	-	-	-	-	-	-	-	-	-	-	-	-	-	-	-	-	-	-	-	-	-	-	2	0.04	0.18

Supplementary Table S2. Frequencies of different repeat motifs in EST-SSRs from *Capparis spinosa* leaf transcriptome.

Repeats	4	5	6	7	8	9	10	11	12	13	14	15	16	17	18	19	20	21	22	23	24	25	26	27	28	29	30	31	Total	Total (%)	Within motifs (%)
GAACCT	-	-	-	1	-	-	-	-	-	-	-	-	-	-	-	-	-	-	-	-	-	-	-	-	-	-	-	-	1	0.02	0.09
GAACTG	2	-	-	-	-	-	-	-	-	-	-	-	-	-	-	-	-	-	-	-	-	-	-	-	-	-	-	-	2	0.04	0.18
GAAGAC	1	-	2	-	-	-	-	-	-	-	-	-	-	-	-	-	-	-	-	-	-	-	-	-	-	-	-	-	3	0.06	0.27
GAAGAG	4	2	-	-	-	-	-	-	-	-	-	-	-	-	-	-	-	-	-	-	-	-	-	-	-	-	-	-	6	0.12	0.54
GAAGAT	3	-	-	-	-	-	-	-	-	-	-	-	-	-	-	-	-	-	-	-	-	-	-	-	-	-	-	-	3	0.06	0.27
GAAGCA	20	-	-	1	-	-	-	-	-	-	-	-	-	-	-	-	-	-	-	-	-	-	-	-	-	-	-	-	21	0.42	1.88
GAATCC	1	-	-	-	-	-	-	-	-	-	-	-	-	-	-	-	-	-	-	-	-	-	-	-	-	-	-	-	1	0.02	0.09
GACAAG	1	-	-	-	-	-	-	-	-	-	-	-	-	-	-	-	-	-	-	-	-	-	-	-	-	-	-	-	1	0.02	0.09
GACCGA	5	-	-	-	-	-	-	-	-	-	-	-	-	-	-	-	-	-	-	-	-	-	-	-	-	-	-	-	5	0.10	0.45
GACGAA	-	3	6	-	-	-	-	-	-	-	-	-	-	-	-	-	-	-	-	-	-	-	-	-	-	-	-	-	9	0.18	0.81
GACGAG	-	-	5	-	-	-	-	-	-	-	-	-	-	-	-	-	-	-	-	-	-	-	-	-	-	-	-	-	5	0.10	0.45
GACGAT	7	-	2	2	-	-	-	-	-	-	-	-	-	-	-	-	-	-	-	-	-	-	-	-	-	-	-	-	11	0.22	0.99
GACGCC	-	-	-	-	3	-	-	-	-	-	-	-	-	-	-	-	-	-	-	-	-	-	-	-	-	-	-	-	3	0.06	0.27
GACGGT	2	-	-	-	-	-	-	-	-	-	-	-	-	-	-	-	-	-	-	-	-	-	-	-	-	-	-	-	2	0.04	0.18
GACTGC	-	-	-	-	-	-	1	-	-	-	-	-	-	-	-	-	-	-	-	-	-	-	-	-	-	-	-	-	1	0.02	0.09
GAGAAG	11	-	-	-	-	-	-	-	-	-	-	-	-	-	-	-	-	-	-	-	-	-	-	-	-	-	-	-	11	0.22	0.99
GAGAGG	1	4	-	-	-	-	-	-	-	-	-	-	-	-	-	-	-	-	-	-	-	-	-	-	-	-	-	-	5	0.10	0.45
GAGCCA	1	-	-	-	-	-	-	-	-	-	-	-	-	-	-	-	-	-	-	-	-	-	-	-	-	-	-	-	1	0.02	0.09
GAGGAA	1	-	7	-	-	-	-	-	-	-	-	-	-	-	-	-	-	-	-	-	-	-	-	-	-	-	-	-	8	0.16	0.72
GAGTTA	3	-	-	-	-	-	-	-	-	-	-	-	-	-	-	-	-	-	-	-	-	-	-	-	-	-	-	-	3	0.06	0.27
GATAAC	4	-	-	-	-	-	-	-	-	-	-	-	-	-	-	-	-	-	-	-	-	-	-	-	-	-	-	-	4	0.08	0.36
GATCCA	2	-	-	-	-	-	-	-	-	-	-	-	-	-	-	-	-	-	-	-	-	-	-	-	-	-	-	-	2	0.04	0.18
GATCTC	1	-	-	-	-	-	-	-	-	-	-	-	-	-	-	-	-	-	-	-	-	-	-	-	-	-	-	-	1	0.02	0.09
GATGAA	3	-	-	3	1	-	-	-	-	-	-	1	-	-	-	-	-	-	-	-	-	-	-	-	-	-	-	-	8	0.16	0.72
GATGAG	10	-	-	-	-	-	-	-	-	-	-	-	-	-	-	-	-	-	-	-	-	-	-	-	-	-	-	-	10	0.20	0.90
GATGCA	-	2	-	-	-	-	-	-	-	-	-	-	-	-	-	-	-	-	-	-	-	-	-	-	-	-	-	-	2	0.04	0.18
GATGGA	2	2	-	-	-	1	-	-	-	-	-	-	-	-	-	-	-	-	-	-	-	-	-	-	-	-	-	-	5	0.10	0.45
GATGGC	2	-	-	-	-	-	-	-	-	-	-	-	-	-	-	-	-	-	-	-	-	-	-	-	-	-	-	-	2	0.04	0.18
GATGGT	2	-	-	-	-	-	-	-	-	-	-	-	-	-	-	-	-	-	-	-	-	-	-	-	-	-	-	-	2	0.04	0.18
GATTAT	1	-	-	-	-	-	-	-	-	-	-	-	-	-	-	-	-	-	-	-	-	-	-	-	-	-	-	-	1	0.02	0.09
GATTCA	1	-	-	-	-	-	-	-	-	-	-	-	-	-	-	-	-	-	-	-	-	-	-	-	-	-	-	-	1	0.02	0.09
GATTGC	1	-	-	-	-	-	-	-	-	-	-	-	-	-	-	-	-	-	-	-	-	-	-	-	-	-	-	-	1	0.02	0.09
GATTTC	1	-	-	-	-	-	-	-	-	-	-	-	-	-	-	-	-	-	-	-	-	-	-	-	-	-	-	-	1	0.02	0.09
GATTTG	-	5	-	-	-	-	-	-	-	-	-	-	-	-	-	-	-	-	-	-	-	-	-	-	-	-	-	-	5	0.10	0.45
GATTTT	1	-	-	-	-	-	-	-	-	-	-	-	-	-	-	-	-	-	-	-	-	-	-	-	-	-	-	-	1	0.02	0.09
GCAAAC	-	1	-	-	-	-	-	-	-	-	-	-	-	-	-	-	-	-	-	-	-	-	-	-	-	-	-	-	1	0.02	0.09
GCAACA	4	-	-	-	-	-	-	-	-	-	-	-	-	-	-	-	-	-	-	-	-	-	-	-	-	-	-	-	4	0.08	0.36
GCAGCC	2	-	-	-	-	-	-	-	-	-	-	-	-	-	-	-	-	-	-	-	-	-	-	-	-	-	-	-	2	0.04	0.18
GCAGCG	2	-	-	-	-	-	-	-	-	-	-	-	-	-	-	-	-	-	-	-	-	-	-	-	-	-	-	-	2	0.04	0.18
GCAGGA	1	-	-	-	-	-	-	-	-	-	-	-	-	-	-	-	-	-	-	-	-	-	-	-	-	-	-	-	1	0.02	0.09
GCAGTG	-	-	-	1	-	-	-	-	-	-	-	-	-	-	-	-	-	-	-	-	-	-	-	-	-	-	-	-	1	0.02	0.09
GCATCA	1	-	-	-	-	-	-	-	-	-	-	-	-	-	-	-	-	-	-	-	-	-	-	-	-	-	-	-	1	0.02	0.09
GCCACA	3	-	-	-	-	-	1	-	-	-	-	-	-	-	-	-	-	-	-	-	-	-	-	-	-	-	-	-	4	0.08	0.36
GCCACC	1	-	-	-	-	-	-	-	-	-	-	-	-	-	-	-	-	-	-	-	-	-	-	-	-	-	-	-	1	0.02	0.09
GCCATT	2	-	-	-	-	-	-	-	-	-	-	-	-	-	-	-	-	-	-	-	-	-	-	-	-	-	-	-	2	0.04	0.18
GCCGCA	1	-	-	-	-	-	-	-	-	-	-	-	-	-	-	-	-	-	-	-	-	-	-	-	-	-	-	-	1	0.02	0.09
GCCTCA	-	-	1	-	-	-	-	-	-	-	-	-	-	-	-	-	-	-	-	-	-	-	-	-	-	-	-	-	1	0.02	0.09
GCCTCC	-	2	-	2	-	-	-	-	-	-	-	-	-	-	-	-	-	-	-	-	-	-	-	-	-	-	-	-	4	0.08	0.36
GCCTCT	1	-	-	-	-	-	-	-	-	-	-	-	-	-	-	-	-	-	-	-	-	-	-	-	-	-	-	-	1	0.02	0.09
GCCTGA	-	-	1	-	-	-	-	-	-	-	-	-	-	-	-	-	-	-	-	-	-	-	-	-	-	-	-	-	1	0.02	0.09
GCCTGG	-	-	2	-	-	-	-	-	-	-	-	-	-	-	-	-	-	-	-	-	-	-	-	-	-	-	-	-	2	0.04	0.18
GCGGTG	3	-	-	-	-	-	-	-	-	-	-	-	-	-	-	-	-	-	-	-	-	-	-	-	-	-	-	-	3	0.06	0.27
GCTCAT	2	-	-	-	-	-	-	-	-	-	-	-	-	-	-	-	-	-	-	-	-	-	-	-	-	-	-	-	2	0.04	0.18
GCTCCA	1	-	-	-	-	-	-	-	-	-	-	-	-	-	-	-	-	-	-	-	-	-	-	-	-	-	-	-	1	0.02	0.09
GCTGCG	2	-	-	-	-	-	-	-	-	-	-	-	-	-	-	-	-	-	-	-	-	-	-	-	-	-	-	-	2	0.04	0.18
GCTTCC	-	-	2	-	-	3	-	-	-	-	-	-	-	-	-	-	-	-	-	-	-	-	-	-	-	-	-	-	5	0.10	0.45
GCTTCT	4	-	-	-	-	-	-	-	-	-	-	-	-	-	-	-	-	-	-	-	-	-	-	-	-	-	-	-	4	0.08	0.36
GGAACT	1	-	-	-	-	-	-	-	-	-	-	-	-	-	-	-	-	-	-	-	-	-	-	-	-	-	-	-	1	0.02	0.09
GGAAGA	2	-	1	-	-	-	-	-	-	-	-	-	-	-	-	-	-	-	-	-	-	-	-	-	-	-	-	-	3	0.06	0.27
GGACCC	1	-	-	-	-	-	-	-	-	-	-	-	-	-	-	-	-	-	-	-	-	-	-	-	-	-	-	-	1	0.02	0.09
GGAGAA	2	-	-	-	-	-	-	-	-	-	-	-	-	-	-	-	-	-	-	-	-	-	-	-	-	-	-	-	2	0.04	0.18
GGAGAG	9	-	-	-	-	-	-	-	-	-	-	-	-	-	-	-	-	-	-	-	-	-	-	-	-	-	-	-	9	0.18	0.81
GGAGAT	1	-	-	-	-	-	-	-	-	-	-	-	-	-	-	-	-	-	-	-	-	-	-	-	-	-	-	-	1	0.02	0.09
GGATGA	-	-	-	1	-	-	-	-	-	-	-	-	-	-	-	-	-	-	-	-	-	-	-	-	-	-	-	-	1	0.02	0.09
GGCAAG	7	-	-	-	-	-	-	-	-	-	-	-	-	-	-	-	-	-	-	-	-	-	-	-	-	-	-	-	7	0.14	0.63
GGCACC	2	-	-	-	-	-	-	-	-	-	-	-	-	-	-	-	-	-	-	-	-	-	-	-	-	-	-	-	2	0.04	0.18
GGCAGA	1	-	-	-	-	-	-	-	-	-	-	-	-	-	-	-	-	-	-	-	-	-	-	-	-	-	-	-	1	0.02	0.09
GGCGAT	-	3	-	-	-	-	-	-	-	-	-	-	-	-	-	-	-	-	-	-	-	-	-	-	-	-	-	-	3	0.06	0.27
GGCGGT	5	-	-	-	-	-	-	-	-	-	-	-	-	-	-	-	-	-	-	-	-	-	-	-	-	-	-	-	5	0.10	0.45
GGCTCA	2	-	-	-	-	-	-	-	-	-	-	-	-	-	-	-	-	-	-	-	-	-	-	-	-	-	-	-	2	0.04	0.18
GGCTGT	6	-	-	-	-	-	-	-	-	-	-	-	-	-	-	-	-	-	-	-	-	-	-	-	-	-	-	-	6	0.12	0.54
GGCTTC	1	-	-	-	-	-	-	-	-	-	-	-	-	-	-	-	-	-	-	-	-	-	-	-	-	-	-	-	1	0.02	0.09
GGGAGA	3	-	-	-	-	-	-	-	-	-	-	-	-	-	-	-	-	-	-	-	-	-	-	-	-	-	-	-	3	0.06	0.27
GGGATG	5	-	-	-	-	-	-	-	-	-	-	-	-	-	-	-	-	-	-	-	-	-	-	-	-	-	-	-	5	0.10	0.45
GGGTTA	2	-	-	-	-	-	-	-	-	-	-	-	-	-	-	-	-	-	-	-	-	-	-	-	-	-	-	-	2	0.04	0.18
GGTAAA	-	-	-	-	-	-	-	-	-	1	-	-	-	-	-	-	-	-	-	-	-	-	-	-	-	-	-	-	1	0.02	0.09
GGTATG	-	1	-	-	-	-	-	-	-	-	-	-	-	-	-	-	-	-	-	-	-	-	-	-	-	-	-	-	1	0.02	0.09
GGTCAT	1	-	-	-	-	-	-	-	-	-	-	-	-	-	-	-	-	-	-	-	-	-	-	-	-	-	-	-	1	0.02	0.09
GGTGCT	3	-	-	-	-	-	-	-	-	-	-	-	-	-	-	-	-	-	-	-	-	-	-	-	-	-	-	-	3	0.06	0.27
GGTTCA	3	-	-	-	-	-	-	-	-	-	-	-	-	-	-	-	-	-	-	-	-	-	-	-	-	-	-	-	3	0.06	0.27
GGTTTT	5	-	-	-	-	-	-	-	-	-	-	-	-	-	-	-	-	-	-	-	-	-	-	-	-	-	-	-	5	0.10	0.45
GTATAT	1	1	-	1	3	-	-	-	-	-	-	-	-	-	-	-	-	-	-	-	-	-	-	-	-	-	-	-	6	0.12	0.54
GTCATG	1	-	-	-	-	-	-	-	-	-	-	-	-	-	-	-	-	-	-	-	-	-	-	-	-	-	-	-	1	0.02	0.09
GTCGTG	1	-	-	-	-	-	-	-	-	-	-	-	-	-	-	-	-	-	-	-	-	-	-	-	-	-	-	-	1	0.02	0.09
GTCTCT	1	-	-	-	-	-	-	-	-	-	-	-	-	-	-	-	-	-	-	-	-	-	-	-	-	-	-	-	1	0.02	0.09
GTCTGT	1	-	-	-	-	-	-	-	-	-	-	-	-	-	-	-	-	-	-	-	-	-	-	-	-	-	-	-	1	0.02	0.09
GTCTTG	1	-	-	-	-	-	-	-	-	-	-	-	-	-	-	-	-	-	-	-	-	-	-	-	-	-	-	-	1	0.02	0.09
GTGAAA	1	-	-	-	-	-	-	-	-	-	-	-	-	-	-	-	-	-	-	-	-	-	-	-	-	-	-	-	1	0.02	0.09
GTGATG	1	-	-	-	-	-	-	-	-	-	-	-	-	-	-	-	-	-	-	-	-	-	-	-	-	-	-	-	1	0.02	0.09
GTGGCA	1	-	-	-	-	-	-	-	-	-	-	-	-	-	-	-	-	-	-	-	-	-	-	-	-	-	-	-	1	0.02	0.09
GTGGTC	4	-	-	-	-	-	-	-	-	-	-	-	-	-	-	-	-	-	-	-	-	-	-	-	-	-	-	-	4	0.08	0.36
GTGTGA	1	-	-	-	-	-	-	-	-	-	-	-	-	-	-	-	-	-	-	-	-	-	-	-	-	-	-	-	1	0.02	0.09
GTTAGG	2	-	-	-	-	-	-	-	-	-	-	-	-	-	-	-	-	-	-	-	-	-	-	-	-	-	-	-	2	0.04	0.18
GTTCAG	3	-	-	-	-	-	-	-	-	-	-	-	-	-	-	-	-	-	-	-	-	-	-	-	-	-	-	-	3	0.06	0.27
GTTCCA	2	-	-	-	-	-	-	-	-	-	-	-	-	-	-	-	-	-	-	-	-	-	-	-	-	-	-	-	2	0.04	0.18
GTTCGG	2	-	-	-	-	-	-	-	-	-	-	-	-	-	-	-	-	-	-	-	-	-	-	-	-	-	-	-	2	0.04	0.18
GTTCTT	-	-	1	-	-	-	-	-	-	-	-	-	-	-	-	-	-	-	-	-	-	-	-	-	-	-	-	-	1	0.02	0.09
GTTGCA	1	-	-	-	-	-	-	-	-	-	-	-	-	-	-	-	-	-	-	-	-	-	-	-	-	-	-	-	1	0.02	0.09
GTTGCG	-	-	-	1	-	-	-	-	-	-	-	-	-	-	-	-	-	-	-	-	-	-	-	-	-	-	-	-	1	0.02	0.09
GTTGGA	3	-	-	-	-	-	-	-	-	-	-	-	-	-	-	-	-	-	-	-	-	-	-	-	-	-	-	-	3	0.06	0.27
GTTGGT	1	-	-	-	-	-	-	-	-	-	-	-	-	-	-	-	-	-	-	-	-	-	-	-	-	-	-	-	1	0.02	0.09
GTTTCA	2	-	-	-	-	-	-	-	-	-	-	-	-	-	-	-	-	-	-	-	-	-	-	-	-	-	-	-	2	0.04	0.18

Supplementary Table S2. Frequencies of different repeat motifs in EST-SSRs from *Capparis spinosa* leaf transcriptome.

Repeats	4	5	6	7	8	9	10	11	12	13	14	15	16	17	18	19	20	21	22	23	24	25	26	27	28	29	30	31	Total	Total (%)	Within motifs (%)
GTTTCC	-	2	-	-	-	-	-	-	-	-	-	-	-	-	-	-	-	-	-	-	-	-	-	-	-	-	-	-	2	0.04	0.18
GTTTGG	2	-	-	-	-	-	-	-	-	-	-	-	-	-	-	-	-	-	-	-	-	-	-	-	-	-	-	-	2	0.04	0.18
GTTTGT	-	-	1	-	-	-	-	-	-	-	-	-	-	-	-	-	-	-	-	-	-	-	-	-	-	-	-	-	1	0.02	0.09
GTTTTT	1	-	-	-	-	-	-	-	-	-	-	-	-	-	-	-	-	-	-	-	-	-	-	-	-	-	-	-	1	0.02	0.09
TAATAT	1	-	-	-	-	-	-	-	-	-	-	-	-	-	-	-	-	-	-	-	-	-	-	-	-	-	-	-	1	0.02	0.09
TAATTT	8	-	-	-	-	-	-	-	-	-	-	-	-	-	-	-	-	-	-	-	-	-	-	-	-	-	-	-	8	0.16	0.72
TACACG	-	2	-	-	-	-	-	-	-	-	-	-	-	-	-	-	-	-	-	-	-	-	-	-	-	-	-	-	2	0.04	0.18
TACATA	4	-	-	-	-	-	-	-	-	-	-	-	-	-	-	-	-	-	-	-	-	-	-	-	-	-	-	-	4	0.08	0.36
TACCAA	-	1	-	-	-	-	-	-	-	-	-	-	-	-	-	-	-	-	-	-	-	-	-	-	-	-	-	-	1	0.02	0.09
TACCTC	1	-	-	-	-	-	-	-	-	-	-	-	-	-	-	-	-	-	-	-	-	-	-	-	-	-	-	-	1	0.02	0.09
TATTGG	1	-	-	-	-	-	-	-	-	-	-	-	-	-	-	-	-	-	-	-	-	-	-	-	-	-	-	-	1	0.02	0.09
TATTTT	9	-	-	-	-	-	-	-	-	-	-	-	-	-	-	-	-	-	-	-	-	-	-	-	-	-	-	-	9	0.18	0.81
TCAACC	2	-	-	-	-	-	-	-	-	-	-	-	-	-	-	-	-	-	-	-	-	-	-	-	-	-	-	-	2	0.04	0.18
TCACAG	1	-	-	-	-	-	-	-	-	-	-	-	-	-	-	-	-	-	-	-	-	-	-	-	-	-	-	-	1	0.02	0.09
TCACCA	17	-	-	-	-	-	-	-	-	-	-	-	-	-	-	-	-	-	-	-	-	-	-	-	-	-	-	-	17	0.34	1.52
TCAGCA	-	-	-	-	-	1	-	-	-	-	-	-	-	-	-	-	-	-	-	-	-	-	-	-	-	-	-	-	1	0.02	0.09
TCAGCT	-	4	-	-	-	-	-	-	-	-	-	-	-	-	-	-	-	-	-	-	-	-	-	-	-	-	-	-	4	0.08	0.36
TCATCG	1	-	1	-	-	-	-	-	-	-	-	-	-	-	-	-	-	-	-	-	-	-	-	-	-	-	-	-	2	0.04	0.18
TCATCT	1	-	2	-	-	-	-	-	-	-	-	-	-	-	-	-	-	-	-	-	-	-	-	-	-	-	-	-	3	0.06	0.27
TCCACA	10	-	-	-	-	-	-	-	-	-	-	-	-	-	-	-	-	-	-	-	-	-	-	-	-	-	-	-	10	0.20	0.90
TCCATT	1	-	-	-	-	-	-	-	-	-	-	-	-	-	-	-	-	-	-	-	-	-	-	-	-	-	-	-	1	0.02	0.09
TCCCGA	1	-	-	-	-	-	-	-	-	-	-	-	-	-	-	-	-	-	-	-	-	-	-	-	-	-	-	-	1	0.02	0.09
TCCGGA	2	-	-	-	-	-	-	-	-	-	-	-	-	-	-	-	-	-	-	-	-	-	-	-	-	-	-	-	2	0.04	0.18
TCCTCA	1	-	-	-	-	-	-	-	-	-	-	-	-	-	-	-	-	-	-	-	-	-	-	-	-	-	-	-	1	0.02	0.09
TCCTCT	6	-	-	-	-	-	-	-	-	-	-	-	-	-	-	-	-	-	-	-	-	-	-	-	-	-	-	-	6	0.12	0.54
TCGAGA	3	-	-	-	-	-	-	-	-	-	-	-	-	-	-	-	-	-	-	-	-	-	-	-	-	-	-	-	3	0.06	0.27
TCGATT	1	-	-	-	-	-	-	-	-	-	-	-	-	-	-	-	-	-	-	-	-	-	-	-	-	-	-	-	1	0.02	0.09
TCGCAG	1	-	-	-	-	-	-	-	-	-	-	-	-	-	-	-	-	-	-	-	-	-	-	-	-	-	-	-	1	0.02	0.09
TCGTCA	1	-	-	-	-	-	-	-	-	-	-	-	-	-	-	-	-	-	-	-	-	-	-	-	-	-	-	-	1	0.02	0.09
TCGTCT	5	2	-	-	-	-	-	-	-	-	-	-	-	-	-	-	-	-	-	-	-	-	-	-	-	-	-	-	7	0.14	0.63
TCTACA	4	-	-	-	-	-	-	-	-	-	-	-	-	-	-	-	-	-	-	-	-	-	-	-	-	-	-	-	4	0.08	0.36
TCTCCC	1	-	-	-	-	-	-	-	-	-	-	-	-	-	-	-	-	-	-	-	-	-	-	-	-	-	-	-	1	0.02	0.09
TCTCGC	1	-	-	-	-	-	-	-	-	-	-	-	-	-	-	-	-	-	-	-	-	-	-	-	-	-	-	-	1	0.02	0.09
TCTCTA	6	2	-	-	-	-	-	-	-	-	-	-	-	-	-	-	-	-	-	-	-	-	-	-	-	-	-	-	8	0.16	0.72
TCTCTG	1	-	-	-	-	-	-	-	-	-	-	-	-	-	-	-	-	-	-	-	-	-	-	-	-	-	-	-	1	0.02	0.09
TCTGCC	1	-	-	-	-	-	-	-	-	-	-	-	-	-	-	-	-	-	-	-	-	-	-	-	-	-	-	-	1	0.02	0.09
TCTGTT	6	-	-	-	-	-	-	-	-	-	-	-	-	-	-	-	-	-	-	-	-	-	-	-	-	-	-	-	6	0.12	0.54
TCTTCA	2	1	-	-	-	-	-	-	-	-	-	-	-	-	-	-	-	-	-	-	-	-	-	-	-	-	-	-	3	0.06	0.27
TCTTCC	3	-	-	-	-	-	-	-	-	-	-	-	-	-	-	-	-	-	-	-	-	-	-	-	-	-	-	-	3	0.06	0.27
TCTTCG	9	7	-	-	-	-	-	-	-	-	-	-	-	-	-	-	-	-	-	-	-	-	-	-	-	-	-	-	16	0.32	1.43
TCTTTT	-	2	-	-	-	-	-	-	-	-	-	-	-	-	-	-	-	-	-	-	-	-	-	-	-	-	-	-	2	0.04	0.18
TGAAAT	1	-	-	-	-	-	-	-	-	-	-	-	-	-	-	-	-	-	-	-	-	-	-	-	-	-	-	-	1	0.02	0.09
TGACGA	-	1	2	-	-	-	-	-	-	-	-	-	-	-	-	-	-	-	-	-	-	-	-	-	-	-	-	-	3	0.06	0.27
TGAGCG	1	-	-	-	-	-	-	-	-	-	-	-	-	-	-	-	-	-	-	-	-	-	-	-	-	-	-	-	1	0.02	0.09
TGAGGA	-	-	1	-	-	-	3	-	-	-	-	-	-	-	-	-	-	-	-	-	-	-	-	-	-	-	-	-	4	0.08	0.36
TGATGG	2	-	-	-	-	3	-	-	-	-	-	-	-	-	-	-	-	-	-	-	-	-	-	-	-	-	-	-	5	0.10	0.45
TGATTT	2	-	-	-	-	-	-	-	-	-	-	-	-	-	-	-	-	-	-	-	-	-	-	-	-	-	-	-	2	0.04	0.18
TGCAGA	1	-	-	-	-	-	-	-	-	-	-	-	-	-	-	-	-	-	-	-	-	-	-	-	-	-	-	-	1	0.02	0.09
TGCGTG	1	-	-	-	-	-	-	-	-	-	-	-	-	-	-	-	-	-	-	-	-	-	-	-	-	-	-	-	1	0.02	0.09
TGCGTT	1	2	-	-	1	-	-	-	-	-	-	-	-	-	-	-	-	-	-	-	-	-	-	-	-	-	-	-	4	0.08	0.36
TGCTCC	2	-	-	4	-	-	-	-	-	-	-	-	-	-	-	-	-	-	-	-	-	-	-	-	-	-	-	-	6	0.12	0.54
TGCTTC	2	-	-	-	-	-	-	-	-	-	-	-	-	-	-	-	-	-	-	-	-	-	-	-	-	-	-	-	2	0.04	0.18
TGGAGA	1	-	-	-	-	-	-	-	-	-	-	-	-	-	-	-	-	-	-	-	-	-	-	-	-	-	-	-	1	0.02	0.09
TGGAGC	5	-	-	-	-	-	-	-	-	-	-	-	-	-	-	-	-	-	-	-	-	-	-	-	-	-	-	-	5	0.10	0.45
TGGCAG	3	-	-	-	-	-	-	-	-	-	-	-	-	-	-	-	-	-	-	-	-	-	-	-	-	-	-	-	3	0.06	0.27
TGGCGG	1	-	-	-	-	-	-	-	-	-	-	-	-	-	-	-	-	-	-	-	-	-	-	-	-	-	-	-	1	0.02	0.09
TGGGCT	2	-	-	-	-	-	-	-	-	-	-	-	-	-	-	-	-	-	-	-	-	-	-	-	-	-	-	-	2	0.04	0.18
TGGTCA	1	-	-	-	-	-	-	-	-	-	-	-	-	-	-	-	-	-	-	-	-	-	-	-	-	-	-	-	1	0.02	0.09
TGGTGA	1	-	-	-	-	-	-	-	-	-	-	-	-	-	-	-	-	-	-	-	-	-	-	-	-	-	-	-	1	0.02	0.09
TGGTTC	4	-	-	-	-	-	-	-	-	-	-	-	-	-	-	-	-	-	-	-	-	-	-	-	-	-	-	-	4	0.08	0.36
TGTATG	3	-	-	-	-	-	-	-	-	-	-	-	-	-	-	-	-	-	-	-	-	-	-	-	-	-	-	-	3	0.06	0.27
TGTCTT	1	-	-	-	-	-	-	-	-	-	-	-	-	-	-	-	-	-	-	-	-	-	-	-	-	-	-	-	1	0.02	0.09
TGTGAG	6	-	-	-	-	-	-	-	-	-	-	-	-	-	-	-	-	-	-	-	-	-	-	-	-	-	-	-	6	0.12	0.54
TGTGAT	1	-	-	-	-	-	-	-	-	-	-	-	-	-	-	-	-	-	-	-	-	-	-	-	-	-	-	-	1	0.02	0.09
TGTGCG	1	-	-	-	-	-	-	-	-	-	-	-	-	-	-	-	-	-	-	-	-	-	-	-	-	-	-	-	1	0.02	0.09
TGTGGA	1	-	-	-	-	-	-	-	-	-	-	-	-	-	-	-	-	-	-	-	-	-	-	-	-	-	-	-	1	0.02	0.09
TGTGTA	1	-	-	-	-	-	-	-	-	-	-	-	-	-	-	-	-	-	-	-	-	-	-	-	-	-	-	-	1	0.02	0.09
TGTTCT	-	-	-	3	-	-	-	-	-	-	-	-	-	-	-	-	-	-	-	-	-	-	-	-	-	-	-	-	3	0.06	0.27
TTAAAA	2	-	-	-	-	-	-	-	-	-	-	-	-	-	-	-	-	-	-	-	-	-	-	-	-	-	-	-	2	0.04	0.18
TTATTG	1	-	-	-	-	-	-	-	-	-	-	-	-	-	-	-	-	-	-	-	-	-	-	-	-	-	-	-	1	0.02	0.09
TTATTT	1	-	-	-	-	-	-	-	-	-	-	-	-	-	-	-	-	-	-	-	-	-	-	-	-	-	-	-	1	0.02	0.09
TTCGAT	1	-	-	-	-	-	-	-	-	-	-	-	-	-	-	-	-	-	-	-	-	-	-	-	-	-	-	-	1	0.02	0.09
TTCGGA	1	-	-	-	-	-	-	-	-	-	-	-	-	-	-	-	-	-	-	-	-	-	-	-	-	-	-	-	1	0.02	0.09
TTCTCC	1	-	-	-	-	-	-	-	-	-	-	-	-	-	-	-	-	-	-	-	-	-	-	-	-	-	-	-	1	0.02	0.09
TTCTGC	-	-	-	1	-	-	-	-	-	-	-	-	-	-	-	-	-	-	-	-	-	-	-	-	-	-	-	-	1	0.02	0.09
TTCTTG	2	-	-	-	-	-	-	-	-	-	-	-	-	-	-	-	-	-	-	-	-	-	-	-	-	-	-	-	2	0.04	0.18
TTCTTT	4	-	-	-	-	-	-	-	-	-	-	-	-	-	-	-	-	-	-	-	-	-	-	-	-	-	-	-	4	0.08	0.36
TTGAGA	1	-	-	-	-	-	-	-	-	-	-	-	-	-	-	-	-	-	-	-	-	-	-	-	-	-	-	-	1	0.02	0.09
TTGAGC	1	-	-	-	-	-	-	-	-	-	-	-	-	-	-	-	-	-	-	-	-	-	-	-	-	-	-	-	1	0.02	0.09
TTGATT	-	1	-	-	-	-	-	-	-	-	-	-	-	-	-	-	-	-	-	-	-	-	-	-	-	-	-	-	1	0.02	0.09
TTGCGT	1	-	-	-	-	-	-	-	-	-	-	-	-	-	-	-	-	-	-	-	-	-	-	-	-	-	-	-	1	0.02	0.09
TTGTTT	1	-	-	-	-	-	-	-	-	-	-	-	-	-	-	-	-	-	-	-	-	-	-	-	-	-	-	-	1	0.02	0.09
TTTAGT	1	-	-	-	-	-	-	-	-	-	-	-	-	-	-	-	-	-	-	-	-	-	-	-	-	-	-	-	1	0.02	0.09
TTTATT	1	-	-	-	-	-	-	-	-	-	-	-	-	-	-	-	-	-	-	-	-	-	-	-	-	-	-	-	1	0.02	0.09
TTTCTT	4	-	-	-	-	-	-	-	-	-	-	-	-	-	-	-	-	-	-	-	-	-	-	-	-	-	-	-	4	0.08	0.36
TTTGCG	-	5	-	-	-	-	-	-	-	-	-	-	-	-	-	-	-	-	-	-	-	-	-	-	-	-	-	-	5	0.10	0.45
TTTGGT	-	1	-	-	-	-	-	-	-	-	-	-	-	-	-	-	-	-	-	-	-	-	-	-	-	-	-	-	1	0.02	0.09
TTTGTT	3	-	-	-	-	-	-	-	-	-	-	-	-	-	-	-	-	-	-	-	-	-	-	-	-	-	-	-	3	0.06	0.27
TTTTAT	1	-	-	-	-	-	-	-	-	-	-	-	-	-	-	-	-	-	-	-	-	-	-	-	-	-	-	-	1	0.02	0.09
TTTTCT	2	-	-	-	-	-	-	-	-	-	-	-	-	-	-	-	-	-	-	-	-	-	-	-	-	-	-	-	2	0.04	0.18
TTTTGA	-	-	3	-	-	-	-	-	-	-	-	-	-	-	-	-	-	-	-	-	-	-	-	-	-	-	-	-	3	0.06	0.27
TTTTGT	6	-	-	-	-	-	-	-	-	-	-	-	-	-	-	-	-	-	-	-	-	-	-	-	-	-	-	-	6	0.12	0.54
TTTTTA	3	-	-	-	-	-	-	-	-	-	-	-	-	-	-	-	-	-	-	-	-	-	-	-	-	-	-	-	3	0.06	0.27
TTTTTC	1	-	-	-	-	-	-	-	-	-	-	-	-	-	-	-	-	-	-	-	-	-	-	-	-	-	-	-	1	0.02	0.09
TTTTTG	4	-	-	-	-	-	-	-	-	-	-	-	-	-	-	-	-	-	-	-	-	-	-	-	-	-	-	-	4	0.08	0.36
**Tot. Exanucleotide**																													**1,115**	**22.26**	
**Tot. EST-SSR**																													**5,009**		

Supplementary Table S3. List of 150 primer pairs designed. Fifty EST-SSR selected and tested in the present study are reported in italic. Polymorphic markers (14) with expected size are in bold. Monomorphic markers in our collection with expected size (13) are underlined.

Primer	Motif	Forward primer (5'-3')	Reverse primer (5'-3')	Expected size (bp)
ESTcapp1	*(TCC)7*	*GTTTCCGCCTCCTCATACG*	*GCGCAAGCGAGTGAAGATAG*	*111*
ESTcapp2	*(AAAT)5*	*GGAGATTTACGTGACTGTTGATG*	*ATATCCCACCAGCTTTGACC*	*235*
ESTcapp3	*(GTTT)5*	*GGTGATATTGATGCCGTGTC*	*CCCAAGCGTCCAAACTCTC*	*303*
ESTcapp4	*(ATC)7*	*TGGCTTCGTATTTCCTGAGAG*	*TCGTGGATGGCGTAGATTTC*	*185*
ESTcapp5	***(TC)20***	***GGTGAGTGGTATGAGCACAAAG***	***GATCGTCATCTGAGGGAAAG***	***101***
ESTcapp6	*(TC)20*	*TCGCTTCTCTTCTGCTTCTTC*	*GACTATCCCAAGGACTGATGG*	*150*
ESTcapp7	*(TC)7*	*TCAATGGAGTCATGGGTAGTTC*	*GGACTGATGGCAGAGAATCG*	*168*
ESTcapp8	***(AGA)7***	***GGCCACTAGATCACTTGTTAGTC***	***TTGCCCTTTCTTGCATGTC***	***120***
ESTcapp9	*(AG)20*	*ACGTACCAGCCACCTAAACC*	*ACCAAACCAATTCTACTGCTTC*	*147*
ESTcapp10	***(AG)20***	***TGATGGTGAAGGAAGAGAAGC***	***TCCCATATTCCAAACAAACC***	***141***
ESTcapp11	***(CGGTGC)4***	***AGTTCCGGCGTATTTAGTGC***	***GCCACAATGAGTTTCCAAGC***	***153***
ESTcapp12	*(AAG)7*	*ATGGATGTTGAGGTCAGTGTAAG*	*ACTTGCGCGACGAATCAC*	*397*
ESTcapp13	*(AG)20*	*CATGCTGCGTTCGAGATTG*	*TGGAGGAGAGAGAGCGAGAG*	*195*
ESTcapp14	***(CAAA)5***	***TGAGACAACAGCACAAATAACC***	***GACACCGGACGAAACACTTC***	***153***
ESTcapp15	*(TC)20*	*GGAGTCGGAGTGGATTACG*	*CACCGTCGAAATTGTTATTCG*	*166*
ESTcapp16	*(GAAAAG)4*	*GAAATGTGGCGATAATGACG*	*TTTGGTGTGCAGAAGTTTGTC*	*203*
ESTcapp17	*(TC)20*	*TTCTCCACCATTCTCCAACC*	*CAGATTCCCAACTGGTCTCC*	*191*
ESTcapp18	***(TC)20***	***GGTTAAGTTTGGGAGCTGGAG***	***CCCATGAGAAGAGTGGCTTG***	***105***
ESTcapp19	*(TTTCT)5*	*GATTCGGTCCATTCTCAAGC*	*TGCAAACAGAACAGATCACG*	*167*
ESTcapp20	***(AG)20***	***CACCTGTCACTCCATTATCATCC***	***CTGGTCGTGATGGGAAAGTC***	***120***
ESTcapp21	***(AAG)7***	***AAGGGTACGGGATGCTTATG***	***GAATGAGCCAGTTTCTGTTGC***	***138***
ESTcapp22	*(AG)20*	*ACCTCCAGATGTTGCAGAATG*	*CCATAGTGTAAGGGCCGATG*	*100*

Supplementary Table S3. List of 150 primer pairs designed. Fifty EST-SSR selected and tested in the present study are reported in italic. Polymorphic markers (14) with expected size are in bold. Monomorphic markers in our collection with expected size (13) are underlined.

Primer	Motif	Forward primer (5'-3')	Reverse primer (5'-3')	Expected size (bp)
ESTcapp23	*(AGAAG)5*	*GACCAACAGTCTAGCCCATTC*	*GTCCGCATTAAGCCAGATG*	*100*
ESTcapp24	*(ATG)7*	*GCCATTCCAAGGCTCATTAC*	*GCTAATCCCAGCTACAACAGG*	*139*
ESTcapp25	*(TGT)7*	*GCATATCCCTTCTTGCATCG*	*CAGTTTGCAATCGACACAGC*	*140*
ESTcapp26	*(AT)20*	*CAAGCAATTAAAGCAGGCAAG*	*CTGGTGGTGGTAGAACAGAAG*	*186*
ESTcapp27	*(TCACA)5*	*TTAAGTTGCTGGCCGAAATAC*	*CTTGACCTGCCCAAACAATC*	*309*
ESTcapp28	*(TC)20*	*TCCATCCACGTACCTTGTCC*	*AACTCCCGCCTCAACTTTC*	*237*
ESTcapp29	*(ACAAA)5*	*TGTTCTCCGACCTCCACTTC*	*TTAATGTTTCGCGGTGTCTG*	*141*
ESTcapp30	*(TGCA)5*	*CGTGACTTGTGGTCGAGATTAC*	*AAATGACACGGAGGATGAAG*	*175*
ESTcapp31	*(TGT)7*	*CAATGTGAGGTGGGAGGATG*	*CCGGTATCGACTGAACCAC*	*115*
ESTcapp32	***(AT)20***	***GGAAGAGTTCTCCATCCCAAG***	***GCTCAATCACACCGATCTGC***	***164***
ESTcapp33	***(CGC)7***	***TGAGATCCTCGTTAGCTTCG***	***ACGTAGTCATGGCGAGACG***	***150***
ESTcapp34	*(TCG)7*	*ACTGGTCTTGCCGACTCCTG*	*AAAGAGGGAAGTGGGTATTTGG*	*251*
ESTcapp35	***(TGT)7***	***ACTGCCGTTGTTGTTCCAAG***	***CCCAGTAGCTGTTCTCTCAAGC***	***114***
ESTcapp36	*(CAACA)5*	*CTCAAAGCACACTGCAAACC*	*AAGACCCTCACGAAACAGAAAG*	*101*
ESTcapp37	***(GCAT)5***	***TGATCTCTCTTCCCATTCATTC***	***AGGCTTCTACGGTTCTGAGG***	***100***
ESTcapp38	*(AT)20*	*AAATGAAGCCCACGCATTAG*	*CCCATCTTAACCATTAGCCAAC*	*254*
ESTcapp39	*(TC)20*	*CCTAGAGGAGAGGCAACAAGG*	*CCGGATCAAACAAACAAACAG*	*118*
ESTcapp40	*(TC)20*	*TTGTTGAATGTTGGTGTGACG*	*TTGACAGAGAGGGAAGAGAGG*	*100*
ESTcapp41	*(TCA)7*	*TGTGCCTGTGGAAGATTGTC*	*TGTACCAAGCAAAGCCCAAG*	*149*
ESTcapp42	*(AAAC)5*	*CAATAGAAACCTCTTCCACACC*	*GATGGAGGGAAATTATGCTG*	*221*
ESTcapp43	*(TTG)7*	*TTCCTCGTTATTGGGCTCTC*	*AAGCAGCCGCATCTATCATC*	*103*
ESTcapp44	*(GCTG)5*	*ACAGCTTGATGTCTCTGAAGAAG*	*ATATTCGGGCACTGAAATCG*	*210*
ESTcapp45	*(GCAT)5*	*ATCTCTCTTCCCATTCATTCG*	*GCTTCTACGGTTCTGAGGAT*	*100*
ESTcapp46	***(AAG)7***	***CGGTACGGGATGCTTATGC***	***ATGAGCCAGTTACTGTTGC***	***130***

Supplementary Table S3. List of 150 primer pairs designed. Fifty EST-SSR selected and tested in the present study are reported in italic. Polymorphic markers (14) with expected size are in bold. Monomorphic markers in our collection with expected size (13) are underlined.

Primer	Motif	Forward primer (5'-3')	Reverse primer (5'-3')	Expected size (bp)
ESTcapp47	*(AAG)7*	*CCGTACGGGATGCTTAGG*	*ATGAGCCAGTCTCTGTTGCTCC*	*110*
ESTcapp48	*(AGAAG)5*	*GACAAACAGTCTAGCATTCC*	*GTAAGCATTAAGCCAGATGC*	*102*
ESTcapp49	***(TGT)7***	***GCTTATCTCTTCTTGCCCATC***	***CAGCTCTTGAATCGAACACAG***	***137***
ESTcapp50	*(AT)20*	*CGCAATTAAGCAGGCAAGC*	*CTGCTATGGTAGAACAGACG*	*190*
ESTcapp51	(GAT)7	GACGGTGGTGAGTGTTCTTTA	TTTCACTATCAACCCCAAAAA	179
ESTcapp52	(TTG)8	CATCGGAAATCTCTTTCCTTC	GATTGAAGGTGTTTGTGCAAG	147
ESTcapp53	(GAA)7	GGTGGGGAATCTTTGTTTCT	CGACACAACTGATTGATACGA	150
ESTcapp54	(TCT)9	GAAAATGATTCCAGCATCTGA	CAGCTGCAATAAACTTGCTAA	150
ESTcapp55	(GAA)18	TGTGTCCTTTAGTGGAGCAAG	ACGACAACAGACGAACAATTC	147
ESTcapp56	(CCTCCG)4	AGGTGGTGAAGACATTCATTG	AAGCTTCATGCAGAATCCAG	161
ESTcapp57	(TTC)8	CAAAGGTGAGAGCTCTTTGTG	CCAGCTGCAATAAACTTGCTA	183
ESTcapp58	(TGGAGC)4	TGTCCATGTGAACAGACAAAA	CTGGAATCCATCCAAAAACA	156
ESTcapp59	(TTATTT)4	AATCTCTGTGGATTTTGTCACC	CGTTTCATCGAACAATTTGAA	151
ESTcapp60	(AGCTCC)4	TGTACGCTTGCTTCAACTTCT	CACCCAAACCATCCTCTTTAC	148
ESTcapp61	(GCTCCA)4	CTTGTGTTCGCTCTCTTCAAC	GAAACCATCCGTTCTTTGACT	135
ESTcapp62	(TTTTAT)4	CACATTTATTCAGCGTCAACC	TCGACTTTCCTGTGATCTTGT	163
ESTcapp63	(TTA)7	TTTCATGATCACCAATTCACC	GCTGAGGGGAAAAGAAAAA	159
ESTcapp64	(AGAG)6	CCGAAAGATCTCTCACAAAAT	CGACTAGGATTTTTCCCGTTA	186
ESTcapp65	(CTA)7	CACGATTTTTCCATTCCAATA	AGAAGAAGAACCACACCATCC	159
ESTcapp66	(GACCA)5	AAAATTCGCCTAAGCAGAAAC	GGAGACGAGAGGAGAAAGAGA	148
ESTcapp67	(GGTCT)6	TAATCAGATAGGCGTTTTGGA	CTGAACAACAAACCGCTAAAC	150
ESTcapp68	(CCAAAG)5	CTCGTTCTCAACCTCCTCTTC	CAAGCTTAAGGGTGTCTCTGG	154
ESTcapp69	(ACAT)7	TGAGCCATTACTTCGATTTTG	CGACAGAAGCGATAAGTTCAC	146
ESTcapp70	(TGCAG)9	AAGGAGGATTTCGTCTTTTTG	ACTTTGCCATAAACACTGCAC	150

Supplementary Table S3. List of 150 primer pairs designed. Fifty EST-SSR selected and tested in the present study are reported in italic. Polymorphic markers (14) with expected size are in bold. Monomorphic markers in our collection with expected size (13) are underlined.

Primer	Motif	Forward primer (5'-3')	Reverse primer (5'-3')	Expected size (bp)
ESTcapp71	(AAG)7	CTTGTCTTCCTCTTCCCTCAG	TTTCTTATCGTCCTTGCCTCT	159
ESTcapp72	(TTC)8	GGGACCTGAAAATAGTTGAGC	CCATTAACCTAAAAGCCCACA	157
ESTcapp73	(GAA)8	ATCCTAGTCATGATGGGGAAA	CCTGTTCTTGATCTCACTCCA	146
ESTcapp74	(GAA)8	ATTAACGGCGGGATTATTAAA	ACGTTCATACTCGATGGACAA	152
ESTcapp75	(CTCT)10	GGAAGTAACGGAAAATCTGGA	GGAGACCAAATTGGAACTAGC	160
ESTcapp76	(ATGT)5	GCACACACAAATACACGCTAT	CCACGTGCACATTCATACATA	156
ESTcapp77	(AAAAGC)4	CATGCTGTAGAAAACCTGGAT	GTGCTGTTGTCCATATCAAGC	151
ESTcapp78	(AAAAGC)4	TTGATCGGTTATATGGCTTTG	AATCTTCTGGTTTGCTTGGTT	157
ESTcapp79	(AAAT)5	TCGTTGTTCCTTTGGATCTATT	ACCTTAAGGGTCCTTTTAGCC	159
ESTcapp80	(TCA)13	CCAAGAAACAGAACCATACCA	GGAGATTCTTGTAGGCAGGTG	167
ESTcapp81	(GCT)7	AATAAACAACCGTCGCTCATC	CACTGGATGCTGAACCTTTTA	151
ESTcapp82	(TGTGAT)4	GATTTTCTCATCCGTTCCCTA	ACAAACCTCGCAAGGAATAAC	125
ESTcapp83	(ACTGGA)4	TTGGTAGCCTTAGCAAAGAAA	GCCACAGGTTCTGCTGAG	145
ESTcapp84	(TCT)8	TTTTGGGGGTTACGTGATAAT	GTTCCGAACGAAGAAGAAGAA	148
ESTcapp85	(AGG)11	ATGATTGCAGACTGGTCAGAG	GCTCAGCTATAAAGGCATGAA	145
ESTcapp86	(AAAG)5	GTTGATCACATACAGCCCTTG	TAGAATGCGGAATCTGGATAA	138
ESTcapp87	(GGACCC)4	TCTCAGACAGACAGACCCAGT	TGCCGATGATGAACTTGTACT	152
ESTcapp88	(TTCGAT)4	GGATGATCTCTGCATTCTCAA	TCCATTTTGGGTTTGTGG	144
ESTcapp89	(TCTTTT)5	ATCTTCCCCAAATCAATGAAG	GCATCTCACCCAAGAAAAAGT	178
ESTcapp90	(CAG)8	CTCAGCAACCACAGATTCAAC	GGATGAACTCACTGGAGGATT	143
ESTcapp91	(CAG)7	ATTGAGACGCAGATGATGAAA	TTGGGCACGTTGTAATAAGAG	159
ESTcapp92	(CTTCCG)6	CCTTTCTAATGGTCGATCAGG	GAGAGCGAGAGATCTGAGGAG	142
ESTcapp93	(CAG)7	CGGAAGCTCCTGAGTTTGT	GAGGGTACATATGCAGCAGAG	144
ESTcapp94	(GCAAAC)5	ATTTAACGATTGACGGATGTG	ACTAGACCATCATGCACGAAC	148

Supplementary Table S3. List of 150 primer pairs designed. Fifty EST-SSR selected and tested in the present study are reported in italic. Polymorphic markers (14) with expected size are in bold. Monomorphic markers in our collection with expected size (13) are underlined.

Primer	Motif	Forward primer (5'-3')	Reverse primer (5'-3')	Expected size (bp)
ESTcapp95	(TCT)7	CACAACTCGCAATTCTGTTTT	AACGCCATAACCGACTCTAAC	147
ESTcapp96	(TCTC)10	GCCAAGACCTTTAGGTAGAGC	TCGTATCCAGGGATGAAATAA	143
ESTcapp97	(AAG)7	AAATGGGAGTGGAAGGAACTA	CGGAGAGAACATTTTTGATCTT	153
ESTcapp98	(TATG)5	CCATCATGTTCAGTGATGACA	CCCAATTAAAACCACATCAAA	157
ESTcapp99	(CCAG)6	CCCGAATGGTTAATCTCCTTA	CAAATGCCGAACACAGATAAC	127
ESTcapp100	(TCT)9	AGCAGAGCAGTAGAAACGACA	GACAGGCCCTTTTACTCAAAC	140
ESTcapp101	(CTT)8	GTGATCGATTGGGAAGAAATC	TACCTTTGAGACAAGCAGCAG	153
ESTcapp102	(TCAA)5	CATTTGATGAACACCATCTCC	TTTACCTCGTCGTTTTTAGGG	147
ESTcapp103	(TTGA)6	TCATACGCGAGAATTTTGAAG	GCACTCTTTGTCGTAGTCTGC	149
ESTcapp104	(ATG)7	TGTCGCTTTGATTTAGGTCAC	TCCTTCCACGTAAAGATGTCA	150
ESTcapp105	(CCTGCT)6	ATATTCCACCGTCCGATTTAG	ACGTCGTATTCCTCGTCTTCT	160
ESTcapp106	(CCTGCT)8	ACAAGCCTGAGGAGGATTTAG	GAAACACAAATTTGCTTTGGA	153
ESTcapp107	(TATA)5	TTTGCATTCCAACTTCATTTC	CGTTATGAGTGAAGAGGCTGA	143
ESTcapp108	(AGA)8	TGGAAGATTTTTCAGTCATCG	CTTTGGTATCAATGGCTTCCT	132
ESTcapp109	(AGA)8	CAAGCAAGTATGTGAGCCCTA	GCCATAATACCCAACTTCACA	154
ESTcapp110	(AGCATC)4	CAGTAGCATGAAGGGGATGTA	TGCATTAAAACATGAGAAGCA	150
ESTcapp111	(AGA)8	GGAGGAAGAATTGGATTATCG	CTATCCCAAGCAGCAATTACA	150
ESTcapp112	(ATAT)5	AAGCTTAACGCTGAGAAGAGG	ATACCCAAAAGTTCCACCATT	155
ESTcapp113	(CTT)8	GCCACCAGAGGTTTCTTCTAA	AAGGATTTTGTTTTGGTGGAG	148
ESTcapp114	(AAG)10	ATGAAGACAGCGACAATGAAG	TTGGACTCTCTGGTACAATGAA	159
ESTcapp115	(TCT)8	TTATCTGATTCGTTGGCTCTG	CAAAGAGAACCTTAGCCTGTG	167
ESTcapp116	(TCT)8	TGACAGATTCTAGCTGGAGCA	CCACAAACTCTCAAAGAGAACC	152
ESTcapp117	(ATAG)5	GCACAGCTTCAGGTTCTGATA	CCATTGGTATATAATCCACAAAA	217
ESTcapp118	(TGTA)6	GTTTCTTTCGCAAATCAACAT	CACACACACACACACACACAC	126

Supplementary Table S3. List of 150 primer pairs designed. Fifty EST-SSR selected and tested in the present study are reported in italic. Polymorphic markers (14) with expected size are in bold. Monomorphic markers in our collection with expected size (13) are underlined.

Primer	Motif	Forward primer (5'-3')	Reverse primer (5'-3')	Expected size (bp)
ESTcapp119	(CAG)8	GTCACAGCCTTTCTCTTCTCC	TTGGTACAAGAATCGGTTCAC	134
ESTcapp120	(CTCT)10	GCCGAATTGGTTAAATTCAGT	CCGTGAACCAAGCAATATAGA	153
ESTcapp121	(TCC)7	GGGAGATCGAACAAGAATCTC	GGATCTCTCCTGGAGCATAAG	151
ESTcapp122	(CCT)8	AGAGGAAAGCCCAGATTTGTA	TCAGCATACTGCCTAAATTCG	144
ESTcapp123	(CCT)7	TCCTCCTCAATCTCTTGTTCC	GTTTTGTTTGGAAGCTTCTGC	148
ESTcapp124	(CATA)5	ATGTACAGGATCGAGCAGACA	AAACAGGAGGAGAAAGTGGTG	129
ESTcapp125	(TTA)15	TGTTGATCCGAGCTTAGACTG	TCCAACCCTAAACTCCAATTC	184
ESTcapp126	(CAT)12	TGGAACATCGACTCTCTCTTG	GCCATCCTCTCCTGTACTCTC	157
ESTcapp127	(TAATAT)4	TGTGCCAAAGTCATTTTCAAC	TGGGAAGACATTGTACATCAAA	146
ESTcapp128	(AGA)7	AGACGAGCCATTGAGATTTTT	TATAATACTGGCGTGCTGCTC	151
ESTcapp129	(TAA)7	CGGAATAGTCCTCCCAAAATA	TTGAATGGAATGTCACGTTTT	153
ESTcapp130	(AAC)7	TTTCTGATTTTCAGGCAGCTA	TGATTTGGTTCGATTTTCTTG	148
ESTcapp131	(TCTC)5	CTCTCACACGAACACACACAC	GCCATTTGGAGAGAGAGAGAG	150
ESTcapp132	(TATG)5	AGGGACATATCTACCAATTCCA	AAGGGTTTGGTCATCAAATCTA	156
ESTcapp133	(TCC)7	CCCTACTACCAGTTTCCTCCA	AGGAGGAGGAGGAGGAGGT	150
ESTcapp134	(TCCT)5	CCGTTTTTCTCCATCATCTCT	AAAGAGGTCCAGAAAGCGATA	138
ESTcapp135	(GAGAAG)4	AGAGAAGCGAGACAAAAGGAA	ATCAAATTCGTCTGGACCTTT	153
ESTcapp136	(ATG)7	GGAACGAGTTTTGGAATTGAT	CCTGCAGCTACTCTGTTCATT	173
ESTcapp137	(GAA)7	GAAGCAGAAGAAGAGGAGGAA	GATTCAATGCCTTTACGTTTG	146
ESTcapp138	(CCTCCG)6	GGAAGCTAAGTTTGCTGCTCT	AGCAGAAATCCAGAACTCACC	169
ESTcapp139	(CGGAGG)4	GTTTCAGCTCCTCACGACATA	ATGTCTAAACCGCCGAGTAAC	140
ESTcapp140	(GCCTCC)7	CAGAGAGACGTGCTTGATTGT	AAAGGTTCGAATGTGAAATCC	150
ESTcapp141	(ATAT)5	TCTTCCTTTGCATCTGTGTTC	CGAGTTCTAGCATCACACACA	167
ESTcapp142	(GACGAT)6	TGTAAAGGTGAAGTTGCAGATG	AACCAACATGAGAATGGTCCT	150

Supplementary Table S3. List of 150 primer pairs designed. Fifty EST-SSR selected and tested in the present study are reported in italic. Polymorphic markers (14) with expected size are in bold. Monomorphic markers in our collection with expected size (13) are underlined.

Primer	Motif	Forward primer (5'-3')	Reverse primer (5'-3')	Expected size (bp)
ESTcapp143	(TCTTT)5	GCAAAGCGACCAGATTACAT	GGAAGCTTCTGAAACTCCATT	158
ESTcapp144	(AGGGAG)4	AGATCGTGACTATGACCGAGA	CTGCTTCTACTCCTGCTCCTT	156
ESTcapp145	(TTTC)7	GCTTGCTGATTCACACAGTTT	GTTCACTGAAAGTCCGGGTAT	148
ESTcapp146	(GCA)8	GTGCACAAAAGGATACGAGTG	ATCATAGGCCTTGGTTCTGTC	155
ESTcapp147	(TTC)7	TGTATCTCCAGTGACGCTCTT	AGAAACTGAAAAGGCAACTCC	151
ESTcapp148	(TTC)12	AAAGCTAAGGGAGAAGATCCA	TCTGCAAAAAGTTGCTCAGAT	147
ESTcapp149	(GCG)7	GCTTCAACAACAACAGCAGAT	ATTGAATTGCCCGTTAGTAGG	133
ESTcapp150	(CATATC)4	TTGGGCAATATAATGGTTTGA	ACGATTTCGAATAAGCTGCTA	149

Supplementary Table S4. *Capparis spinosa* collection used for the EST-SSR validation.

N°	Sample ID	Species	Origin	Population code used in DAPC analysis
1	CC AZ 03	*C. spinosa* subsp. *spinosa*	Azerbaijan	CC world
2	CC AZ 04	*C. spinosa* subsp. *spinosa*	Azerbaijan	CC world
3	CC AZ 05	*C. spinosa* subsp. *spinosa*	Azerbaijan	CC world
4	CC AZ 06	*C. spinosa* subsp. *spinosa*	Azerbaijan	CC world
5	CC AZ 07	*C. spinosa* subsp. *spinosa*	Azerbaijan	CC world
6	CC AZ 08	*C. spinosa* subsp. *spinosa*	Azerbaijan	CC world
7	CR CAL 01	*C. spinosa* subsp. *rupestris*	Italy - Calabria	CR Italy
8	CR CAL 02	*C. spinosa* subsp. *rupestris*	Italy - Calabria	CR Italy
9	CR CAL 03	*C. spinosa* subsp. *rupestris*	Italy - Calabria	CR Italy
10	CC CAM 02	*C. spinosa* subsp. *spinosa*	Italy - Sicily	CC Sicily
11	CC CAM 03	*C. spinosa* subsp. *spinosa*	Italy - Sicily	CC Sicily
12	CC CAM 05	*C. spinosa* subsp. *spinosa*	Italy - Sicily	CC Sicily
13	CR CBAS 01	*C. spinosa* subsp. *rupestris*	Italy - Basilicata	CR Italy
14	CR CBAS 02	*C. spinosa* subsp. *rupestris*	Italy - Basilicata	CR Italy
15	CR CBAS 03	*C. spinosa* subsp. *rupestris*	Italy - Basilicata	CR Italy
16	CR CEF 02	*C. spinosa* subsp. *rupestris*	Italy - Sicily	CR Sicily
17	CC CHINA 02	*C. spinosa* subsp. *spinosa*	China	CC world
18	CC CIPRO 01	*C. spinosa* subsp. *spinosa*	Cyprus	CC world
19	CC CIPRO 03	*C. spinosa* subsp. *spinosa*	Cyprus	CC world
20	CC CIPRO 04	*C. spinosa* subsp. *spinosa*	Cyprus	CC world
21	CC CIPRO 05	*C. spinosa* subsp. *spinosa*	Cyprus	CC world
22	CR FAV 01	*C. spinosa* subsp. *rupestris*	Italy - Sicily - Favignana island	CR Favignana
23	CR FAV 02	*C. spinosa* subsp. *rupestris*	Italy - Sicily - Favignana island	CR Favignana
24	CR FAV 05	*C. spinosa* subsp. *rupestris*	Italy - Sicily - Favignana island	CR Favignana
25	CR FAV 06	*C. spinosa* subsp. *rupestris*	Italy - Sicily - Favignana island	CR Favignana
26	CR LAM 01	*C. spinosa* subsp. *rupestris*	Italy - Sicily - Lampedusa island	-
27	CR LAR 02	*C. spinosa* subsp. *rupestris*	Italy - Sicily	CR Sicily
28	CR LAR 04	*C. spinosa* subsp. *rupestris*	Italy - Sicily	CR Sicily
29	CC MIL 03	*C. spinosa* subsp. *spinosa*	Italy - Sicily	CC Sicily
30	CC MIL 06	*C. spinosa* subsp. *spinosa*	Italy - Sicily	CC Sicily
31	CC MIL 08	*C. spinosa* subsp. *spinosa*	Italy - Sicily	CC Sicily
32	CC MIL 09	*C. spinosa* subsp. *spinosa*	Italy - Sicily	CC Sicily

Supplementary Table S4. *Capparis spinosa* collection used for the EST-SSR validation.

N°	Sample ID	Species	Origin	Population code used in DAPC analysis
33	CC MIR 04	*C. spinosa* subsp. *spinosa*	Italy - Sicily	CC Sicily
34	CC MOR 02	*C. spinosa* subsp. *spinosa*	Italy - Sicily	CC Sicily
35	CC MOR 03	*C. spinosa* subsp. *spinosa*	Italy - Sicily	CC Sicily
36	CR MOR 05	*C. spinosa* subsp. *rupestris*	Italy - Sicily	CR Sicily
37	CR MOR 06	*C. spinosa* subsp. *rupestris*	Italy - Sicily	CR Sicily
38	CC MOR 07	*C. spinosa* subsp. *spinosa*	Italy - Sicily	CR Sicily
39	CR MOR 07	*C. spinosa* subsp. *rupestris*	Italy - Sicily	CR Sicily
40	CR MOR 08	*C. spinosa* subsp. *rupestris*	Italy - Sicily	CR Sicily
41	CR MOR 09	*C. spinosa* subsp. *rupestris*	Italy - Sicily	CR Sicily
42	CR PAN 03	*C. spinosa* subsp. *rupestris*	Italy - Sicily - Pantelleria island	CR Pantelleria
43	CR PAN 06	*C. spinosa* subsp. *rupestris*	Italy - Sicily - Pantelleria island	CR Pantelleria
44	CR PAN 07	*C. spinosa* subsp. *rupestris*	Italy - Sicily - Pantelleria island	CR Pantelleria
45	CR PAN 09	*C. spinosa* subsp. *rupestris*	Italy - Sicily - Pantelleria island	CR Pantelleria
46	CR PAN 21	*C. spinosa* subsp. *rupestris*	Italy - Sicily - Pantelleria island	CR Pantelleria
47	CR PAN 61	*C. spinosa* subsp. *rupestris*	Italy - Sicily - Pantelleria island	CR Pantelleria
48	CC PCA 01	*C. spinosa* subsp. *spinosa*	Italy - Sicily	CC Sicily
49	CC PCA 03	*C. spinosa* subsp. *spinosa*	Italy - Sicily	CC Sicily
50	CC PCA 05	*C. spinosa* subsp. *spinosa*	Italy - Sicily	CC Sicily
51	CR ROMA 01	*C. spinosa* subsp. *rupestris*	Italy - Lazio	CR Italy
52	CR ROMA 02	*C. spinosa* subsp. *rupestris*	Italy - Lazio	CR Italy
53	CR ROMA 03	*C. spinosa* subsp. *rupestris*	Italy - Lazio	CR Italy
54	CR ROMA 05	*C. spinosa* subsp. *rupestris*	Italy - Lazio	CR Italy
55	CR ROMA 06	*C. spinosa* subsp. *rupestris*	Italy - Lazio	CR Italy
56	CR SAL 02	*C. spinosa* subsp. *rupestris*	Italy - Sicily - Salina island	CR Salina
57	CR SAL 04	*C. spinosa* subsp. *rupestris*	Italy - Sicily - Salina island	CR Salina
58	CR SAL 05	*C. spinosa* subsp. *rupestris*	Italy - Sicily - Salina island	CR Salina
59	CR SAL 07	*C. spinosa* subsp. *rupestris*	Italy - Sicily - Salina island	CR Salina
60	CR SAL 09	*C. spinosa* subsp. *rupestris*	Italy - Sicily - Salina island	CR Salina
61	CR SAL 10	*C. spinosa* subsp. *rupestris*	Italy - Sicily - Salina island	CR Salina
62	CR SIC 02	*C. spinosa* subsp. *rupestris*	Italy - Sicily	CR Sicily
63	CC SIC 04	*C. spinosa* subsp. *spinosa*	Italy - Sicily	CC Sicily
64	CR SIC 04	*C. spinosa* subsp. *rupestris*	Italy - Sicily	CR Sicily
65	CC SIC 08	*C. spinosa* subsp. *spinosa*	Italy - Sicily	CC Sicily
66	CC TOR 13	*C. spinosa* subsp. *spinosa*	Italy - Sicily	CC Sicily

Supplementary Table S4. *Capparis spinosa* collection used for the EST-SSR validation.

N°	Sample ID	Species	Origin	Population code used in DAPC analysis
67	CC TOR 02	*C. spinosa* subsp. *spinosa*	Italy - Sicily	CC Sicily
68	CC TOR 03	*C. spinosa* subsp. *spinosa*	Italy - Sicily	CC Sicily
69	CC TOR 04	*C. spinosa* subsp. *spinosa*	Italy - Sicily	CC Sicily
70	CR UST 01	*C. spinosa* subsp. *rupestris*	Italy - Sicily - Ustica island	CR Ustica
71	CR UST 04	*C. spinosa* subsp. *rupestris*	Italy - Sicily - Ustica island	CR Ustica
72	CR UST 07	*C. spinosa* subsp. *rupestris*	Italy - Sicily - Ustica island	CR Ustica
73	CR UST 13	*C. spinosa* subsp. *rupestris*	Italy - Sicily - Ustica island	CR Ustica
74	CR UST 14	*C. spinosa* subsp. *rupestris*	Italy - Sicily - Ustica island	CR Ustica
75	CR UST 15	*C. spinosa* subsp. *rupestris*	Italy - Sicily - Ustica island	CR Ustica

Supplementary Table S5. List of wild populations of *Capparis spinosa* selected for RNA-Seq analysis with the geologic substrate of provenance and geographic origin. Provinces are indicated in brackets: AG = Agrigento; CL = Caltanissetta.

Population ID	Substrate	Location	Latitude	Longitude
**MOR**	Limestone	Morello (CL)	37°29’47.91’’	14°8’42.95’’
**MIL**	Chalk	Milena (CL)	37°28’53.53”	13°42’2.66’’
**SIC**	Chalk	Siculiana (AG)	37°20’43.57’’	13°23’10.00’’

Supplementary Figure S1. Gene ontology (GO) classification. Annotated unigenes were visualized into 3 major categories: (**A**) Biological Processes; (**B**) Cellular Components; and (**C**) Molecular Function. The percentage of unigenes and count for each specific category is indicated in the plots.

A

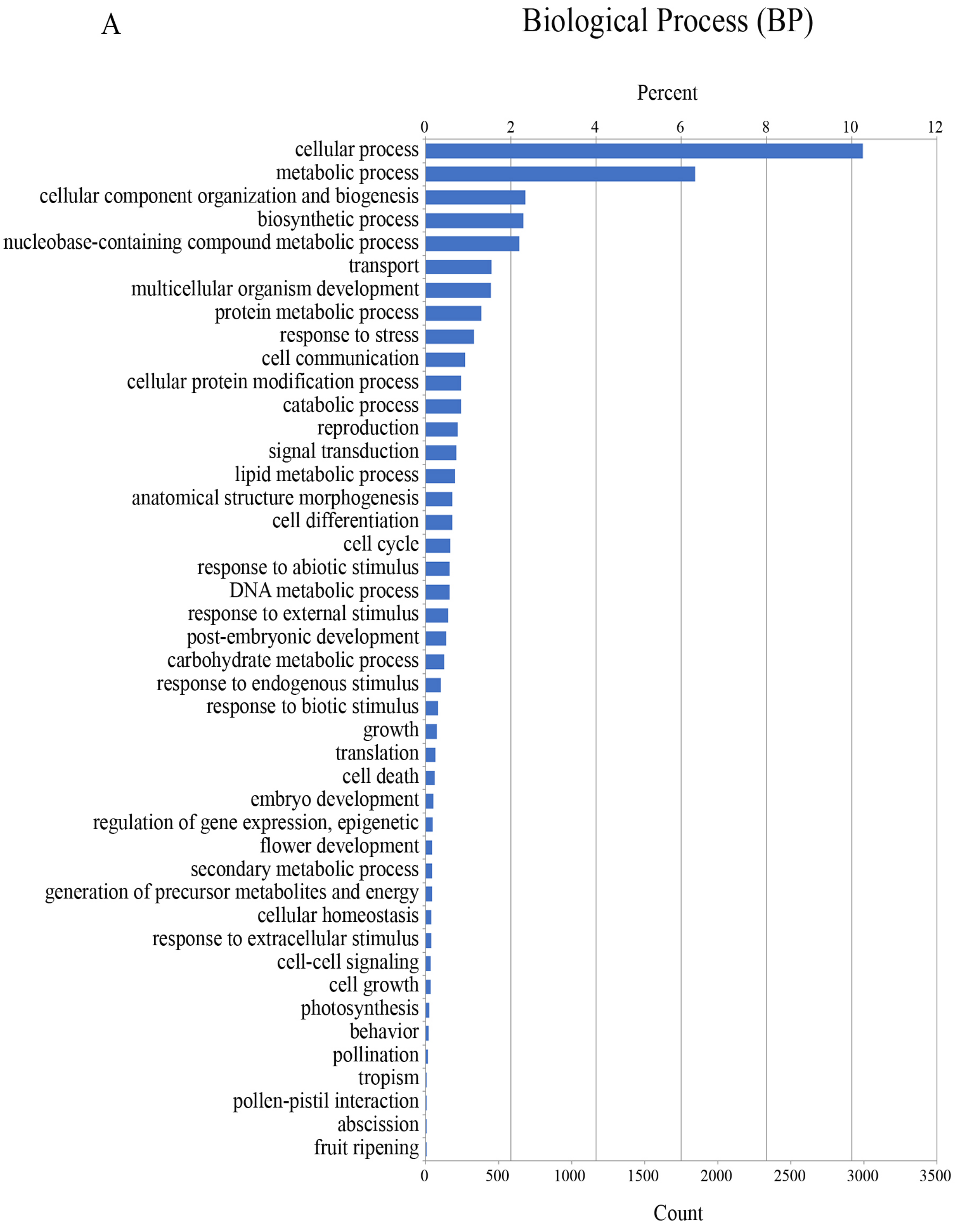

Supplementary Figure S1. Gene ontology (GO) classification. Annotated unigenes were visualized into 3 major categories: (**A**) Biological Processes; (**B**) Cellular Components; and (**C**) Molecular Function. The percentage of unigenes and count for each specific category is indicated in the plots.

B

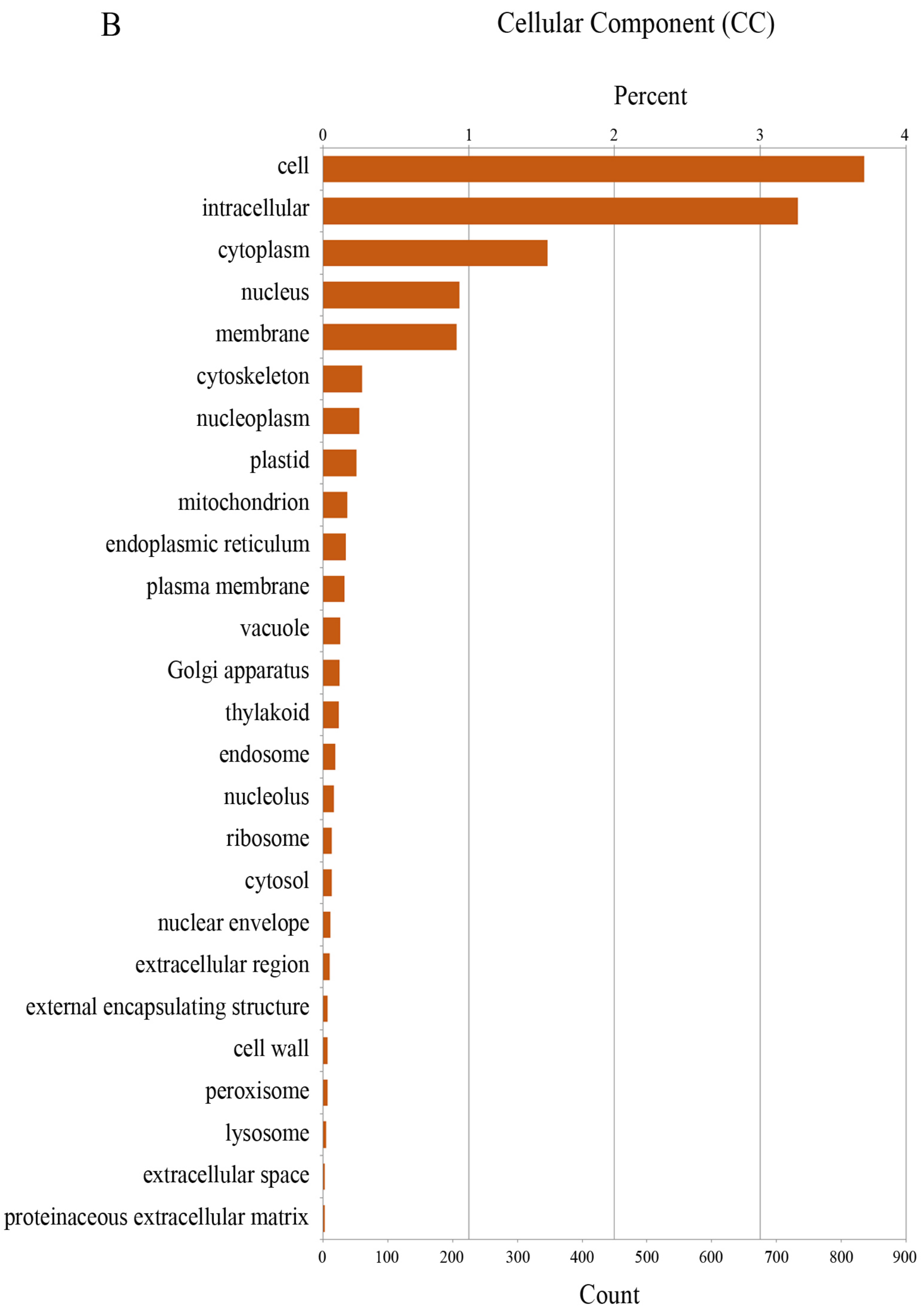

Supplementary Figure S1. Gene ontology (GO) classification. Annotated unigenes were visualized into 3 major categories: (**A**) Biological Processes; (**B**) Cellular Components; and (**C**) Molecular Function. The percentage of unigenes and count for each specific category is indicated in the plots.

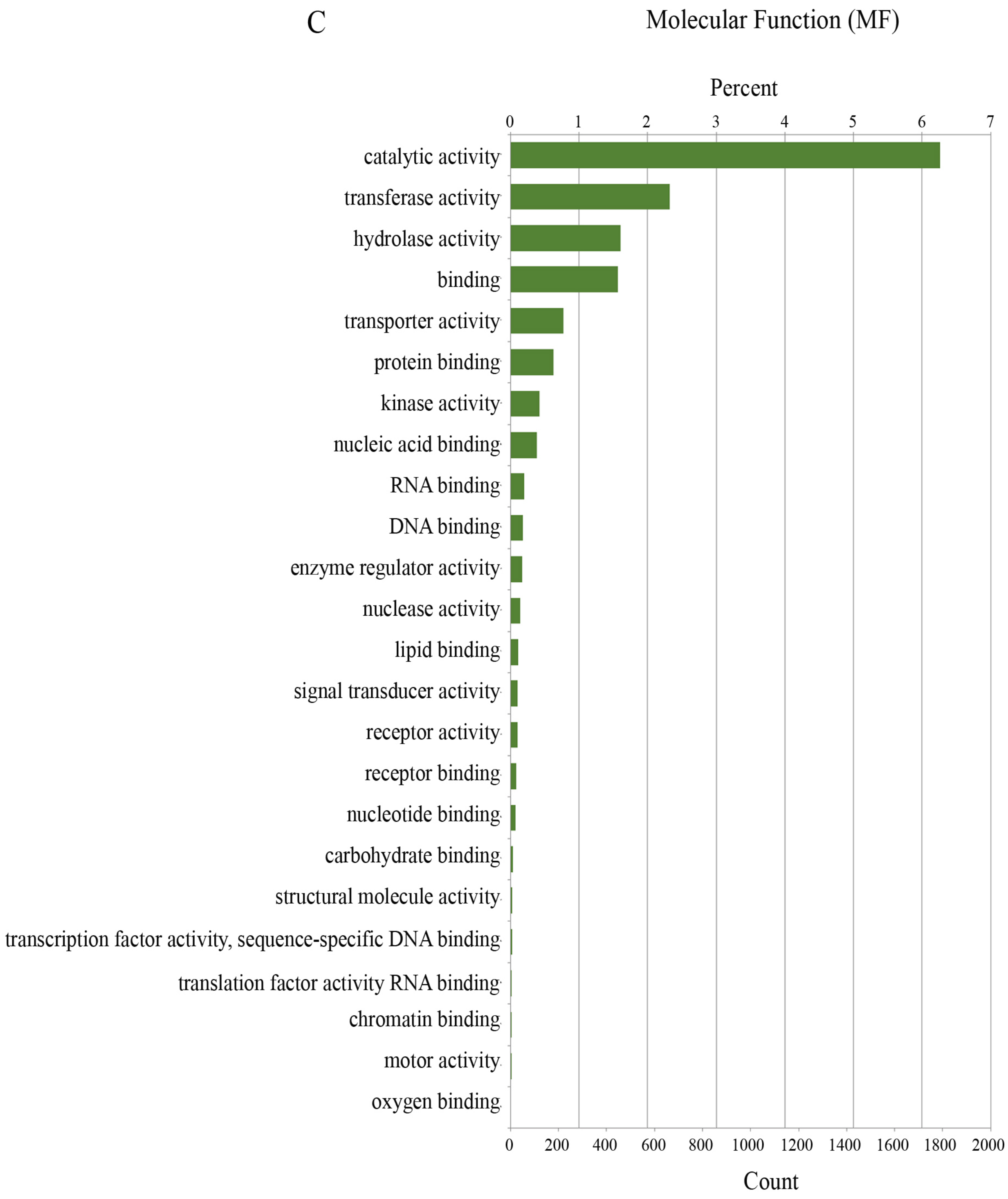

Supplementary Figure S2. KEGG analysis showing genes involved in sphingolipid metabolism pathways (**A**), oxidative phosphorylation (**B**), and photosynthesis (**C**) in *Capparis spinosa* leaf transcriptome (Enzyme Code - EC - identified are in green). Pathways were developed through KEGG Mapper, Search Pathway (https://www.genome.jp/kegg/tool/map_pathway1.html) [125,126,127,128]

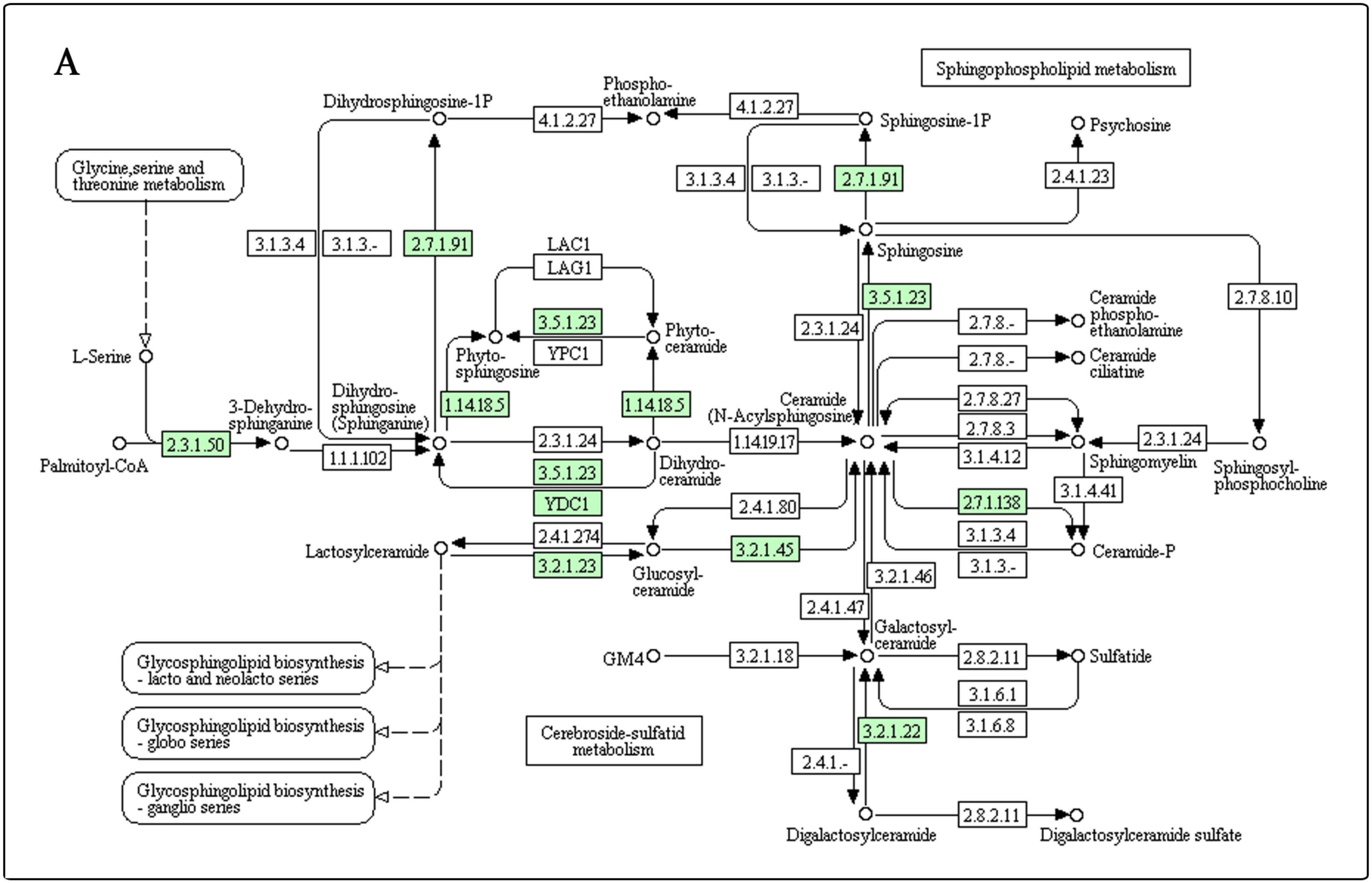

Supplementary Figure S2. KEGG analysis showing genes involved in sphingolipid metabolism pathways (**A**), oxidative phosphorylation (**B**), and photosynthesis (**C**) in *Capparis spinosa* leaf transcriptome (Enzyme Code - EC - identified are in green). Pathways were developed through KEGG Mapper, Search Pathway (https://www.genome.jp/kegg/tool/map_pathway1.html) [125,126,127,128]

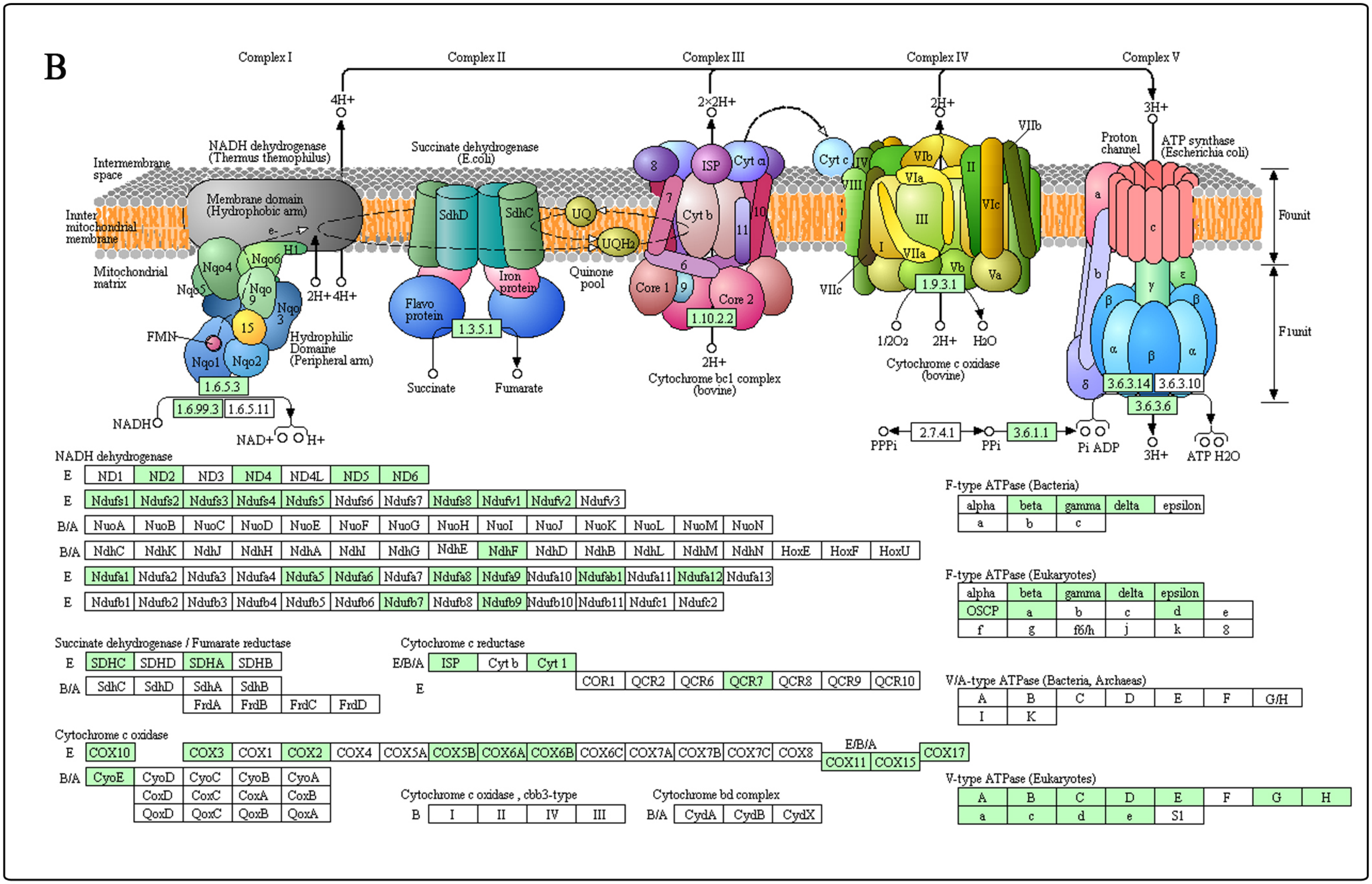

Supplementary Figure S2. KEGG analysis showing genes involved in sphingolipid metabolism pathways (**A**), oxidative phosphorylation (**B**), and photosynthesis (**C**) in *Capparis spinosa* leaf transcriptome (Enzyme Code - EC - identified are in green). Pathways were developed through KEGG Mapper, Search Pathway (https://www.genome.jp/kegg/tool/map_pathway1.html) [125,126,127,128]

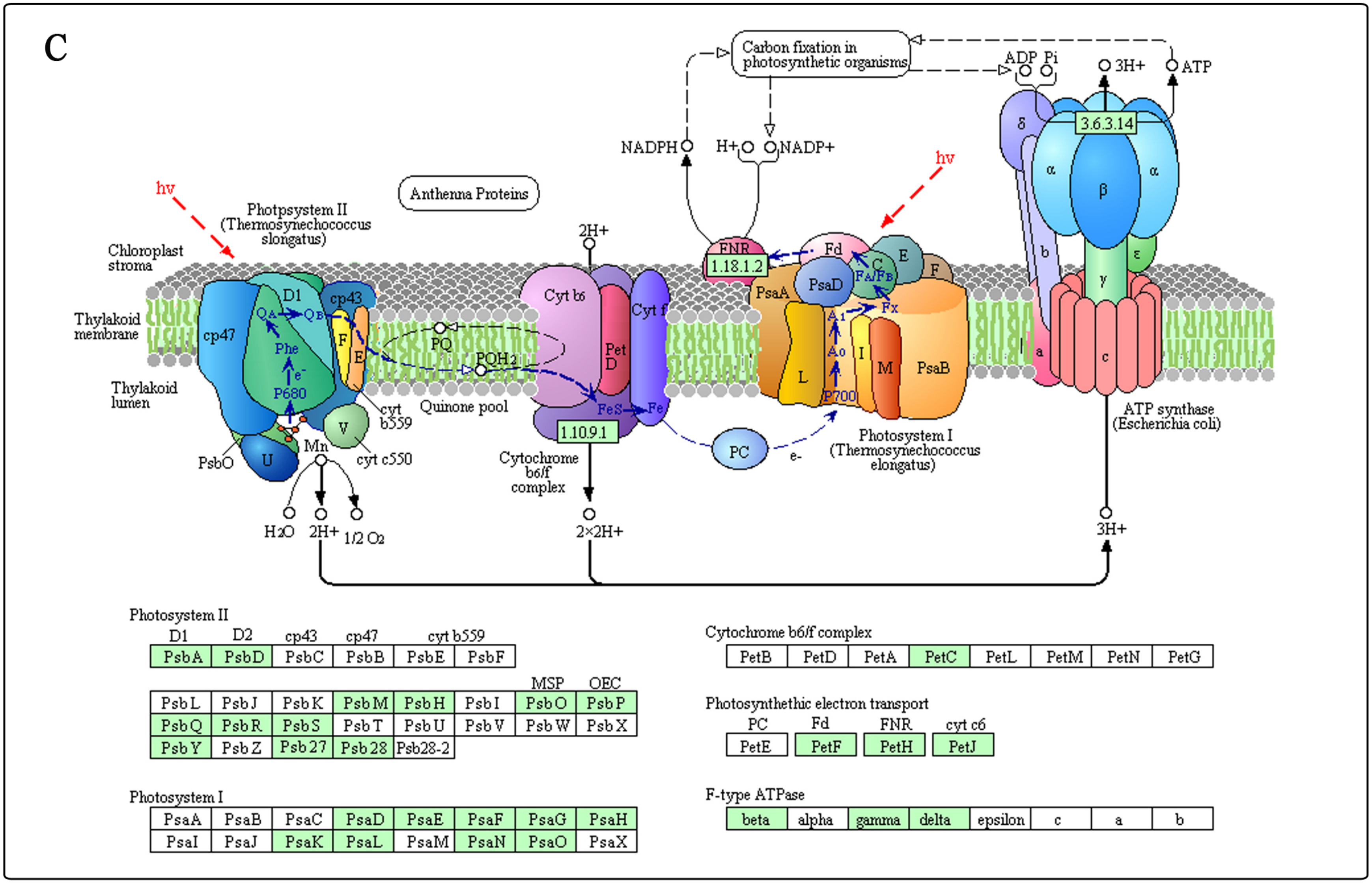

Supplement: Supplementary file 3 — Supplementary information [file 41598_2019_46613_MOESM3_ESM.pdf]
